# Isolation, Characterization, and Safety Evaluation of Human Skin-Derived Precursors from an Adherent Monolayer Culture System

**DOI:** 10.1155/2019/9194560

**Published:** 2019-08-19

**Authors:** Ru Dai, Wei Hua, Wei Chen, Lidan Xiong, Li Li, Yiming Li

**Affiliations:** ^1^Department of Dermatology, Ningbo First Hospital, Zhejiang University, No. 59, Liuting Street, Ningbo, Zhejiang 315010, China; ^2^Department of Dermatology, West China Hospital, Sichuan University, No. 37, Guo Xue Xiang, Chengdu, Sichuan 610041, China

## Abstract

**Background:**

Skin-derived precursors (SKPs) are promising candidates for regenerative medicine. Several studies have transcultured human SKPs (termed tSKPs) from fibroblasts (FBs) expanded in monolayer culture. Herein, we optimized the procedure by treating flasks with poly-2-hydroxyethyl methacrylate (poly-HEMA).

**Methods:**

tSKPs generated from our adherent monolayer culture system were investigated for protein expression and differentiation capacity. The aggregated cells and the proliferative cells within tSKP spheres were detected by mix-culturing FBs expressing two different fluorescent proteins and BrdU- or EdU-positive cells, respectively. To distinguish tSKPs from FBs, we compared their phenotypes and transcriptomes. The tumorigenicity of tSKPs and FBs was also assessed in our study.

**Results:**

tSKPs expressed Versican, Fibronectin, Vimentin, Sox2, and Nestin. Under appropriate stimuli, tSKPs could differentiate to mesenchymal or neural lineages. While these spheres were heterogeneous populations consisting of both proliferative and aggregated cells, the rate of proliferative cells correlated with a seeding density. tSKPs, isolated from FBs, were distinctive from FBs in cell cycle, marker expression, neural differentiation potential, and transcript profiles despite the two sharing partial similarity in certain properties. As for tumorigenesis, both tSKPs and FBs could be considered as nontumorigenic *ex vivo* and *in vivo*.

**Conclusion:**

tSKPs were heterogeneous populations presenting similar characteristics as traditional SKPs, while being different from FBs. The potential mixture of FBs in spheres did not affect the biosafety of tSKPs, as both of which had normal karyotype and nontumorigenicity. Taken together, we suggested tSKPs had potential applications in regenerative medicine.

## 1. Background

Skin-derived precursors (SKPs), first described in 2001 [1], are stem cells with self-renewal and multilineage differentiation capacity. Traditionally, SKPs are isolated according to the Nature Protocols [2], that is growing as floating spheres in suspending serum-free medium with basic fibroblast growth factor (bFGF) and epidermal growth factor (EGF). Many studies have stated that growing human SKPs (hSKPs) through a traditional method had many limitations such as relatively low yield, slow growth rate, and cell heterogeneity [3, 4]. Recently, several methods have been described to facilitate hSKP generation, including applying three-dimensional environment [5], employing nanofibrous scaffolds [6], suspending in stirred bioreactors [7], and establishing from human-induced pluripotent stem cells [8].

hSKPs are initially known for similar properties as neural crest stem cells (NCSCs) which can be cultured in two different forms of suspending neurospheres and adherent monolayer cultures; it is therefore unsurprising that several studies have reported to transculture hSKPs from monolayer fibroblast (FB) culture (henceforth termed tSKPs). Early in 2004, Joannides et al. pioneered the growth of hSKPs in attachment conditions supplemented with serum [9], the protocol of which was basically reproduced in the case with cheek skin by Yoshikawa et al. in 2013 [10]. In 2012, two independent groups successfully isolated multipotent hSKPs from primary monolayer FB culture [11, 12], and a more recent study from one of the above groups even reported to perform acidic stress on dermal FBs to include sphere expansion termed as pH-SKP cells [13]. Like hSKPs generated from primary dissociated dermis, these tSKPs shared similarities at protein expression, transcript profiles, and differentiation properties [12]. However, the identity of these tSKPs remains ambiguous. Since FBs isolated from human dermis also had multipotent adult stem cells with differentiation potential [14], scholars argued those tSKPs were clusters of dermal FBs rather than traditional hSKPs [15]. Moreover, NCSCs either in form of neurospheres or in form of monolayers were heterogeneous consisting of aggregated cells and proliferating cells even in very low-density cultures [16]. Fernandes et al. showed that traditional SKPs contained mixed cells when cultured at high cell densities; however, at low densities, only proliferative cells could be detected [17]. Therefore, we suppose that tSKPs are also heterogeneous populations and cell density may affect their composition. To date, there is no research investigating the composition of tSKP spheres as well as comparing the characteristics of tSKPs with primary adherent FBs. For further application, it is also essential to examine the biological safety and immunocompatibility of tSKPs although their traditional counterparts have been proved to be poorly immunogenic [18].

In the present study, we cultured dissociated dermal cells in a FB-adherent medium. Since spheres tended to adhere to plates while premature adherence would reduce cell yield [19], we coated plates with poly-2-hydroxyethyl methacrylate (poly-HEMA) to prevent adherence before seeding cells [20]. The acquired tSKPs were then characterized for their properties. A further investigation into the impact of cell density on tSKPs' composition was also performed. In addition, we compared the biological properties of tSKPs with primary FBs in immunocytochemistry, cell cycle, CD antigen expression, differentiation capacity, and RNA-sequencing (RNA-seq). For potential clinical application, we assessed the biosafety of tSKPs and FBs *ex vivo* and *in vivo*. This study provides strategy for tSKP isolation and evidence to distinguish tSKPs from preestablished primary FBs.

## 2. Methods

### 2.1. Isolation and Preparation of Primary FB Cultures from Human Foreskin Samples

Human skin samples were obtained from patients aged 18 to 45 years old undergoing circumcisions, who signed informed consent forms. Our project was conducted in accordance with the ethical guidelines of West China Hospital (Chengdu, China) and had Ethics Committee approval (No. 2017064A).

The culture procedure was performed according to the protocol described by Wenzel et al. with some modification [21]. Human foreskins were washed twice with cold phosphate buffer solution (PBS), cut into 4-6 mm^2^ pieces, washed again, and incubated in 0.25% (*v*/*v*) trypsin (HyClone, USA) overnight at 4°C followed by 15 minutes at 37°C. The epidermis was manually removed and the dermis was minced into 1-2 mm^2^ pieces followed by incubating in collagenase IX (Sigma, USA) for 2-3 hours at 37°C. The digested tissues were mechanically dissociated and filtered through a 70 *μ*m cell strainer (BD Falcon, USA) and then centrifuged at 1,500 rpm for 4 minutes. The supernatant was removed and the remaining cell pellet was seeded at 25,000 cells/mL into a FB-adherent medium ([Supplementary-material supplementary-material-1]). Cells (10 mL total medium) were cultured in a 25-cm^2^ tissue culture flask (BD Falcon, USA) in 37°C with the medium changed every 2-3 days. When reaching 80% confluency, FBs were passaged into next generation. FBs isolated from primary human skin were defined as P0 FBs, passaged once were defined as P1 FBs, and so forth.

### 2.2. Generation of tSKPs from Primary FB Cultures

FBs were incubated with 0.25% trypsin for 15 minutes at 37°C. Detached cells were washed twice with PBS and then transcultured into a SKP-proliferating medium ([Supplementary-material supplementary-material-1]) at the density of 25,000-40,000 cells/mL. We grew these cells in 25 cm^2^ tissue culture flasks coated with poly-HEMA (Sigma, USA) to reduce cell adherence and fed a 1-2 mL additional fresh SKP-proliferating medium containing all growth factors and supplements every 3 days. Cell growth was monitored and recorded by pictures every day. Five random fields were selected to estimate sphere' number and size. Fiji (version 1.0) was used to measure the diameter of the largest sphere in each field. tSKPs generated from P1 FBs were defined as P1 tSKPs, from P2 FBs were defined as P2 tSKPs, and so forth.

### 2.3. Immunocytochemistry

Cells were plated on slides and fixed in 4% (*m*/*v*) paraformaldehyde (PFA) for 30 minutes at room temperature. Then, cells were permeabilized with 0.25% (*v*/*v*) Triton X-100 for 10 minutes, followed by being blocked with 3% (*v*/*v*) bull serum albumin (BSA) in PBS for 30 minutes. Cells were incubated in primary antibody overnight at 4°C and secondary antibodies for 1 hour at room temperature. Cells were then washed three times for 5 minutes with PBS and stained with DAPI (Dojindo, Japan) for 1 minute before visualized under a fluorescence microscope (Olympus, Japan). Primary antibodies used in this study were anti-Versican Ab (Abcam, UK, 1 : 200), anti-Fibronectin Ab (Abcam, UK, 1 : 250), anti-Collagen I Ab (Abcam, UK, 1 : 500), anti-Vimentin Ab (Abcam, UK, 1 : 250), anti-Sox2 Ab (Abcam, UK, 1 : 200), anti-Nestin Ab (Abcam, UK, 1 : 200), anti-*α*-smooth muscle actin (*α*-SMA) Ab (Abcam, UK, 1 : 200), anti-Calponin Ab (Abcam, UK, 1 : 250), anti-S100*β* Ab (Abcam, UK, 1 : 200), anti-*β*III-tubulin Ab (Abcam, UK, 1 : 100), and anti-BrdU Ab (Abcam, UK, 1 : 200). The secondary antibody was Alexa Fluor® 488 donkey anti-mouse IgG (Abcam, UK, 1 : 500), Alexa Fluor® 594 goat anti-rabbit IgG (Abcam, UK, 1 : 500), and Alexa Fluor® 488 goat anti-rabbit IgG (Abcam, UK, 1 : 500). To estimate the percentage of cells expressing a given marker protein, three random fields were selected. The protocol was performed in three independent experiments.

### 2.4. Differentiation of tSKPs and FBs

For tSKPs, mature spheres generated from FBs were dissociated into single cells by collagenase XI and seeded at the density of 25,000 cells/mL into 6-well plates suspended with a SKP-adherent medium ([Supplementary-material supplementary-material-1]) until 60-70% confluency. As for FBs, cells were dissociated into single cells by 0.25% trypsin and seeded at the density of 25,000 cells/mL into 6-well culture dishes suspended with a FB-adherent medium ([Supplementary-material supplementary-material-1]) until 60-70% confluency.

For adipogenic and osteogenic differentiation, cells were then cultured in an adipogenic or osteogenic differentiation medium provided in the induced StemPro® Differentiation Kits (A1007001 and A1007201, Gibco, USA). After being induced for 21-28 days, cells were stained with Oil Red-O Solution (for adipocyte differentiation) or Alizarin Red Solution (for osteocyte differentiation). For directing into smooth muscle cells (SMCs), the basal medium was replaced by a SMC differentiation medium ([Supplementary-material supplementary-material-1]). At the end of a 28-day induced differentiation, cells were immunostained for SMC markers of *α*-SMA and Calponin (as described above). For neuron and Schwann cell differentiation, cells were seeded on laminin (Sigma, USA) and poly-D-lysine- (Sigma, USA) coated coverslips in 6-well culture dishes. When reaching 60-70% confluency, cells were transformed into a neuron or Schwann cell differentiation medium for 28 days ([Supplementary-material supplementary-material-1]). Immunocytochemical analysis was used to assess S100*β* or *β*III-tubulin expression (as described above).

### 2.5. Retroviral Transduction of FBs and Generation of tSKPs from Enhanced Green Fluorescent Protein- (EGFP-) and mCherry-Expressing FBs

P2 FBs were collected and seeded at the density of 1 × 10^5^ cells/well into 24-well culture dishes containing a 500 *μ*L FB-adherent medium. When the confluency reached 30%-40%, the medium was replaced by a fresh medium containing 5 *μ*g/mL polybrene (OBIO, China). Cells of each well were transduced with 7 *μ*L retroviruses expressing EGFP (OBIO, China) or 8 *μ*L retroviruses expressing mCherry, respectively, according to the protocol provided by the manufacturer (OBIO, China), and then incubated in 37°C, 5% CO_2_ condition. After transducing for 24 hours, cells were changed with a fresh medium without retroviruses. After 4 days under proliferating condition, transduced FBs were estimated under the fluorescent microscope to obtain successfully transduced population expressing either EGFP or mCherry.

EGFP- and mCherry-expressing cells were mixed at 1 : 1 ratio at the density of 1 × 10^3^ cells/mL, 5 × 10^3^ cells/mL, 1 × 10^4^ cells/mL, 2 × 10^4^ cells/mL, 5 × 10^4^ cells/mL, and 1 × 10^5^ cells/mL to generate tSKPs. The transculture methodology was described above. To identify the fluorescent protein expression level, tSKP spheres were visualized under a fluorescent microscope.

### 2.6. BrdU-Based and EdU-Based Proliferation Assay

Proliferative capacity of tSKP spheres was assessed by BrdU-based immunofluorescent analysis. Hereto, 10 *μ*mol/L BrdU was added to the tSKP cultures of different densities as described above at day 2. Afterwards, tSKP spheres were transformed into slides coated with poly-D-lysine and incubated overnight with a SKP-proliferating medium. Cells were fixed in 4% PFA for 15 minutes and permeabilized with 0.25% Triton X-100 for 10 minutes. Then, cells were treated with 2 N HCl for 30 minutes at 37°C followed by 30 minutes in 0.1 M borate buffer. The following steps were operated as the protocol mentioned above.

tSKP proliferation was also quantified by EdU labeling using the Cell-Light™ EdU Cell Proliferation Kit (RiboBio, China) and following the protocol described by the manufacturer. Briefly, 50 *μ*M EdU was added to the tSKP cultures of different densities as described above at day 2, and cells were cultured for 2 hours. Then, we collected tSKP spheres, digested them into single cells, and fixed cells with 4% PFA. To detect EdU incorporation, cells were treated with 2 mg/mL of glycine and permeabilized with 0.5% Triton X-100. Then, 500 *μ*L freshly prepared staining solution (provided by the manufacturer) was added, and cells were then incubated for 10 minutes followed by permeabilizing with 0.5% Triton once again. Finally, EdU-based fluorescence was analyzed by flow cytometer (BD, USA).

### 2.7. RNA Extraction and qRT-PCR

Total RNA was extracted from cells using Trizol (Ambion, USA) and then was converted to cDNA using the iScript™ cDNA Synthesis Kit (Bio-Rad, USA) according to the manufacturer's instructions. qRT-PCR was carried out using the CFX Connect™ Real-Time PCR Detection System (Bio-Rad, USA) with SsoAdvanced™ Universal SYBR® Green Supermix (Bio-Rad, USA). The primers are described in [Supplementary-material supplementary-material-1]. For a quantitative measurement, *β*-actin was used as an endogenous control and the RT-PCR signal of the experimental RNA was measured in relation to the signal of the control. The relative mRNA expression of each gene was calculated using the *ΔΔ*Ct method [22, 23]. Three independent biological experiments and three technical replicates were performed.

### 2.8. Cell Cycle Analysis

Cell cycle analysis was performed using PI staining and flow cytometry. At time point of 48, 72, and 120 hours, 10^6^ tSKPs and FBs were harvested and digested into single cells. Cells were washed in 1 mL cold PBS and centrifuged. The supernatant was discarded before adding 70% (*v*/*v*) ice-cold ethanol to fix cells at 4°C overnight. Cells were centrifuged and washed twice with cold PBS. Each sample was added with 100 *μ*L RNAase (KeyGen BioTech, China) and then incubated in the 37°C water bath for 1 hour before staining with 400 *μ*L PI (KeyGen BioTech, China). The samples were kept in darkness at 4°C and then analyzed with flow cytometer.

### 2.9. Cell Surface Marker Expression

The cell surface marker expression of tSKPs and FBs was analyzed using the BD Stemflow™ hMSC Analysis Kit (BD, USA) according to the manufacturer's instruction. Briefly, we detached cells into single cells, washed with PBS, and resuspended at a concentration of 10^7^ cells/mL. Samples were added with CD antibodies (anti-CD90, anti-CD73, anti-CD105, anti-CD44, and negative cocktail of anti-CD19, anti-CD45, anti-CD11b, anti-CD34, and HLA-DR) and incubated in darkness on ice for 30 minutes before being analyzed on flow cytometer.

### 2.10. Karyotyping

Karyotypes of tSKPs at day 3 and FBs at day 2 of P5 and P10, respectively, were analyzed. Colcemid (Gibco, USA) was added to a final concentration of 0.25 *μ*g/mL for 3 hours at 37°C. The precipitate of samples was collected after digesting by trypsin. Samples were then treated with 0.075 M hypotonic potassium chloride and 3 : 1 methanol and acetic acid fixative according to the standard cytogenetic protocol. Metaphase chromosomes were prepared by dropping the fixed cell suspension onto precleaned slides above an alcohol lamp, aged at 65°C overnight before G-banding with 0.25% trypsin and Giemsa's staining. Each sample was analyzed at least for 20 metaphases using Leica chromosome analysis software.

### 2.11. Major Histocompatibility Complex Analysis

The expression of HLA-I and HLA-DR of tSKPs and FBs of P5 and P10 was analyzed on the level of phenotype and mRNA through a flow cytometer and a PCR Detection System according to the methods mentioned above.

### 2.12. Transplantation of tSKPs and/or FBs in Severe Combined Immune-Deficient (SCID) Mice

All animal studies were performed under the guidelines of the institutional animal care. Both tSKPs and FBs of P5 and P10 were collected for transplantation.

Initially, tSKPs and FBs were labelled with EGFP to examine their survival *in vivo*. The EGFP-labelled tSKPs and FBs were prepared as mentioned above. The cell pellets (2 × 10^6^ cells) were resuspended in 200 *μ*L Hanks, respectively. Hanks containing tSKPs or FBs were subcutaneously injected into the buttock skin of SCID mice, and the mice were sacrificed after 2 weeks. The skin was cooled with liquid nitrogen and cut into frozen sections as soon as possible followed by being observed under an inverted fluorescence microscope.

SCID mice aged 6-8 weeks were bought from Vital River Co. LTD, Beijing. A total of 32 SCID mice were randomly assigned to eight groups as follows: blank, Hanks, P5 FBs, P5 tSKPs, P5 FBs+tSKPs (1 : 1 cell ratio), P10 FBs, P10 tSKPs, and P10 FBs+tSKPs (1 : 1 cell ratio). The detailed information is presented in [Supplementary-material supplementary-material-1]. In the buttock skin of the SCID mice, tSKPs, FBs, or a mixture of tSKPs and FBs was injected at 2-week intervals (at the beginning of weeks 1, 3, 5, 7, and 9). In short, the mice were transplanted subcutaneously with 2 million cells in five doses, a total of 10 million cells per animal. All the SCID mice were bred under specific-pathogen-free conditions with a 12-hour light-dark cycle. The general conditions of mice including injection sites, mental status, diet, and defecation were monitored daily. Mice were injected for 9 weeks and sacrificed 24 hours after their last treatment. Skins (back), brains, hearts, lungs, livers, spleens, and kidneys were examined macroscopically and histologically. The peripheral blood samples of different groups were also collected to analyze the total number of CD3+, CD4+, and CD8+ T cells and the ratio of CD4+/CD8+ T cells by a flow cytometer.

### 2.13. RNA-seq

cDNA library preparation was performed at Novogene, Beijing. Total RNA was extracted from samples using Trizol, and mRNA was purified from total RNA using oligo (dT) magnetic beads. The mRNA was broken into short fragments in fragmentation buffer. Taking mRNA as a template, first strand cDNA was synthesized using random hexamers. Second strand cDNA was subsequently synthesized in the condition of dNTPs, DNA polymerase I, and buffer. The cDNA was purified with AMPure XP beads and then performed end reparation and 3′-end single nucleotid A addition. At last, the resulting fragments were screened by AMPure XP beads and enriched by PCR amplification. The quality of library products was determined on the Agilent Bioanalyzer 2100 system, and the qualified products were used for sequencing on Illumina HiSeq™ 2000. FBs were considered as the control, and tSKPs were the treatment.

Original image data generated from HiSeq was transferred into sequenced reads through base calling, and these sequences were defined as “raw reads.” The resulting sequences were filtered as follows: remove adaptor sequences, N sequences, and low-quality sequences. The remaining reads were mapped to the human genome using Tophat v2.0.12, and no more than 2 mismatches were allowed during the mapping read procedure. The gene expression level was presented by using the RPKM method, which was calculated based on the length of the gene and sequencing discrepancies. HTSeq v0.6.1 was used to count the read numbers mapped to each gene. RPKM values could be used for comparing the difference of gene expression in two conditions. DEGSeq v1.12.0 was used to analyze gene expression difference of samples. “∣log_2_(Fold change)∣ > 1 and *q* value < 0.005” were used to identify differentially expressed genes (DEGs) as the threshold.

Gene ontology (GO) enrichment analysis of DEGs that are significantly enriched in GO terms using GOseq (Release 2.23). The corresponding biological functions related to the DEGs were also presented. The calculated *p* value was adjusted through Benjamini and Hochberg's approach, using an adjusted *p* < 0.05 as a threshold. GO terms with adjusted *p* < 0.05 were regarded as significantly enriched by DEGs. Kyoto encyclopedia of genes and genomes (KEGG) enrichment analysis provided differential expression genes that were statistical enrichment in KEGG pathways, using KOBAS v2.0.

### 2.14. Statistical Analysis

All data are presented as mean ± standard deviation (SD). Comparisons were performed with Student's *t*-tests or the Friedman M test. Student's *t*-test was used for comparisons between two groups, and ANOVA was performed for multiple comparisons. The Friedman M test was used for nonnormal distributed parameters. The significant level was set to *p* < 0.05.

## 3. Results

### 3.1. tSKPs Can Be Routinely Generated from Human Dermal FBs

**Figure 1:**
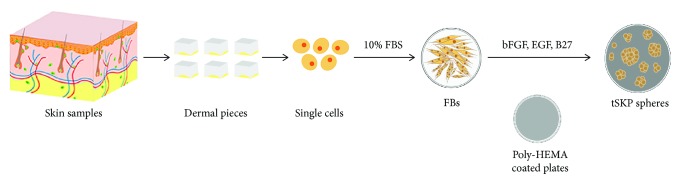
tSKP isolation protocols. Skin samples were cut into pieces with epidermis removed, followed by enzymatic digestion and mechanical dissociation into single cells. Dermal cells were seeded into a FB-adherent medium containing 10% FBS; mature FBs were then transferred into a SKP-proliferating medium. After an average of 7 days, mature spheres could be observed.

**Figure 2:**
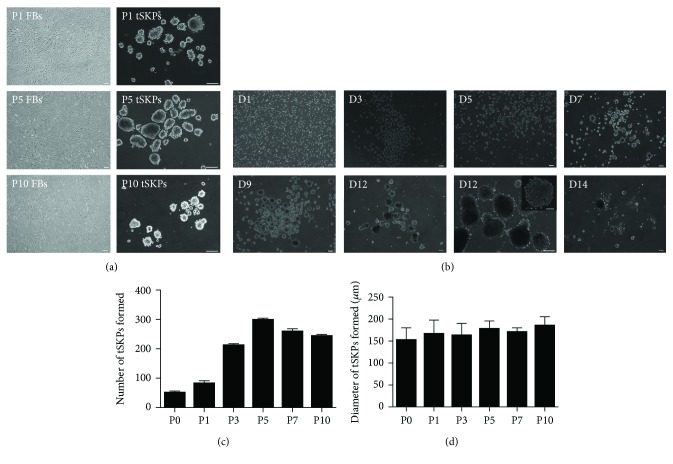
tSKPs were generated from primary adherent FB cultures. (a) Phase contrast images of monolayer adherent FBs at P1, P5, and P10 (left panel) and tSKPs derived from the left FB culture (right panel). Note the characteristic morphology, which was very similar to primary SKPs. Typical SKP spheres had been successfully generated from P1, P5, and P10 FBs in this study. (b) Formation of tSKP spheres derived from P3 FBs from day 1 to 14 in culture. A typical SKP sphere appeared after approximately 7 days. Inset in day 12 shows the typical morphology of a mature sphere. Scale bars: 100 *μ*m. (c) Measurement of tSKP number from FBs at different passages at day 7. (d) Measurement of a tSKP size from FBs at different passages at day 7.

According to our protocol of treating plates with Poly-HEMA (Figure 1), tSKPs could be generated from primary adherent FBs at P1, P5, and P10 (Figure 2(a)), and these resulting spheres were morphologically similar to primary hSKPs as described by Toma et al. [24]. Focused on tSKPs generated from P3 FBs, we first identified phase bright, spherical colonies after about 3 to 5 days in SKP proliferation media (Figure 2(b)). The mature spheres of tSKPs took an average of 7 days to form (Figure 2(b)), which was shorter than traditional cultured SKPs as reported [2]. At day 12-14, tSKP spheres grew larger, the central cores of spheres began to darken, and some spheres even adhered to the plates (Figure 2(b)), which indicated that these spheres should be passaged. The spheroid number and size from FBs with different passages were investigated to assess tSKP-forming ability. The number of tSKPs increased with FB generation, while decreased when subcultured more than 5 times (Figure 2(c)). The results of spheroid size revealed no obvious variance among tSKPs from FBs at different generations (Figure 2(d)). Compared with regular SKPs (regular SKPs: 134 ± 5.9 *μ*m) described by Toma et al. [24], the average diameter of tSKPs (range from 152 to 185 *μ*m) was a little larger regardless of any generations.

**Figure 3:**
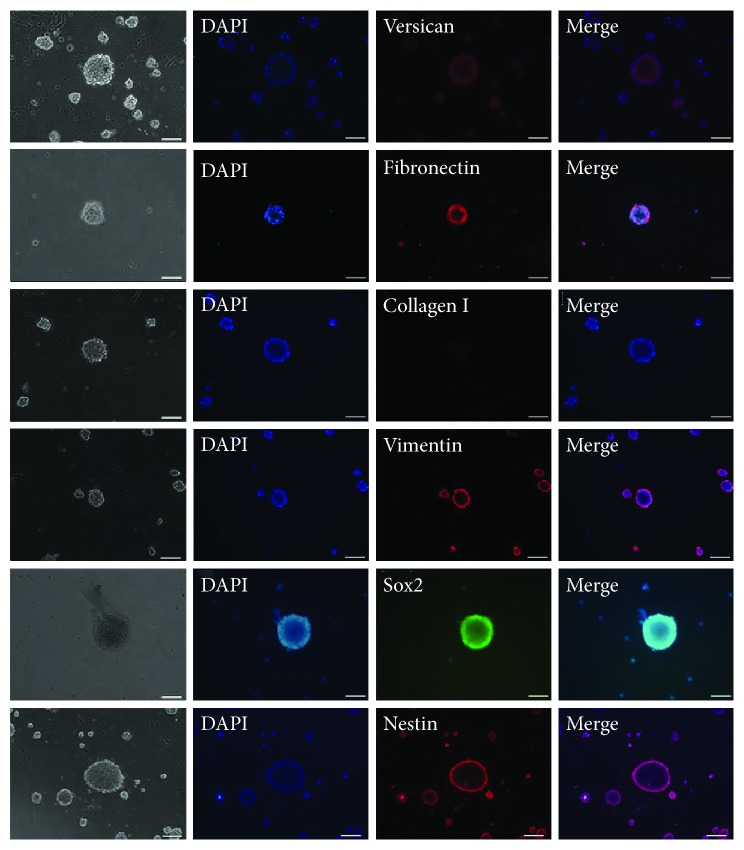
Immunocytochemical analysis of tSKPs. tSKPs expressed Versican (red), Fibronectin (red), Vimentin (red), Sox2 (green), and Nestin (red), while did not expressed Collagen I (red). Nuclei of all the cells were counterstained with DAPI (blue). Scale bars: 100 *μ*m.

To further confirm the similarity between tSKP spheres and regular SKP spheres, the protein expression of tSKPs was analyzed by immunocytochemistry. As shown in Figure 3, cells were positive for Versican, Fibronectin, Vimentin, Sox2, and Nestin, which was similar to traditional SKPs [24, 25]. However, tSKPs did not express FB characteristic markers Collagen I, suggesting tSKP spheres were different from primary adherent FBs.

### 3.2. tSKPs from FBs Can Be Directed into Multiple Differentiation Pathways

Since traditional SKPs hold differentiation potential in both neural and mesodermal cell types [24], we therefore investigated whether tSKPs could also be differentiated into these lineages. tSKPs generated from P3 to P5 FBs were examined for their differentiation potential.

**Figure 4:**
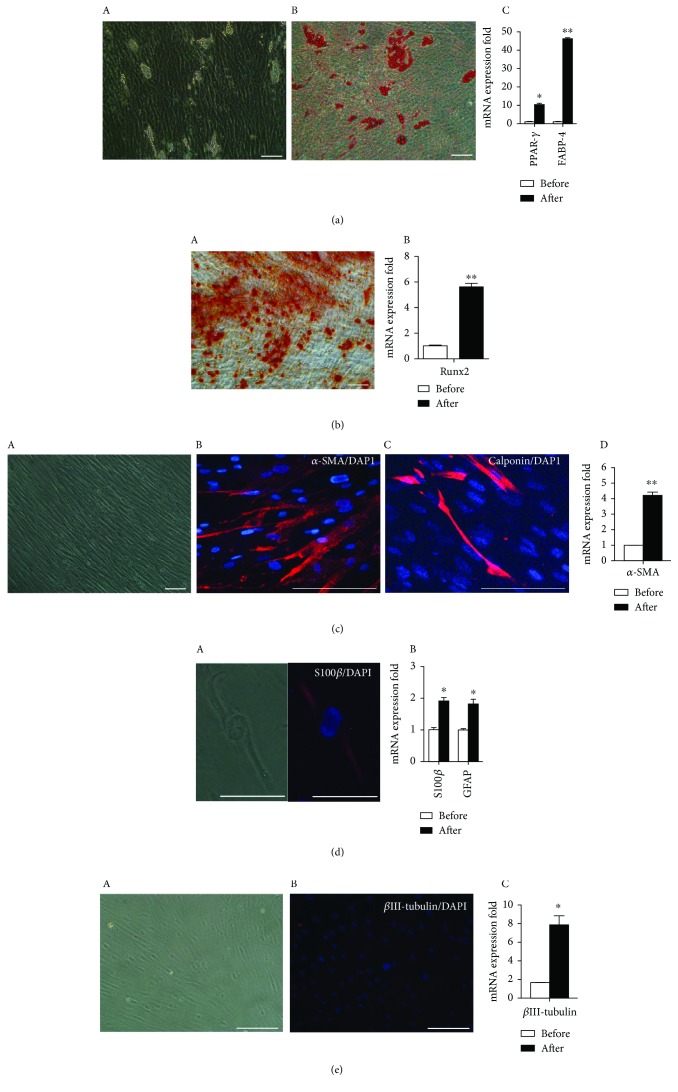
Multiple differentiation potentials of tSKPs generated from primary adherent FBs. (a) tSKPs could differentiate into adipocytes after induction for 28 days. (A) Phase contrast imaging showed lipid droplet inclusions, (B) and these lipids were positive staining for Oil Red-O. (C) The qRT-PCR results showed that PPAR-*γ* and FABP-4 was significantly increased after induction. (b) tSKPs could differentiate into osteocytes after induction for 28 days. (A) Calcium deposition was detected by Alizarin Red staining. (B) The qRT-PCR results showed that Runx2 was significantly increased after induction. (c) tSKPs could differentiate into smooth muscle cells after induction for 28 days. (A) Phase contrast imaging revealed the morphology of elongated and spindle appearance. The immunocytochemistry analysis showed that cells were positive for (B) *α*-SMA and (C) Calponin. (D) The qRT-PCR results showed that *α*-SMA was significantly increased after induction. (d) tSKPs could differentiate into Schwann cells after induction for 28 days. (A) The immunofluorescence staining revealed the expression of the Schwann cell differentiation marker of S100*β*. (B) The qRT-PCR results showed that S100*β* and GFAP were significantly increased after induction. (e) After induction in a neuron differentiation medium for 28 days, (A) immunofluorescence staining detected that cells were negative for *β*III-tubulin, while the mRNA expression of *β*III-tubulin was significantly increased after induction. ^∗^*p* < 0.05, ^∗∗^*p* < 0.01. Scale bars: 100 *μ*m.

After being cultured in an adipogenic medium for 28 days, lip droplets were detected in cultures from tSKPs. Oil Red-O staining indicated these cells exhibited the adipogenic phenotype (Figure 4(a)). The results of qRT-PCR analysis also confirmed that the mRNA expression of peroxisome proliferator-activated receptor-*γ* (PPAR-*γ*) and fatty acid binding protein-4 (FABP-4) was significantly elevated after being induced for 28 days (Figure 4(a)).

After being cultured in an osteogenic medium for 28 days, Alizarin Red staining indicated intracellular calcium deposition in cultures from tSKPs (Figure 4(b)). Furthermore, qRT-PCR analysis also showed that the mRNA expression of Runx2 increased significantly after induction (Figure 4(b)).

After a 28-day induction in a SMC differentiation medium, cells presented an elongated and spindle morphology. The immunocytochemistry revealed an expression of SMC markers of *α*-SMA and Calponin in cells being directed from tSKPs (Figure 4(c)). qRT-PCR showed the mRNA expression of *α*-SMA increased significantly after 4 weeks of induction (Figure 4(c)).

To examine whether tSKPs held the capacity of neurogenesis, cells were directed into neurons and Schwann cells in respective media. After 28 days in corresponding differentiation media, immunofluorescence staining revealed the expression of the Schwann cell differentiation marker of S100*β* (Figure 4(d)), while being negative for the early neuronal marker *β*III-tubulin (Figure 4(e)). However, the qRT-PCR results showed that both Schwann cell's markers of S100*β* and glial fibrillary acid protein (GFAP) (Figure 4(d)) and neuronal marker of *β*III-tubulin were significantly increased at a transcriptional level after induction (Figure 4(e)).

However, tSKPs isolated from FBs at high passage (P10) reduced multipotency. These tSKPs were incapable of neurogenetic differentiation although they could still be directed to adipocytes, osteocytes, and SMCs (Fig. [Supplementary-material supplementary-material-1]).

### 3.3. tSKPs Are Heterogeneous Cytospheres Consisting of Both Aggregated Cells and Proliferating Cells

**Figure 5:**
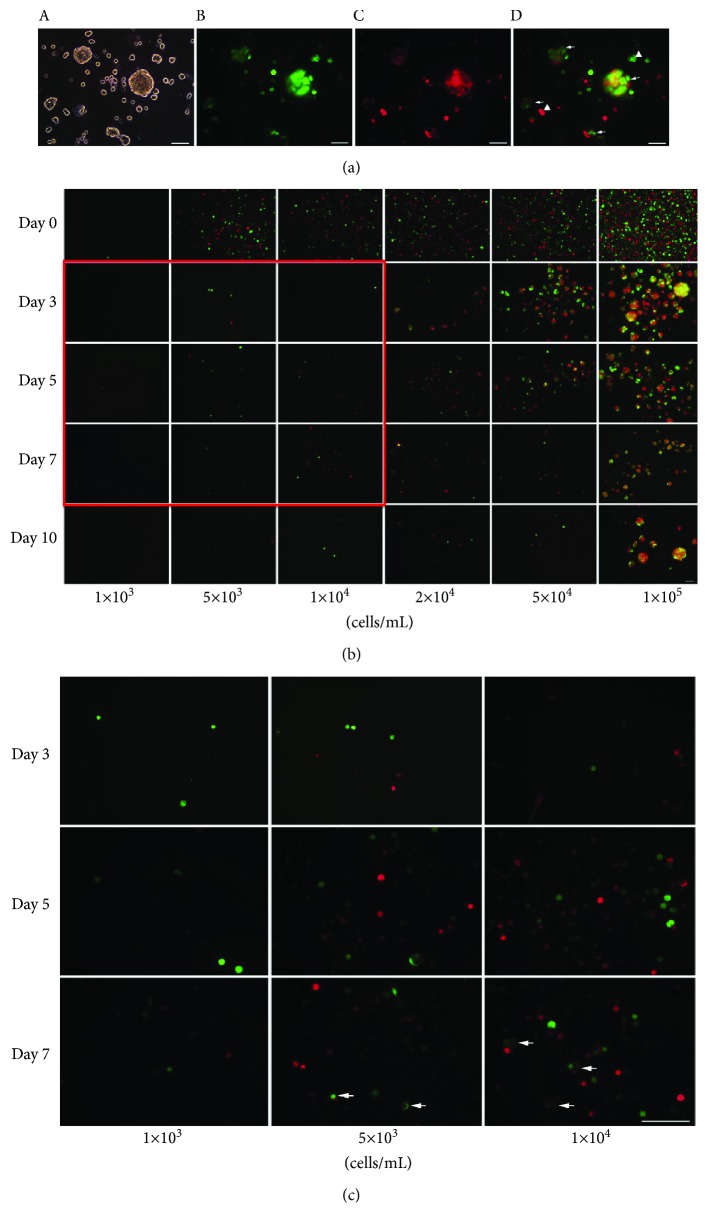
tSKP spheres generated from FBs contain aggregated cells. (a) tSKPs generated from mixed FBs expressing either EGFP or mCherry equally with a seeding density of 2‐4 × 10^4^ cells/mL. Phase contrast (A) and fluorescence photomicrograph (D) of tSKPs five days after transculture. Immunofluorescence detection of EGFP- (green) (B) and mCherry-positive cells (red) (C) in tSKP spheres, respectively. Arrows indicated spheres containing both EGFP- and mCherry-positive cells, and arrowheads showed spheres consisted of cells expressing either EGFP or mCherry alone. (b) Formation of tSKP spheres derived from mixed-FB plating at different densities of 1 × 10^3^, 5 × 10^3^, 1 × 10^4^, 2 × 10^4^, 5 × 10^4^, and 1 × 10^5^ cells/mL. (c) Enlarge the red box of (b). Arrows showed the spheres formed at a density of 5 × 10^3^ and 1 × 10^4^ cells/mL consisted of both EGFP- and mCherry-positive cells in day 7. Scale bars: 100 *μ*m.

To investigate whether tSKP spheres contained aggregated cell, we transduced FBs with retroviruses coding for either EGFP or mCherry. After 96 hours under proliferating conditions, FBs positive for EGFP (green) or mCherry (red) were observed under immunofluorescence (Fig. [Supplementary-material supplementary-material-1]). We then mixed FBs expressing either EGFP or mCherry at a 1 : 1 ratio and transcultured tSKPs in a SKP proliferation medium at the density of 2‐4 × 10^4^ cells/mL. Five days after transculturing, it was found that almost all tSKP spheres coexpressed EGFP and mCherry, and only very few cytospheres expressed EGFP or mCherry alone (Figure 5(a)). To further investigate whether the seeding density could affect the spheres' component, we transcultured tSKPs from mixed FBs at different densities of 1 × 10^3^, 5 × 10^3^, 1 × 10^4^, 2 × 10^4^, 5 × 10^4^, and 1 × 10^5^ cells/mL and recorded sphere status on a daily base. Our results showed that the higher the cell density, the faster their growth. Moreover, cell densities higher than 2 × 10^4^ cell/mL resulted in forming clusters as early as in day 3, and these clusters mostly coexpressed EGFP and mCherry (Figure 5(b)) indicating that spheres were mainly from cell aggregation. Moreover, spheres with double expressions at the densities of 5 × 10^3^ and 1 × 10^4^ cell/mL were observed at day 5 (Figure 5(c)). When plated at an ultralow density of 1 × 10^3^ cell/mL, the growth rate of spheres was extremely slow. During a 10-day transculture, only small spheres were observed while no mature spheres formed (Figure 5(c)). However, these small spheres expressed EGFP or mCherry alone, revealing that no cell aggregation was involved in clusters.

**Figure 6:**
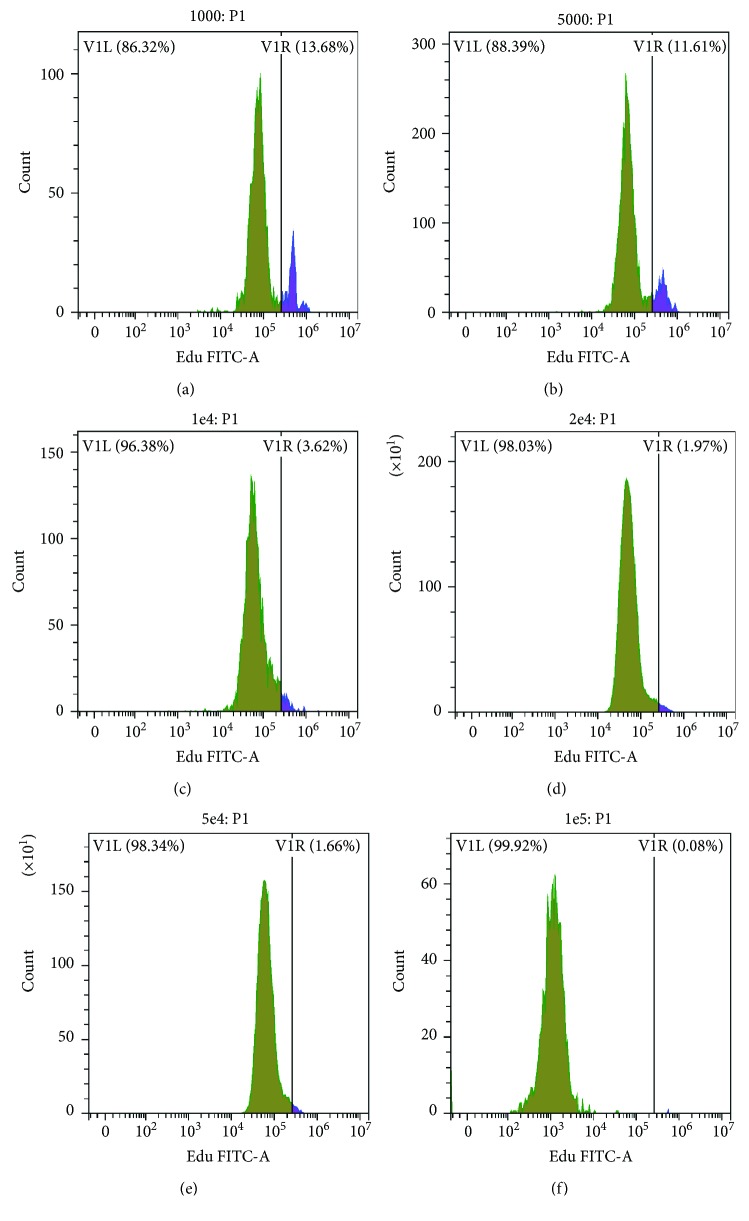
The percentage of proliferative cells within tSKP spheres, represented by EdU-positive cells. (a) 1 × 10^3^ cells/mL: 13.68%. (b) 5 × 10^3^ cells/mL: 11.61%. (c) 1 × 10^4^ cells/mL: 3.62%. (d) 2 × 10^4^ cells/ml: 1.97%. (e) 5 × 10^4^ cells/mL: 1.66%. (f) 1 × 10^5^ cells/mL: 0.08%.

To confirm tSKP spheres contained proliferative cells, BrdU was added into a SKP growth medium 1 day after plating and BrdU-positive cells were examined under immunofluorescence. The result showed that BrdU-positive cells could be detected within tSKP spheres (Fig. [Supplementary-material supplementary-material-1]), implying the existence of proliferative cells. To further explore the relationship between the plating density and the percentage of proliferative cells within spheres, we added EdU into a SKP growth medium at different densities of 1 × 10^3^, 5 × 10^3^, 1 × 10^4^, 2 × 10^4^, 5 × 10^4^, and 1 × 10^5^ cells/mL, respectively, and sorted EdU-positive cells by flow cytometer. We found that EdU-positive cells varied with the initial density: the higher the initial density, the lower the percentage of proliferative cells. The results of flow cytometer showed that the percentage of proliferative cells was 13.68%, 11.61%, 3.62%, 1.97%, 1.66%, and 0.08% at an initial density of 1 × 10^3^, 5 × 10^3^, 1 × 10^4^, 2 × 10^4^, 5 × 10^4^, and 1 × 10^5^ cells/mL accordingly (Figure 6).

### 3.4. FBs Are Distinctive from tSKPs, although the Two Share Several Similarities in Certain Characteristics

As described above, tSKP spheres were heterogeneous populations consisting of both aggregated and proliferative cells. Moreover, aggregated cells contributed largely to sphere formation. Therefore, we doubted whether the cytospheres were simply clusters of aggregated FBs rather than tSKPs. To answer it, we compared the biological properties and transcriptomes of FB-derived tSKPs with the corresponding FBs to investigate whether FBs had the same characteristics as tSKPs.

**Figure 7:**
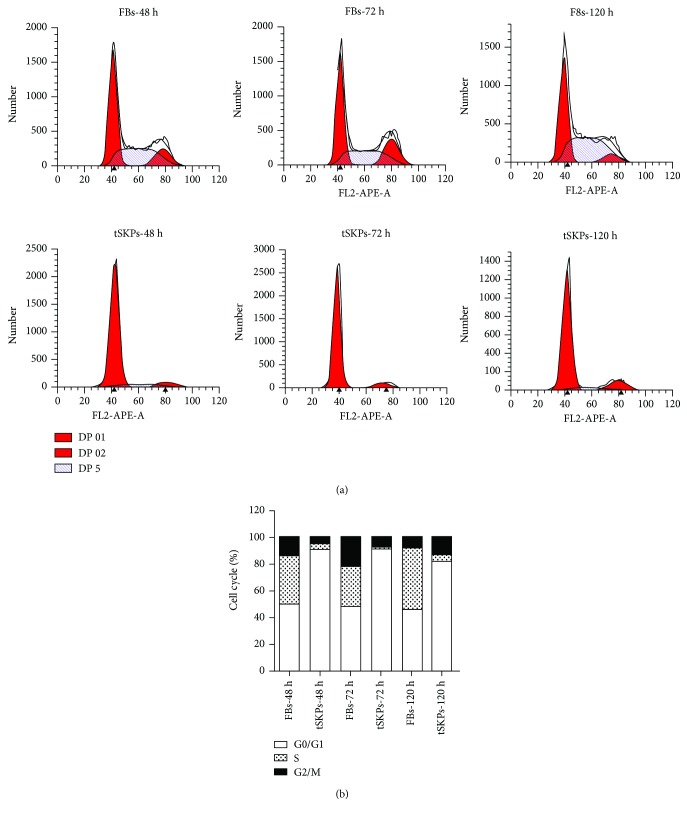
Cell cycle analysis results. FBs and tSKPs demonstrated different cell cycle pattern. The majority of tSKPs were in the G1 phase, while FBs were equally distributed in the G1 phase and the S+G2 phase. At each time point of 48, 72, and 120 hours, the proliferation index (S+G2/M) of FBs was still higher than tSKPs. The percentage of tSKPs in the S+G2 phase increased gradually.

The results of cell cycle analysis are shown in Figure 7. FBs and tSKPs demonstrated different cell cycle pattern. As for tSKPs, the majority of cells were in the G1 phase at each time point. For FBs, cells in the G1 phase and the S+G2 phase were equally distributed. However, we found that the percentage of tSKPs in the S+G2 phase slowly increased over time, although the proliferation index (S+G2/M) was still lower than FBs at the same time point, indicating tSKPs maintained a relatively slow growth rate as reported by Toma et al. [24].

**Figure 8:**
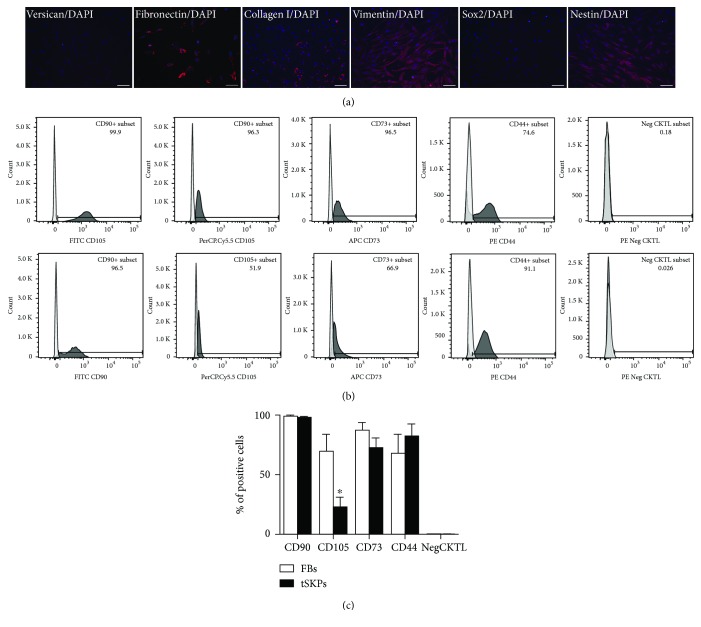
Characterization of primary adherent FBs and tSKPs. (a) Immunocytochemical of FBs. FBs expressed Fibronectin (red), Collagen I (red), Vimentin (red), and Nestin (red) and rarely expressed Versican (red), while were negative for Sox2 (red). Nuclei of all the cells were counterstained with DAPI (blue). (b) Cell surface marker expression of FBs (upper panel) and tSKPs (lower panel). Both FBs and tSKPs expressed CD90, CD105, CD73, and CD44, while lacking expression of negative cocktails. The expression rate of CD105 in FBs was significantly higher than in tSKPs (*p* < 0.05). (c) Histogram of the CD antigen expression. The percentage of FB expressed CD105 was significantly increased versus tSKPs. ^∗^*p* < 0.05. Scale bars: 100 *μ*m.

The results of immunocytochemistry showed that FBs expressed Fibronectin, Collagen I, Vimentin, and Nestin and rarely express Versican, while were negative for Sox2 (Figure 8(a)). The CD antigen expression analysis is shown in Figures 8(b) and 8(c). Both FBs and tSKPs expressed CD90, CD105, CD73, and CD44, while lacking expression of negative cocktail of CD19, CD45, CD11b, CD34, and HLA-DR (Figure 8(b)). The expression rate of CD105 in FBs was significantly higher than in tSKPs (*p* < 0.05) (Figure 8(c)).

**Figure 9:**
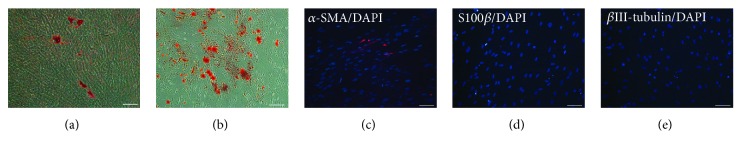
Multiple differentiation potentials of primary adherent FBs. (a) The result of Oil Red-O staining after a 28-day adipogenic induction detected lipid droplets. (b) The result of Alizarin Red staining after a 28-day osteogenic induction detected calcium deposition. (c) The result of immunofluorescence staining detected *α*-SMA-positive cells. (d) No S100*β*-positive cells were detected after a 28-day induction for Schwann cells. (e) No *β*III-tubulin-positive cells were detected after a 28-day induction for neurons.

To investigate whether FBs held differentiation potential, FBs were also cultured in differentiation media. After a 28-day induction, lipid droplets (Figure 9(a)) and calcified matrix (Figure 9(b)) were observed in cultures from FBs; however, the number of induced lipids and calcified matrix was less compared with cultures from tSKPs. Immunofluorescence analysis of SMC-induced cells showed positive staining for *α*-SMA (Figure 9(c)). However, as for neural-induced differentiation, cells were positive neither for Schwann cell's markers of S100*β* (Figure 9(d)) nor for neuronal markers of *β*III-tubulin (Figure 9(e)).

### 3.5. RNA-seq Analysis of tSKPs and FBs

**Figure 10:**
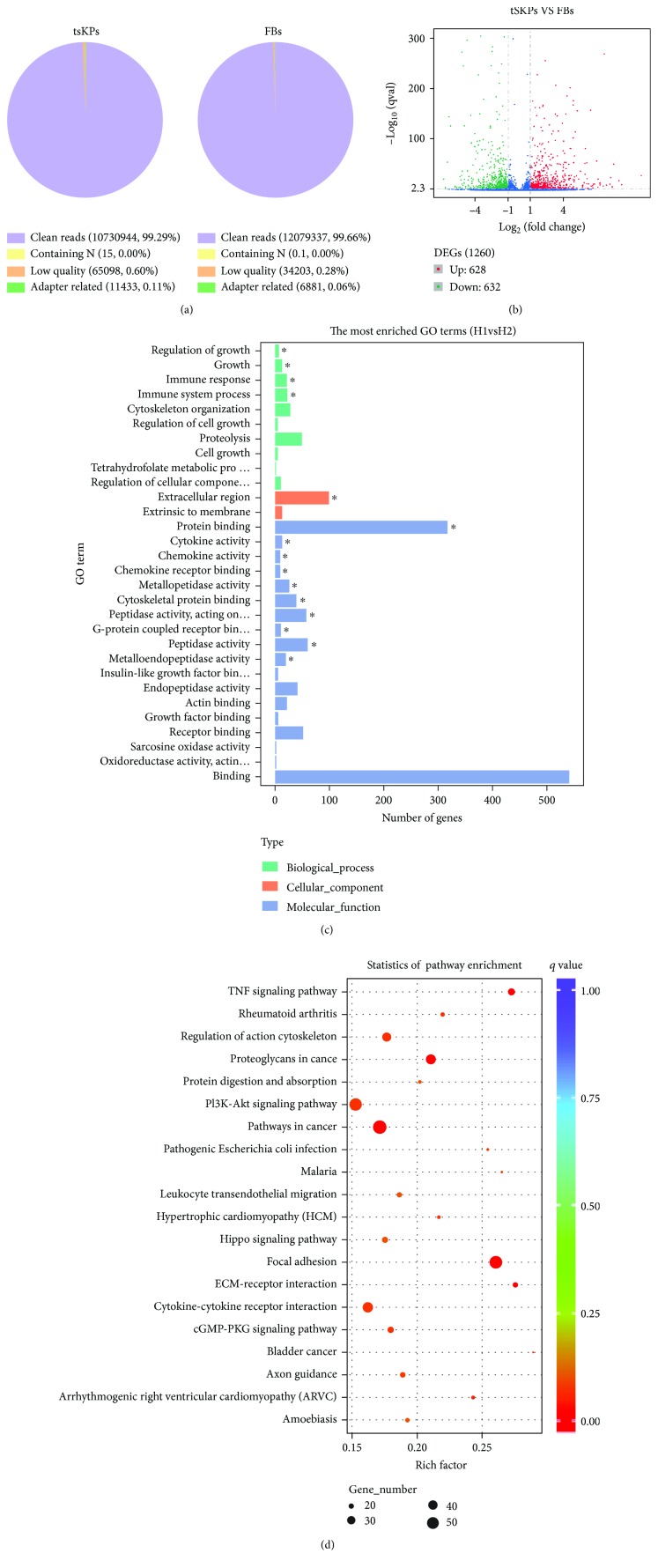
Results of RNA-seq of tSKPs and FBs. (a) Classification of raw reads in tSKPs and FBs. (b) Scattered plot of DEGs identified between tSKPs and FBs. (c) The top 30 enriched GO terms in the three main domains: biological process (10), cellular component (2), and molecular function (18). ^∗^Significantly enriched GO term. (d) Scattered plot of DEGs enriched KEGG pathways.

To explore potential mechanisms involved in the transculturing process, we performed RNA-seq to investigate RNA profiles in tSKPs and FBs (the detailed information of RNA-seq data was provided on GEO with the record of GSE133190). After filtering the only adaptor sequence, N-containing sequences, and low-quality sequences, each RNA-seq library still generated more than 10 million clean reads. The percentage of clean reads of each library was 99.29% and 99.66%, respectively (Figure 10(a)), and 96.82% and 97.85% of the remaining sequences matched to the human genome (Table 1).

A total of 1,260 DEGs were identified between tSKPs or FBs, with 628 upregulated and 632 downregulated (Figure 10(b)). The 1,260 DEGs could be categorized into 2,028 GO terms. In the three main domain of biological process, cellular component, and molecular function, the top 30 enriched GO terms are listed in Figure 10(c). Among these groups, 18 GO terms were in the domain of molecular function, which were mostly related to cellular binding, cytokine or chemokine activity, and catalytic activity; 2 GO terms were in the domain of cellular component, which were related to the extracellular region and extrinsic to membrane; 10 GO terms were related to biological process, which were associated with cellular growth and immune regulation. The full lists of up and down DEGs are presented in [Supplementary-material supplementary-material-1].

KEGG analysis identified the pathways where DEGs were significantly enriched, which was beneficial for further understanding DEG biological functions. The top 20 enriched KEGG pathways are shown in Figure 10(d), and we identified 5 significantly enriched pathways, which was focal adhesion (*p* = 2.13 × 10^−6^), TNF signaling pathway (*p* = 0.000988), proteoglycans in cancer (*p* = 0.002355), ECM-receptor interaction (*p* = 0.003310), and pathways in cancer (*p* = 0.014627), respectively.

The regulation at a transcriptional level is also essential for the gene expression. Transcription factor (TF) achieves gene regulation information by binding to a specific upstream nucleotide sequence. The analysis of TF identified significantly varied TFs in DEGs, helping to further understand possible mechanisms in the transculturing process. The differentially expressed TFs with strong evidence and their functions are listed in Table 2.

### 3.6. qRT-PCR for Data Validation

**Figure 11:**
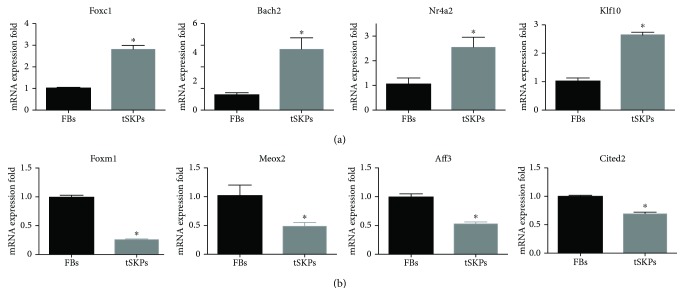
qRT-PCR results. (a) Foxc1, Bach2, Nr4a2, and Klf10 significantly upregulated in tSKPs. (b) Foxm1, Meox2, Aff3, and Cited2 significantly downregulated in tSKPs. ^∗^*p* < 0.05.

Several main TFs with ∣log_2_(Fold change)∣ > 2 were selected to verify the RNA-seq data by qRT-PCR. As shown in Figure 11, 4 TFs (Foxc1, Bach2, Nr4a2, and Klf10) were significantly upregulated in tSKPs and 4 TFs (Foxm1, Meox2, Aff3, and Cited2) were significantly downregulated in tSKPs. These results were highly consistent with RNA-seq, which confirmed that RNA-seq could provide reliable data for mRNA differential expression analysis.

### 3.7. tSKPs Generated from FBs Have Biological Safety

tSKPs generated from FBs shared similar properties with primary SKPs, which enabled tSKPs, a promising candidate for regenerative medicine. However, in any transplantation scenario, immunocompatibility should be investigated. de Kock et al. showed traditional hSKPs were poorly immunogenic and could modulate the allogeneic immune response [18]. In our study, tSKPs were generated from FBs and presented as heterogeneous spheres, which might contain previous FBs. As such, the biosafety of both FBs and tSKPs was investigated in this study.

**Figure 12:**
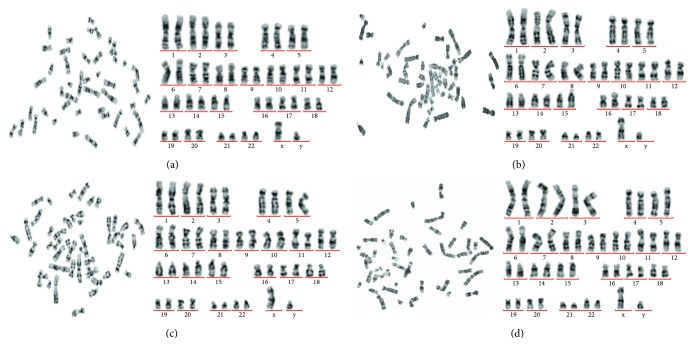
G-banding of metaphase chromosomes. The left panel shows the metaphase spread, and the right panel shows the ordered chromosomal pairs. (a) P5 FBs. (b) P5 tSKPs. (c) P10 FBs. (d) P10 tSKPs.

Using a G-banding set-up, the results of karyotype showed that both P5 and P10 FBs and tSKPs presented a normal man karyotype, 46 XY (Figure 12), without inversions, deletions, duplications, interfusions, or ring chromosomes.

**Figure 13:**
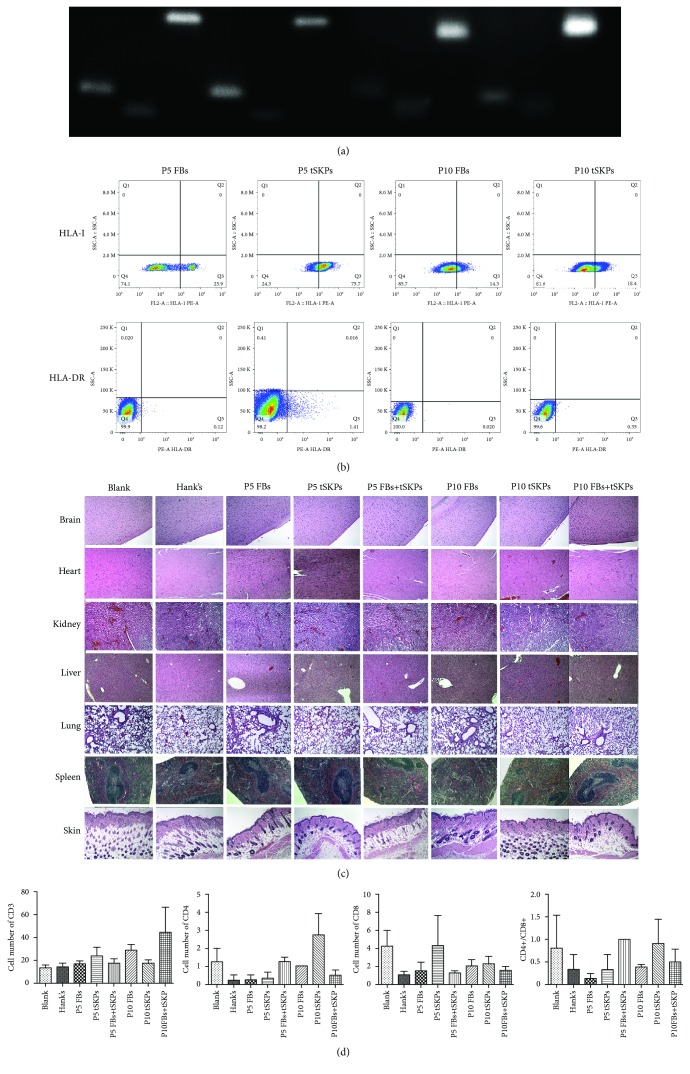
Analysis of biological safety for FBs and tSKPs. (a) RT-PCR analysis for the expression of HLA-I, HLA-DR, and, as a control, GADPH in FBs and tSKPs that had been passaged five and ten times. (b) Flow cytometer analysis for the surface marker expression of HLA-I, HLA-DR, and, as a control, GADPH in FBs and tSKPs that had been passaged five and ten times. (c) Effect of FBs and tSKPs to SCID mice on anatomic pathology. HE staining revealed that all organs from different groups showed normal histology. (d) Effect of FBs and tSKPs to SCID mice on the subpopulation of T lymphocytes. No significant change in the subpopulation of T lymphocytes. The values are expressed as mean ± SD.

HLA-I and HLA-DR expressions were determined in both FBs and tSKPs of P5 and P10, using RT-PCR and flow cytometer. At the transcriptional level, both P5 samples expressed HLA-I and were weakly positive for HLA-DR, while P10 samples were weakly positive for both HLA-I and HLA-DR (Figure 13(a)). At the protein level, the expression of HLA-I in P5 FBs, P5 tSKPs, P10 FBs, and P10 tSKPs was 75.7%, 25.9%, 18.4%, and 14.3%, respectively, which showed that the HLA-I expression was downregulated from P5 to P10 as well as from FBs to tSKPs; however, HLA-DR was not detected in all samples (Figure 13(b)).

Two weeks after transplanted into SCID mice, both tSKPs and FBs could survive as EGFP-labelled tSKPs and FBs could be identified in frozen sections of the dermis and subcutaneous layers (Fig. [Supplementary-material supplementary-material-1]). Then, we transplanted FBs, tSKPs, or mixtures from both P5 and P10 into SCID mice, and no unscheduled death was observed during the experiment, except for one mouse in the group of P5 tSKPs died one day before sacrifice. All mice were in good condition with normal appetite, and no infection, vomit, or abnormal excrement was observed, and were sacrificed after 9 weeks of experiment. There was no significant change or mass in organs macroscopically (Fig. [Supplementary-material supplementary-material-1]). In histological, the brain, heart, kidney, liver, lung, spleen, and skin from each group revealed normal structure and no tumor or other lesion was detected (Figure 13(c)). CD3+, CD4+, and CD8+ T cells and the ratio of CD4+/CD8+ T cells from peripheral blood samples of different groups were analyzed by flow cytometer. There was no significant change in the subpopulation of T cells among different groups when using the Friedman M test (Figure 13(d)).

## 4. Discussion

In the present study, we showed that tSKPs could be isolated from FBs initially expanded in monolayer culture and presented similar properties of self-renew and differentiation potentials as traditional SKPs. These tSKPs expressed classic SKP markers of Versican, Fibronectin, Vimentin, Sox2, and Nestin and displayed the differentiation capacity for adipocytes, osteocytes, SMCs, and Schwann cells even though the multipotency was reduced in tSKPs from FBs at high (P10) passage. Moreover, tSKPs generated from an adherent monolayer culture system were heterogeneous consisting of both proliferative and aggregated cells. To determine whether the spheres were merely the clusters of aggregated FBs, we compared cell cycle, specific marker expression, differentiation potentials, and transcriptional profiles of tSKPs with FBs. We found that FBs were distinctive from tSKPs in spite of sharing several characteristics in certain properties. To further assess the biosafety of tSKPs and FBs, we adopted karyotyping, major histocompatibility complex analysis, transplant of cells, anatomic pathology, and analysis of subset of T cells, confirming that tSKPs as well as previous FBs were poorly tumorigenic. Together, these findings supported that tSKPs represented as a candidate for regenerative medicine.

The human skin is an adequate source containing various stem cells, making it a good candidate for research and transplantation. Toma et al. reported to isolate hSKPs from neonatal human foreskin tissue in 2005 [24]. Like their rodent counterparts, hSKPs expressed Nestin, Fibronectin, and Vimentin and differentiated to both neural and mesodermal cell types. Although rodent SKPs have been cultured successfully with large quantity, hSKPs are still difficult to culture. The present procedure for generating hSKPs was tedious and time-consuming with only a few thousands of hSKPs available [4]. In 2004, Joannides et al. pioneered the growth of hSKPs in attachment conditions supplemented with serum [9], the protocol of which was basically reproduced in the case with cheek skin by Yoshikawa et al. in 2013 [10]. In 2012, Wenzel et al. and Hill et al. tried to generate suspension SKP-like spheres from monolayer dermal cultures, then confirmed that these spheres were morphologically and functionally similar to traditionally isolated hSKPs [11, 12]. When culturing cells by methods mentioned above, we found the suspension spheres tended to adhere to the plastic while premature adherence would result in a reduced yield of hSKPs [19]. We therefore optimized procedures by treating the flasks with poly-HEMA, a biomaterial that could be used as nonadherent coating materials [20]. The method demonstrated in our study successfully generated cytospheres, and these spheres expressed characteristic SKP markers of Versican, Fibronectin, Vimentin, Sox2, and Nestin. As for multipotent differentiation potentials, we showed tSKPs could be, like previously described SKPs [24], differentiated to both mesodermal and neural lineages of adipocytes, osteocytes, SMCs, and Schwann cells. As Wenzel et al. have described, we also found tSKPs isolated from FBs at high passage presented reduced sphere-forming ability and multipotency. This was consistent with the fact that highly passaged FBs lost their perivascular cells, and SKPs derived from foreskin had been reported in that precise location [26]. One limitation of our study was that only one S100*β*-positive cell was detected. Therefore, the results of Schwann cell differentiation should be interpreted with more caution. By contrast, we had not detected neurons in immunofluorescence, though qRT-PCR results showed *β*III-tubulin was significantly increased at the mRNA level after induction. Potential explanations for this finding included the following: (i) The time of induction was insufficient that mRNA was unable to translate corresponding protein timely; (ii) Several studies reported the stemness of tSKPs varied with age and anatomical location of samples [10, 27]. hSKPs from younger subjects had a better differentiation potential than those from elder ones [27], and hSKPs from facial skin exhibited a higher differentiation capacity than those from the trunk or extremity [10]. In light of our samples were from adult foreskins, the reason for unsuccessfully directing into neuron might come from samples themselves rather than our adherent monolayer culture system. As no convincing derivation of neuronal lineage was detected in current article, future studies remain to investigate it.

NCSCs could be cultured *in vitro* by two different forms of suspending neurospheres and adherent monolayer cultures [28, 29]. Since hSKPs were initially known for similar properties as NCSCs, it was unsurprising that tSKPs could be generated from primary adherent culture. However, Jessberger et al. reported that both suspension and adherent NCSCs were heterogeneous population containing fusion and proliferative cells [16]. Moreover, Reynolds and Rietze believed that the majority of spheres were in fact derived from nonstem cells, which indicated that neurospheres could merge with other cells during the formation process [30]. How about tSKPs—a kind of stem cells similar to NCSCs? Interestingly, we found that tSKP spheres formed at faster rate when compared with their traditional counterparts. Therefore, we doubted that cell aggregates might contribute to spheroid formation and accelerate the formation process. To address this question, we determined the nature of endogenous cells through generating tSKPs by EGFP- or mCherry-expressing FBs. If the spheres were from cell proliferation exclusively, only one fluorescent expression would be detected. However, our study found that both EGFP- and mCherry-positive cells appeared in cultures of tSKPs at different densities except for ultralow density of 1 × 10^3^ cells/mL. This finding suggested that cell aggregation involved in sphere formation. A further analysis on proliferative capacity within tSKPs revealed that these spheres also contained proliferative cells. The proliferative cells within tSKPs varied with cell density: the lower the cell density, the larger their percentage of proliferative cells. Regarding the proliferation data shown in our figures, it would be important for us to explore the possible mechanism underlying this behavior. As demonstrated in a recent study by Liu et al., proliferation of hSKPs was regulated by Akt-FOXO3- p27^INK4b^/p15^KIP1^ signaling [31]. Therefore, we are supposed to look at senescence markers described by Liu et al. in tSKPs with different densities in the future study. We herein indicated the heterogeneous property of tSKPs rather than the clonal expansion of a single cell: tSKPs contained both aggregated and proliferative cells.

Since FBs were also known for self-renewal ability [14, 32], we proposed the possibility that the cytospheres were simply clusters of aggregated FBs rather than tSKPs. A hypothesis was that, FB clusters might hold the similar characteristics as tSKPs. To address it, we employed tests to characterize two cells in cell cycle, immunocytochemistry, CD antigen expression profile, capacity to generate adipocytes, osteocytes, SMCs, Schwann cells, and neurons. Despite sharing certain similarities, we achieved two types of cells with distinctive characteristics. Stem cells are known to proliferate at a slow rate as the slow-cycling property is beneficial for appropriate responses to external signals [33, 34]. In our study, tSKPs maintained relatively slow growth rate with most cells in the G1 phase; however, the proliferation rate of FBs was relatively greater with cells equally distributed in the G1 and S+G2 phase. In terms of cell marker expression, tSKPs and FBs differed in the expression of Collagen I, Versican, and Sox2. In principal, Collagen I mostly occurs in terminally differentiated cells of the skin, bone, heart, and others, while being out of expression in undifferentiated multipotent precursors [35]. The negative expression of Collagen I in tSKPs eliminated their fibroblast origin. Conversely, FBs were positive for Collagen I, which indicate their difference in nature. Versican and Sox2 were traditional surface markers of SKPs as reported [24, 36]. Versican is a large extracellular matrix proteoglycan that participates in cell adhesion, migration, and proliferation and is often considered as an antiadhesion molecule [37]. Sox2 is a transcription factor that maintains the pluripotency and self-renewal properties of undifferentiated embryonic stem cells, which also plays a critical role in the maintenance of embryonic and neural stem cells [38]. Johnston et al. reported that the skin repair property held by nerve-derived neural crest precursors was depended on Sox2 [39]. Moreover, the neurogenic property of hSKPs was restricted to the p75NTR^+^CD56^+^ subgroup and mediated by Sox2 expression levels [40]. The positive expression of Versican and Sox2 suggested tSKPs' similar property as traditional SKPs and indicated a stronger differentiation potential compared with FBs. The latter consideration was verified by our induction results that FBs were lost of neurogenic property. As for the CD antigen expression, both tSKPs and FBs expressed CD90, CD105, CD73, and CD44, while lacking expression of the negative cocktail of CD19, CD45, CD11b, CD34, and HLA-DR. The expression level of C105 was significantly different in two groups, which might be regarded as a further identified marker.

The difference in growth pattern, protein expression, and differentiated potentials indicated an underlying difference in transcriptomes. Through comparing the transcriptional profiles of tSKPs and FBs, we acquired a total of 1,260 DEGs. GO analysis of the DEGs showed that these DEGs were mostly enriched in protein binding, extracellular region, multipeptidase activity, growth, and immune regulation, revealing complex mechanisms involved in the transculture process. KEGG analysis revealed two major developmental signaling pathways of focal adhesion and TNF signal pathway. The focal adhesion signal pathway plays essential roles in important biological processes including cell motility, cell proliferation, and cell differentiation [41]. In the focal adhesion signal pathway, tSKPs *vs*. FBs upregulated 18 DEGs and downregulated 36 DEGs, indicating that the altered condition from adherent FBs to suspension tSKPs could suppress cellular adhesion. TNF is a protein superfamily participating in a multibiological process, including regulation of immune response, inflammation, proliferation, differentiation, apoptosis, and embryogenesis [42]. In this pathway, tSKPs vs. FBs upregulated 25 DEGs and downregulated 5 DEGs, indicating that tSKPs held a superior immunoregulatory function. In general, immunomodulatory function is one of the key characteristics of stem cells [43]. It was reported that SKPs could modulate the immune response through cell contact and secretion of soluble inhibitory factors [18]. Regulation at a transcriptional level is essential for gene expression. TFs regulate gene expression by binding to a specific upstream nucleotide sequence to achieve biological functions. Analysis of TFs varied between tSKPs and FBs is needed to accomplish an insight of potential mechanisms involved. The expression of Foxc1 was reported as correlated with hair cycle in hair follicle stem cells [44]. Bach2 mediated inflammatory response and could promote B cell development [45]. The Nr4a2 expression was associated with neuron progenitors [46] and could regulate neurogenesis of hippocampus neural stem cells [47]. The Klf family contributed to self-renewal and cellular reprogramming of stem cells [48]. The upregulation of these TFs in tSKPs might be involved in the neural-phenotype of tSKPs, whereas further functional studies were needed to explore it in detail.

One of the major questions raised by our findings was the nature of the cells that contributed to tSKP spheres. Although we had confirmed that tSKPs and FBs were distinct, there was a substantial possibility that FBs might merge into tSKP spheres. In light of the aforementioned problem, we investigated the biological safety of both tSKPs and initial FBs to ensure that the potential mixed FBs would not affect sphere clinical application, although it is unlikely that a clinical-grade tSKP-product may be contaminated with fibroblasts. Abnormal karyotypes are closely associated with tumorigenesis [49]. Our work here showed that both tSKPs and FBs could maintain their normal karyotypes when cultured *ex vivo* in long term. Clinical use of tSKPs required these cells to be poorly to no immunogenic, and we thus determined their immunological profiles. We showed here for the first time that tSKPs, generated from adherent FBs, expressed HLA-I while lacked expression of HLA-DR. Moreover, the immunophenotype of FBs was similar to tSKPs. Due to aberrant HLA-DR expression, cells did not possess antigen-presenting functions and could not activate lymphocytes. Therefore, we demonstrated that both tSKPs and FBs exhibited low immunogenicity. A further *ex vivo* experiment showed transplantation of tSKPs or FBs did not affect the body weight and body temperature of SCID mice. There was no tumor formations observed and no significant difference found in subset analysis of T lymphocytes among each group.

## 5. Conclusions

In summary, we have demonstrated here that tSKPs could be isolated from an adherent monolayer culture system, and these spheres were morphologically and functionally similar to traditional SKPs. The formed spheres were heterogeneous populations consisting of both aggregated and proliferative cells, and the rate of proliferative cells within spheres correlated with a seeding density; the lower the cell density, the larger the percentage of proliferative cells. Although isolated from monolayer adherent FBs, tSKPs were distinctive from FBs despite the two sharing similarity in certain properties. We also showed that both tSKPs and FBs could be considered as nontumorigenic *ex vivo* and *in vivo*, indicating the potential mixture of FBs in spheres did not affect the biosafety of tSKPs. Taken together, we suggested tSKPs had potential therapeutic applications in cell therapy and regenerative medicine.

However, our study was a preliminary study on biosafety assessment. More research should be made on investigating the costimulatory molecule expression, the immunophenotype in inflammatory environment, and the immunosuppressive features both *in vitro* and *in vivo*. In addition, it is now believed that stem cells with neurogenic capacities ascribed to dedifferentiated Schwann cells presenting in spheroid culture [40, 50, 51], although it is still unclear whether the diversity will affect the biotechnological applications of hSKPs or not. In the current study, we are limited in our experimental ability to address these issues on tSKPs. In the future, related markers involved in distinguishing purified tSKPs from other cells will be carefully examined to clarify these points.

## Figures and Tables

**Table 1:** Summary of mapping results (mapping to reference genes).

Sample name	Total reads	Total mapped	Multiple mapped	Uniquely mapped	Nonsplice reads	Splice reads
tSKPs_RNA	10730944	10389834 (96.82%)	600661 (5.6%)	9789173 (91.22%)	7759368 (72.31%)	2029805 (18.92%)
FBs_RNA	12079337	11819139 (97.85%)	586464 (4.86%)	11232675 (92.99%)	9050555 (74.93%)	2182120 (18.06%)

**Table 2:** List of major transcription factors with strong evidence involved in the transculturing process (∣log_2_(Fold change)∣ > 1).

Function	Upregulated transcription factor	Downregulated transcription factor
DNA binding	Snail2, Pou2f2, Foxc1, Nfkb2, Bach2, Foxo3, Nr1d1, Creg1, Foxo1, Nr4a2, Klf10, Tg1f1	Meox2, Foxm1, Klf7, Meis2, Mitf
Transactivation	Foxc1, Rora, Foxo3, Foxo1, Nr4a2, Tg1f1	Meox2, Klf7, Meis2, Aff3, Cited2, Mitf
TF PPI	Nfkbia, Bag1	Bag1, Fhl2, Hmgb2, Cited2
Coactivation	Ncoa7, Nr4a2	Cited2
Other	/	Id1

## Data Availability

The data used to support the findings of this study are available from the corresponding author upon request.

## Abbreviations

SKPs: Skin-derived precursors
bFGF: Basic fibroblast growth factor
EGF: Epidermal growth factor
hSKPs: Human SKPs
NCSCs: Neural crest stem cells
tSKPs: Transcultured hSKPs
FBs: Fibroblasts
RNA-seq: RNA-sequencing
PBS: Phosphate buffer solution
PFA: Paraformaldehyde
BSA: Bull serum albumin
*α*-SMA: Anti-*α*-smooth muscle actin
SMC: Smooth muscle cell
EGFP: Enhanced green fluorescent protein
SCID: Severe combined immune-deficient
DEGs: Differentially expressed genes
GO: Gene ontology
KEGG: Kyoto encyclopedia of genes and genomes
SD: Standard deviation
Poly-HEMA: Poly-2-hydroxyethyl methacrylate
PPAR-*γ*: Peroxisome proliferator-activated receptor-*γ*
FABP-4: Fatty acid binding protein-4
GFAP: Glial fibrillary acid protein.

## Supplementary Materials

Supplementary Materials Fig. S1: tSKPs produced from P10 FBs. (A) Immunocytochemical analysis of tSKPs from P10 FBs. tSKPs expressed Versican (red), Fibronectin (red), Vimentin (red), Sox2 (red), and Nestin (red), while were negative for Collagen I (red). Nuclei of all the cells were counterstained with DAPI (blue). Scale bars: 100 *μ*m. (B) Multiple differentiation potentials of tSKPs generated from P10 FBs. tSKPs can be directed into adipocytes, osteocytes, and SMCs, while cannot be induced into Schwann cells and neurons. Fig. S2: transduction of FBs with retroviruses expressing EGFP or mCherry. (A) Phase and fluorescence photomicrograph of FBs transfected with retroviruses expressing EGFP. (B) Phase and fluorescence photomicrograph of EGFP-transduced FBs after passage. (C) Phase and fluorescence photomicrograph of FBs transfected with retroviruses expressing mCherry. (D) Phase and fluorescence photomicrograph of mCherry-transduced FBs after passage. Scale bars: 100 *μ*m. Fig. S3: tSKP spheres contain BrdU-positive (green) cells. Phase contrast image of tSKPs (left panel). Immunofluorescence staining showed part of cells within tSKP spheres were positive for BrdU, and nuclei of all the cells were counterstained with DAPI (blue) (right panel). Scale bars: 100 *μ*m. Fig. S4: EGFP-labelled tSKPs and FBs can be monitored in the dermis and s.c. tissue at 2 weeks after transplantation. The fluorescence intensity in the dermis and s.c. layers of (A) tSKPs and (B) FBs. Fig. S5: effect of FBs and tSKPs to SCID mice on gross. Table S1: media. Table S2: primer sequences for qRT-PCR. Table S3: full lists of upregulated and downregulated DEGs between tSKPs and FBs. Table S4: experiment treatments.
