# Supplementary material for: Isolation, Characterization, and Safety Evaluation of Human Skin-Derived Precursors from an Adherent Monolayer Culture System
Source: Stem Cells Int. 2019 Aug 19;2019:9194560. doi: 10.1155/2019/9194560 (PMC6721512; doi:10.1155/2019/9194560)
Supplement: Supplementary Materials — Fig. S1: tSKPs produced from P10 FBs. (A) Immunocytochemical analysis of tSKPs from P10 FBs. tSKPs expressed Versican (red), Fibronectin (red), Vimentin (red), Sox2 (red), and Nestin (red), while were negative for Collagen I (red). Nuclei of all the cells were counterstained with DAPI (blue). Scale bars: 100 μm. (B) Multiple differentiation potentials of tSKPs generated from P10 FBs. tSKPs can be directed into adipocytes, osteocytes, and SMCs, while cannot be induced into Schwann cells and neurons. Fig. S2: transduction of FBs with retroviruses expressing EGFP or mCherry. (A) Phase and fluorescence photomicrograph of FBs transfected with retroviruses expressing EGFP. (B) Phase and fluorescence photomicrograph of EGFP-transduced FBs after passage. (C) Phase and fluorescence photomicrograph of FBs transfected with retroviruses expressing mCherry. (D) Phase and fluorescence photomicrograph of mCherry-transduced FBs after passage. Scale bars: 100 μm. Fig. S3: tSKP spheres contain BrdU-positive (green) cells. Phase contrast image of tSKPs (left panel). Immunofluorescence staining showed part of cells within tSKP spheres were positive for BrdU, and nuclei of all the cells were counterstained with DAPI (blue) (right panel). Scale bars: 100 μm. Fig. S4: EGFP-labelled tSKPs and FBs can be monitored in the dermis and s.c. tissue at 2 weeks after transplantation. The fluorescence intensity in the dermis and s.c. layers of (A) tSKPs and (B) FBs. Fig. S5: effect of FBs and tSKPs to SCID mice on gross. Table S1: media. Table S2: primer sequences for qRT-PCR. Table S3: full lists of upregulated and downregulated DEGs between tSKPs and FBs. Table S4: experiment treatments. [file 9194560.f1.docx]

**Supporting information**

**
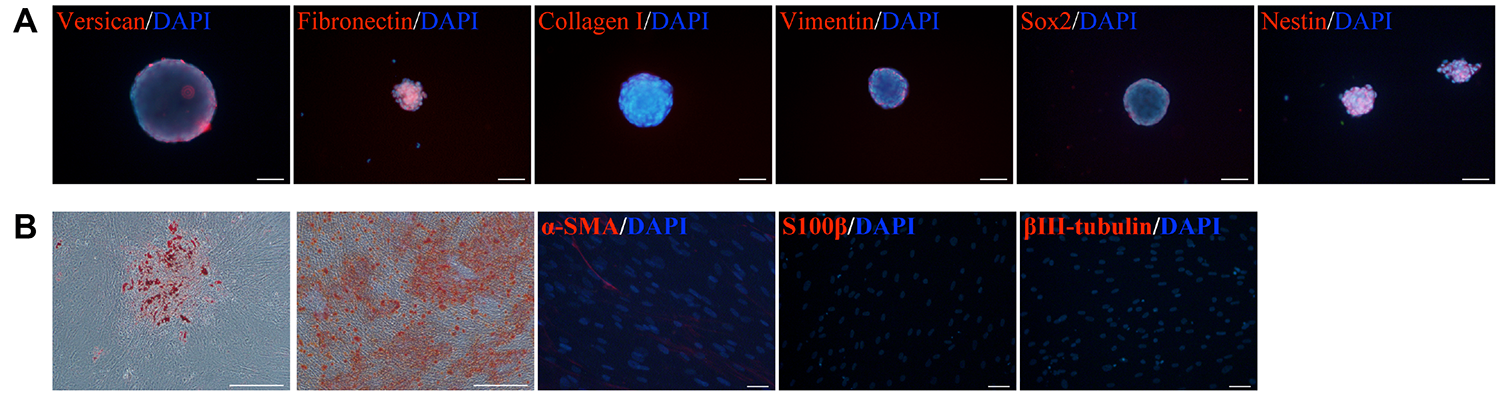
**

**Fig. S1. tSKPs produced from P10 FBs.**

(A): Immunocytochemical analysis of tSKPs from P10 FBs. tSKPs expressed Versican (red), Fibronectin (red), Vimentin (red), Sox2 (red) and Nestin (red), while were negative for Collagen I (red). Nuclei of all the cells were counterstained with DAPI (blue). Scale bars: 100 μm.

(B): Multiple differentiation potentials of tSKPs generated from P10 FBs. tSKPs can be directed into adipocytes, osteocytes and SMCs, while cannot be induced into Schwann cells and neurons.

**
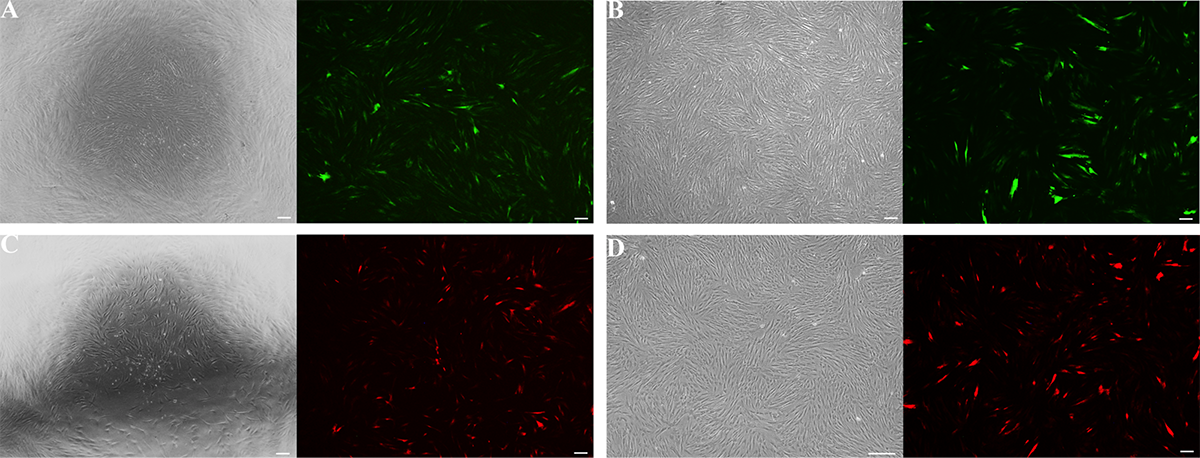
**

**Fig. S2. Transduction of FBs with retroviruses expressing EGFP or mCherry.**

1. Phase and fluorescence photomicrograph of FBs transfected with retroviruses expressing EGFP.
2. Phase and fluorescence photomicrograph of EGFP-transduced FBs after passage.
3. Phase and fluorescence photomicrograph of FBs transfected with retroviruses expressing mCherry.
4. Phase and fluorescence photomicrograph of mCherry-transduced FBs after passage. Scale bars: 100 μm.


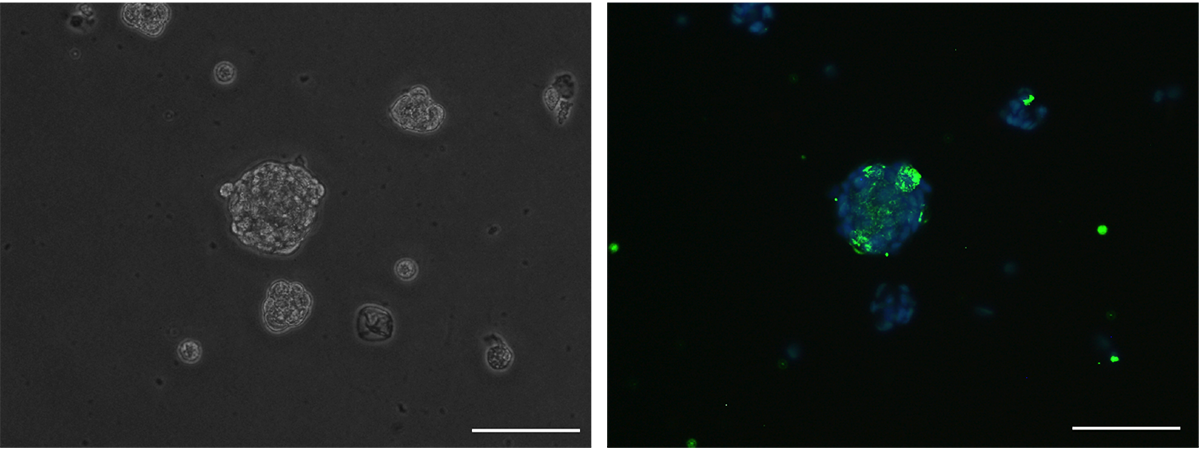


**Fig. S3. tSKPs spheres contain BrdU-positive (green) cells.** Phase contrast image of tSKPs (left panel). Immunoflurescence staining showed part of cells within tSKPs spheres were positive for BrdU, and nuclei of all the cells were counterstained with DAPI (blue) (right panel). Scale bars: 100 μm.


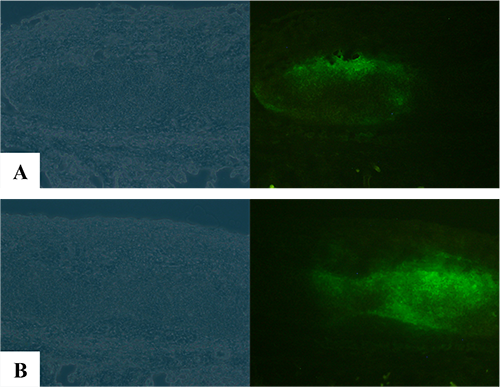


**Fig. S4. EGFP-labelled tSKPs and FBs can be monitored in the dermis and s.c tissue at 2 weeks after transplantation**. The fluorescence intensity in the dermis and s.c. layers of (A) tSKPs and (B) FBs.


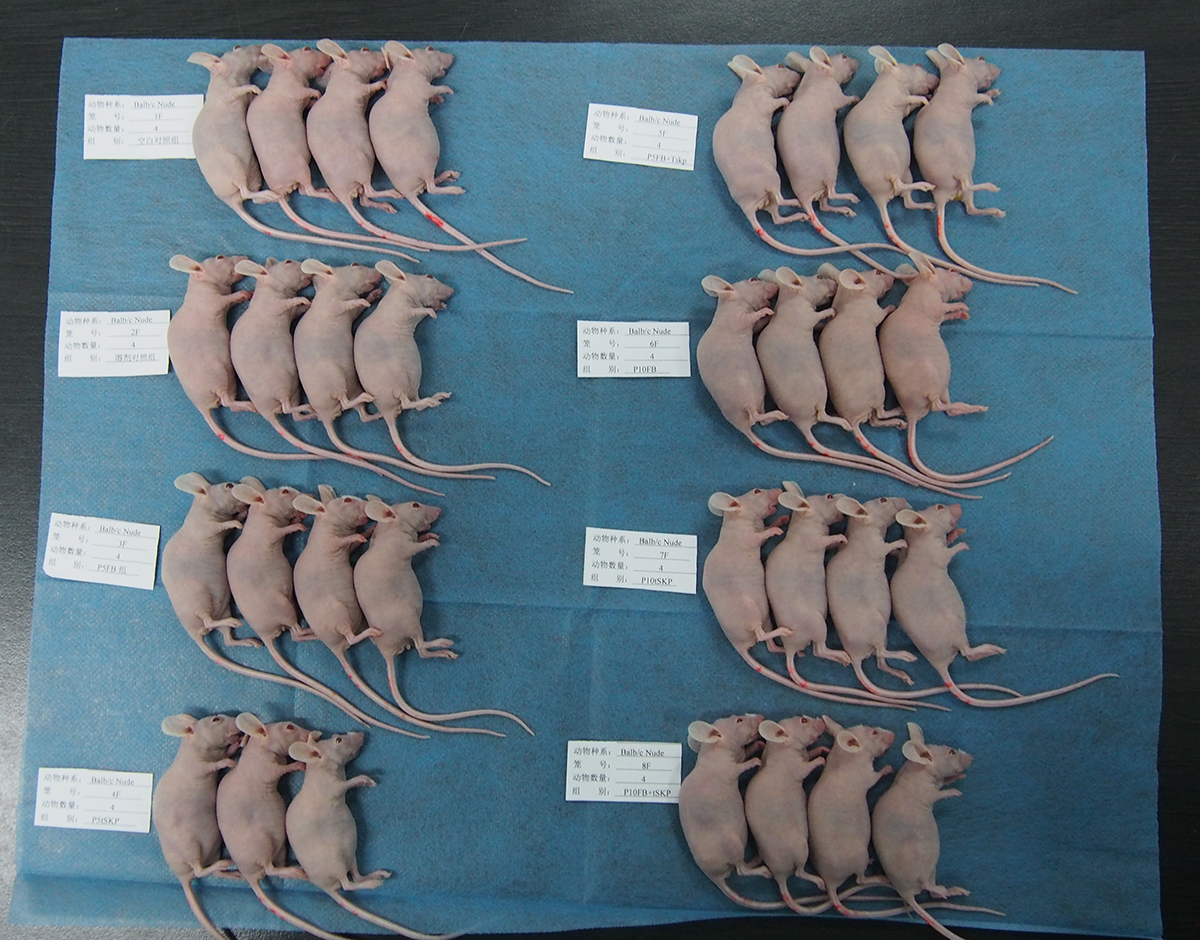


**Fig. S5. Effect of FBs and tSKPs to SCID mice on gross.**

**Table S1. Media**

| Medium | Reagent (Supplier) |
| --- | --- |
| FB adherent medium | DMEM/F12 (Hyclone, USA) 3:1 + 10% FBS (Clark, Australia) + 1% P/S (Invitrogen, USA) |
| SKP proliferating medium | DMEM/F12 (Hyclone, USA) 3:1 + 20 ng/ml EGF (Millipore, USA) + 40 ng/ml bFGF (Millipore, USA) + 2% B27 (Gibco, USA) + 1% P/S (Invitrogen, USA) |
| SKP adherent medium | DMEM/F12 (Hyclone, USA) 3:1 + 20 ng/ml EGF (Millipore, USA) + 40 ng/ml bFGF (Millipore, USA) + 2% B27 (GIBCO, USA) + 5% FBS (Clark, Australia) + 1% P/S (Invitrogen, USA) |
| SMC differentiation medium | DMEM/F12 (Hyclone, USA) 3:1 + 5% FBS (Clark, Australia) + 2.5 ng/ml TGF-β1 (Pepro Tech, USA) + 5 ng/ml PDGF (Pepro Tech, USA) + 1% P/S (Invitrogen, USA) |
| Schwann cell differentiation medium | DMEM/F12 (Hyclone, USA) 3:1 + 2% N_2_ (Invitrogen, USA) + 5 μmol/l forskolin (Sigma, USA) + 50 ng/ml heregulin-1β (Pepro Tech, USA) + 1% FBS (Clark, Australia) |
| Neuron differentiation medium | DMEM/F12 (Hyclone, USA) 3:1 + 10 ng/ml NT-3 (Pepro Tech, USA) + 50 ng/ml NGF (Pepro Tech, USA) + 50 ng/ml BDNF (Pepro Tech, USA) + 1% FBS (Clark, Australia) |

FB, fibroblast; DMEM, Dulbecco’s modified Eagle medium; FBS, fetal bovine serum; P/S, penicillin/streptomycin; SKP, skin-derived precursor; EGF, epidermal growth factor; bFGF, basic fibroblast growth factor; SMC, smooth muscle cell; TGF, transforming growth factor; PDGF, platelet-derived growth factor; NGF, nerve growth factor; BDNF, brain-derived neutrophic factor.

**Table S2. Primer sequences for qRT-PCR.**

| Target gene | Direction | Primer sequences |
| --- | --- | --- |
| β-actin | Forward | CATGTACGTTGCTATCCAGGC |
|  | Reverse | CTCCTTAATGTCACGCACGAT |
| PPAR-γ | Forward | CCGCCCAGGTTTGCTGAATGTGAAG |
|  | Reverse | AATGTTGGCAGTGGCTCAGGACTC |
| FABP-4 | Forward | TGATAAACTGGTGGTGGAATGCGTCA |
|  | Reverse | TCAATGCGAACTTCAGTCCAGGTCAA |
| Rnux2 | Forward | GCCACCACTCACTACCACACCTACCT |
|  | Reverse | GGCTTCCATCAGCGTCAACACCATCA |
| α-SMA | Forward | GGCATCATCACCAACTGGGACGACA |
|  | Reverse | AGCACCGCCTGGATAGCCACATACA |
| S100β | Forward | CTGGAAGGGAGGGAGACAAGCACAAG |
|  | Reverse | AGAACTCGTGGCAGGCAGTAGTAACC |
| GFAP | Forward | ATCCGCACGCAGTATGAGGCAATGG |
|  | Reverse | AGAGACTCCAGGTCGCAGGTCAAGG |
| βIII-tubulin | Forward | TGGATGTGGTGCGGAAGGAGTGTGAA |
|  | Reverse | GTGGATGGACAGCGTGGCGTTGTAG |
| Foxc1 | Forward | GCTACATCGCTCTTATCACCA |
|  | Reverse | CCCTGCTTATTGTCCCGATAG |
| Bach2 | Forward | TCCTTGCCACAGAACATCAGGAAC |
|  | Reverse | TGGATGTCTCGGCAAACTTCCTGG |
| Nr4a2 | Forward | AGAGACGCGGAGAACTCCTA |
|  | Reverse | AGGCATGGCTTCAGCCGAGT |
| Klf10 | Forward | GTGACCGTCGGTTTATGAGG |
|  | Reverse | ACTTCCATTTGCCAGTTTGG |
| Foxm1 | Forward | ATACGTGGATTGAGGACCACT |
|  | Reverse | TCCAATGTCAAGTAGCGGTTG |
| Meox2 | Forward | TCCTGTGCTCCAACTCTTC |
|  | Reverse | CTTCTCAACCTCGTGTCCT |
| Aff3 | Forward | ACTCAACAGGATGATGGCAC |
|  | Reverse | TGCCTAAAGTGTTCTGGATC |
| Cited2 | Forward | ACCATCACCCTGCCCACC |
|  | Reverse | CGTAGTATGTGCTCGCCCA |

**Table S3. Full lists of up-regulated and down-regulated DEGs between tSKPs and FBs.**

| Gene name | Type | Genetype | Description |
| --- | --- | --- | --- |
| NIPAL3 | Down-regulated | protein_coding | NIPA-like domain containing 3 [Source:HGNC Symbol;Acc:25233] |
| LASP1 | Down-regulated | protein_coding | LIM and SH3 protein 1 [Source:HGNC Symbol;Acc:6513] |
| CYP26B1 | Up-regulated | protein_coding | heat shock protein, alpha-crystallin-related, B6 [Source:HGNC Symbol;Acc:26511] |
| SLC7A2 | Up-regulated | protein_coding | coatomer protein complex, subunit zeta 2 [Source:HGNC Symbol;Acc:19356] |
| FKBP4 | Up-regulated | protein_coding | integrin, alpha 3 (antigen CD49C, alpha 3 subunit of VLA-3 receptor) [Source:HGNC Symbol;Acc:6139] |
| CAMKK1 | Up-regulated | protein_coding | aldehyde dehydrogenase 3 family, member B1 [Source:HGNC Symbol;Acc:410] |
| HSPB6 | Down-regulated | protein_coding | cytohesin 3 [Source:HGNC Symbol;Acc:9504] |
| PDK4 | Up-regulated | protein_coding | nuclear factor I/X (CCAAT-binding transcription factor) [Source:HGNC Symbol;Acc:7788] |
| THSD7A | Up-regulated | protein_coding | anillin, actin binding protein [Source:HGNC Symbol;Acc:14082] |
| COPZ2 | Down-regulated | protein_coding | RAB GTPase activating protein 1 [Source:HGNC Symbol;Acc:17155] |
| ITGA3 | Down-regulated | protein_coding | sema domain, immunoglobulin domain (Ig), short basic domain, secreted, (semaphorin) 3B [Source:HGNC Symbol;Acc:10724] |
| TMEM132A | Up-regulated | protein_coding | EH-domain containing 3 [Source:HGNC Symbol;Acc:3244] |
| CACNA1G | Up-regulated | protein_coding | claudin 11 [Source:HGNC Symbol;Acc:8514] |
| JHDM1D | Up-regulated | protein_coding | G protein-coupled receptor, family C, group 5, member A [Source:HGNC Symbol;Acc:9836] |
| ETV1 | Up-regulated | protein_coding | transforming, acidic coiled-coil containing protein 3 [Source:HGNC Symbol;Acc:11524] |
| ALDH3B1 | Down-regulated | protein_coding | ralA binding protein 1 [Source:HGNC Symbol;Acc:9841] |
| IFRD1 | Up-regulated | protein_coding | four and a half LIM domains 1 [Source:HGNC Symbol;Acc:3702] |
| GAS7 | Up-regulated | protein_coding | EH-domain containing 2 [Source:HGNC Symbol;Acc:3243] |
| JARID2 | Up-regulated | protein_coding | vimentin [Source:HGNC Symbol;Acc:12692] |
| CYTH3 | Down-regulated | protein_coding | ubiquitin-like with PHD and ring finger domains 1 [Source:HGNC Symbol;Acc:12556] |
| MGST1 | Up-regulated | protein_coding | vinculin [Source:HGNC Symbol;Acc:12665] |
| NFIX | Down-regulated | protein_coding | death-associated protein kinase 2 [Source:HGNC Symbol;Acc:2675] |
| ST3GAL1 | Up-regulated | protein_coding | capping protein (actin filament), gelsolin-like [Source:HGNC Symbol;Acc:1474] |
| FYN | Up-regulated | protein_coding | phosphatidylinositol-3,4,5-trisphosphate-dependent Rac exchange factor 2 [Source:HGNC Symbol;Acc:22950] |
| HIVEP2 | Up-regulated | protein_coding | KIT ligand [Source:HGNC Symbol;Acc:6343] |
| TSPAN9 | Up-regulated | protein_coding | steroid-5-alpha-reductase, alpha polypeptide 2 (3-oxo-5 alpha-steroid delta 4-dehydrogenase alpha 2) [Source:HGNC Symbol;Acc:11285] |
| SYT7 | Up-regulated | protein_coding | elastin [Source:HGNC Symbol;Acc:3327] |
| PLAUR | Up-regulated | protein_coding | dickkopf 3 homolog (Xenopus laevis) [Source:HGNC Symbol;Acc:2893] |
| ANLN | Down-regulated | protein_coding | LIM domain and actin binding 1 [Source:HGNC Symbol;Acc:24636] |
| RABGAP1 | Down-regulated | protein_coding | spectrin repeat containing, nuclear envelope 2 [Source:HGNC Symbol;Acc:17084] |
| DCN | Up-regulated | protein_coding | coiled-coil domain containing 85A [Source:HGNC Symbol;Acc:29400] |
| SEMA3B | Down-regulated | processed_transcript | protein phosphatase 1, regulatory subunit 12A [Source:HGNC Symbol;Acc:7618] |
| EHD3 | Down-regulated | protein_coding | ATPase, Ca++ transporting, plasma membrane 4 [Source:HGNC Symbol;Acc:817] |
| CLDN11 | Down-regulated | protein_coding | collagen, type XI, alpha 1 [Source:HGNC Symbol;Acc:2186] |
| GPRC5A | Down-regulated | protein_coding | aryl-hydrocarbon receptor repressor [Source:HGNC Symbol;Acc:346] |
| TACC3 | Down-regulated | protein_coding | LIM and calponin homology domains 1 [Source:HGNC Symbol;Acc:29191] |
| BID | Up-regulated | protein_coding | calponin 2 [Source:HGNC Symbol;Acc:2156] |
| RALBP1 | Down-regulated | protein_coding | solute carrier family 9, subfamily A (NHE3, cation proton antiporter 3), member 3 regulator 2 [Source:HGNC Symbol;Acc:11076] |
| WWTR1 | Up-regulated | protein_coding | importin 5 [Source:HGNC Symbol;Acc:6402] |
| SNAI2 | Up-regulated | protein_coding | myosin light chain kinase [Source:HGNC Symbol;Acc:7590] |
| HGF | Up-regulated | protein_coding | asp (abnormal spindle) homolog, microcephaly associated (Drosophila) [Source:HGNC Symbol;Acc:19048] |
| FHL1 | Down-regulated | protein_coding | neuron navigator 3 [Source:HGNC Symbol;Acc:15998] |
| EHD2 | Down-regulated | protein_coding | tetratricopeptide repeat domain 7A [Source:HGNC Symbol;Acc:19750] |
| TYMP | Up-regulated | protein_coding | follistatin-like 3 (secreted glycoprotein) [Source:HGNC Symbol;Acc:3973] |
| VIM | Down-regulated | protein_coding | protein tyrosine phosphatase, non-receptor type 21 [Source:HGNC Symbol;Acc:9651] |
| FAS | Up-regulated | protein_coding | WD repeat domain 1 [Source:HGNC Symbol;Acc:12754] |
| AGPAT4 | Up-regulated | protein_coding | ribosomal protein S6 kinase, 90kDa, polypeptide 2 [Source:HGNC Symbol;Acc:10431] |
| IFNGR1 | Up-regulated | protein_coding | LIM and cysteine-rich domains 1 [Source:HGNC Symbol;Acc:6633] |
| POU2F2 | Up-regulated | protein_coding | thyroid hormone receptor interactor 13 [Source:HGNC Symbol;Acc:12307] |
| UHRF1 | Down-regulated | processed_transcript | actinin, alpha 1 [Source:HGNC Symbol;Acc:163] |
| VCL | Down-regulated | protein_coding | LIM and senescent cell antigen-like domains 2 [Source:HGNC Symbol;Acc:16084] |
| DAPK2 | Down-regulated | protein_coding | SPEG complex locus [Source:HGNC Symbol;Acc:16901] |
| RAB27B | Up-regulated | protein_coding | thyrotropin-releasing hormone degrading enzyme [Source:HGNC Symbol;Acc:30748] |
| TNC | Up-regulated | protein_coding | murine retrovirus integration site 1 homolog [Source:HGNC Symbol;Acc:7237] |
| FAM65C | Up-regulated | protein_coding | fermitin family member 2 [Source:HGNC Symbol;Acc:15767] |
| CAPG | Down-regulated | protein_coding | furry homolog (Drosophila) [Source:HGNC Symbol;Acc:20367] |
| GPM6B | Up-regulated | protein_coding | monoglyceride lipase [Source:HGNC Symbol;Acc:17038] |
| PREX2 | Down-regulated | protein_coding | netrin 4 [Source:HGNC Symbol;Acc:13658] |
| ARHGAP6 | Up-regulated | protein_coding | NUAK family, SNF1-like kinase, 1 [Source:HGNC Symbol;Acc:14311] |
| KITLG | Down-regulated | protein_coding | G-2 and S-phase expressed 1 [Source:HGNC Symbol;Acc:13698] |
| SRD5A2 | Down-regulated | processed_transcript | sema domain, immunoglobulin domain (Ig), short basic domain, secreted, (semaphorin) 3C [Source:HGNC Symbol;Acc:10725] |
| LTBP1 | Up-regulated | protein_coding | RAS protein activator like 2 [Source:HGNC Symbol;Acc:9874] |
| ELN | Down-regulated | protein_coding | actin, beta [Source:HGNC Symbol;Acc:132] |
| HEXB | Up-regulated | protein_coding | RNA binding motif, single stranded interacting protein 2 [Source:HGNC Symbol;Acc:9909] |
| DKK3 | Down-regulated | protein_coding | ankyrin repeat domain 13A [Source:HGNC Symbol;Acc:21268] |
| LIMA1 | Down-regulated | protein_coding | protein phosphatase 1, regulatory subunit 12B [Source:HGNC Symbol;Acc:7619] |
| LAMC3 | Up-regulated | protein_coding | PHD finger protein 17 [Source:HGNC Symbol;Acc:30027] |
| PTPRN | Up-regulated | protein_coding | monooxygenase, DBH-like 1 [Source:HGNC Symbol;Acc:21063] |
| FOXC1 | Up-regulated | protein_coding | ATPase, aminophospholipid transporter, class I, type 8B, member 1 [Source:HGNC Symbol;Acc:3706] |
| SYNE2 | Down-regulated | protein_coding | cordon-bleu WH2 repeat protein-like 1 [Source:HGNC Symbol;Acc:23571] |
| CYFIP2 | Up-regulated | protein_coding | potassium channel, subfamily K, member 2 [Source:HGNC Symbol;Acc:6277] |
| CCDC85A | Down-regulated | protein_coding | ADAM metallopeptidase with thrombospondin type 1 motif, 2 [Source:HGNC Symbol;Acc:218] |
| PRDM1 | Up-regulated | protein_coding | aurora kinase A [Source:HGNC Symbol;Acc:11393] |
| LAMC2 | Up-regulated | protein_coding | TPX2, microtubule-associated, homolog (Xenopus laevis) [Source:HGNC Symbol;Acc:1249] |
| PPP1R12A | Down-regulated | protein_coding | paxillin [Source:HGNC Symbol;Acc:9718] |
| ATP2B4 | Down-regulated | protein_coding | baculoviral IAP repeat containing 5 [Source:HGNC Symbol;Acc:593] |
| CDK17 | Up-regulated | protein_coding | poly(rC) binding protein 4 [Source:HGNC Symbol;Acc:8652] |
| SLC2A3 | Up-regulated | protein_coding | integrin, alpha 6 [Source:HGNC Symbol;Acc:6142] |
| PSD | Up-regulated | protein_coding | zinc finger homeobox 4 [Source:HGNC Symbol;Acc:30939] |
| PTPRU | Up-regulated | protein_coding | coiled-coil domain containing 80 [Source:HGNC Symbol;Acc:30649] |
| COL11A1 | Down-regulated | protein_coding | ezrin [Source:HGNC Symbol;Acc:12691] |
| AHRR | Down-regulated | protein_coding | myosin, light chain 6, alkali, smooth muscle and non-muscle [Source:HGNC Symbol;Acc:7587] |
| LIMCH1 | Down-regulated | protein_coding | TBC1 domain family, member 2 [Source:HGNC Symbol;Acc:18026] |
| CNN2 | Down-regulated | protein_coding | desmoplakin [Source:HGNC Symbol;Acc:3052] |
| SLC9A3R2 | Down-regulated | protein_coding | stearoyl-CoA desaturase (delta-9-desaturase) [Source:HGNC Symbol;Acc:10571] |
| IPO5 | Down-regulated | protein_coding | actin binding LIM protein 1 [Source:HGNC Symbol;Acc:78] |
| NTN1 | Up-regulated | protein_coding | paralemmin [Source:HGNC Symbol;Acc:8594] |
| MYLK | Down-regulated | protein_coding | mediator complex subunit 15 [Source:HGNC Symbol;Acc:14248] |
| FAM107B | Up-regulated | protein_coding | MICAL-like 1 [Source:HGNC Symbol;Acc:29804] |
| ASPM | Down-regulated | protein_coding | Sad1 and UNC84 domain containing 2 [Source:HGNC Symbol;Acc:14210] |
| DDX3Y | Up-regulated | protein_coding | minichromosome maintenance complex component 5 [Source:HGNC Symbol;Acc:6948] |
| NAV3 | Down-regulated | protein_coding | myosin, heavy chain 9, non-muscle [Source:HGNC Symbol;Acc:7579] |
| ACSL4 | Up-regulated | protein_coding | Ran GTPase activating protein 1 [Source:HGNC Symbol;Acc:9854] |
| TTC7A | Down-regulated | protein_coding | phosphorylase, glycogen, liver [Source:HGNC Symbol;Acc:9725] |
| GAL | Up-regulated | protein_coding | serine palmitoyltransferase, long chain base subunit 2 [Source:HGNC Symbol;Acc:11278] |
| RORA | Up-regulated | protein_coding | Ras and Rab interactor 3 [Source:HGNC Symbol;Acc:18751] |
| ATP1B3 | Up-regulated | protein_coding | legumain [Source:HGNC Symbol;Acc:9472] |
| MAPK6 | Up-regulated | protein_coding | phosphoenolpyruvate carboxykinase 2 (mitochondrial) [Source:HGNC Symbol;Acc:8725] |
| RAB27A | Up-regulated | protein_coding | v-myb myeloblastosis viral oncogene homolog (avian)-like 2 [Source:HGNC Symbol;Acc:7548] |
| SLC44A1 | Up-regulated | protein_coding | myosin, light chain 9, regulatory [Source:HGNC Symbol;Acc:15754] |
| FSTL3 | Down-regulated | protein_coding | jagged 1 [Source:HGNC Symbol;Acc:6188] |
| WIPI1 | Up-regulated | protein_coding | myosin, light chain 12A, regulatory, non-sarcomeric [Source:HGNC Symbol;Acc:16701] |
| PTPN21 | Down-regulated | protein_coding | matrix-remodelling associated 5 [Source:HGNC Symbol;Acc:7539] |
| ATP2B1 | Up-regulated | protein_coding | plastin 3 [Source:HGNC Symbol;Acc:9091] |
| WDR1 | Down-regulated | protein_coding | early endosome antigen 1 [Source:HGNC Symbol;Acc:3185] |
| RPS6KA2 | Down-regulated | protein_coding | fms-related tyrosine kinase 1 [Source:HGNC Symbol;Acc:3763] |
| LMCD1 | Down-regulated | protein_coding | katanin p60 subunit A-like 1 [Source:HGNC Symbol;Acc:28361] |
| TRIP13 | Down-regulated | protein_coding | host cell factor C1 regulator 1 (XPO1 dependent) [Source:HGNC Symbol;Acc:21198] |
| TRIB2 | Up-regulated | protein_coding | WAP four-disulfide core domain 1 [Source:HGNC Symbol;Acc:15466] |
| SLC6A15 | Up-regulated | protein_coding | coactosin-like 1 (Dictyostelium) [Source:HGNC Symbol;Acc:18304] |
| ACTN1 | Down-regulated | protein_coding | piezo-type mechanosensitive ion channel component 1 [Source:HGNC Symbol;Acc:28993] |
| LIMS2 | Down-regulated | protein_coding | calcineurin-like phosphoesterase domain containing 1 [Source:HGNC Symbol;Acc:25632] |
| SPEG | Down-regulated | protein_coding | EH-domain containing 4 [Source:HGNC Symbol;Acc:3245] |
| TFRC | Up-regulated | protein_coding | zinc finger, DHHC-type containing 2 [Source:HGNC Symbol;Acc:18469] |
| TRHDE | Down-regulated | protein_coding | stathmin-like 2 [Source:HGNC Symbol;Acc:10577] |
| MRVI1 | Down-regulated | protein_coding | SH2 domain containing 4A [Source:HGNC Symbol;Acc:26102] |
| FERMT2 | Down-regulated | protein_coding | Rho guanine nucleotide exchange factor (GEF) 10 [Source:HGNC Symbol;Acc:14103] |
| PTGS2 | Up-regulated | protein_coding | protein phosphatase 1, regulatory subunit 13 like [Source:HGNC Symbol;Acc:18838] |
| FRY | Down-regulated | protein_coding | excision repair cross-complementing rodent repair deficiency, complementation group 2 [Source:HGNC Symbol;Acc:3434] |
| CA12 | Up-regulated | protein_coding | cyclin-dependent kinase 6 [Source:HGNC Symbol;Acc:1777] |
| MGLL | Down-regulated | protein_coding | caveolin 2 [Source:HGNC Symbol;Acc:1528] |
| NTN4 | Down-regulated | protein_coding | caveolin 1, caveolae protein, 22kDa [Source:HGNC Symbol;Acc:1527] |
| NUAK1 | Down-regulated | protein_coding | met proto-oncogene (hepatocyte growth factor receptor) [Source:HGNC Symbol;Acc:7029] |
| GTSE1 | Down-regulated | protein_coding | wingless-type MMTV integration site family member 2 [Source:HGNC Symbol;Acc:12780] |
| SEMA3C | Down-regulated | protein_coding | homeobox A13 [Source:HGNC Symbol;Acc:5102] |
| RASAL2 | Down-regulated | protein_coding | heat shock 27kDa protein 1 [Source:HGNC Symbol;Acc:5246] |
| ACTB | Down-regulated | protein_coding | serpin peptidase inhibitor, clade E (nexin, plasminogen activator inhibitor type 1), member 1 [Source:HGNC Symbol;Acc:8583] |
| RBMS2 | Down-regulated | protein_coding | mesoderm specific transcript [Source:HGNC Symbol;Acc:7028] |
| ANKRD13A | Down-regulated | protein_coding | mesenchyme homeobox 2 [Source:HGNC Symbol;Acc:7014] |
| NFKB2 | Up-regulated | protein_coding | prune homolog 2 (Drosophila) [Source:HGNC Symbol;Acc:25209] |
| PPP1R12B | Down-regulated | protein_coding | asporin [Source:HGNC Symbol;Acc:14872] |
| IL4R | Up-regulated | protein_coding | KN motif and ankyrin repeat domains 1 [Source:HGNC Symbol;Acc:19309] |
| PHF17 | Down-regulated | protein_coding | PDZ and LIM domain 1 [Source:HGNC Symbol;Acc:2067] |
| FAP | Up-regulated | protein_coding | chemokine (C-X-C motif) ligand 12 [Source:HGNC Symbol;Acc:10672] |
| DBC1 | Up-regulated | protein_coding | chromosome 10 open reading frame 54 [Source:HGNC Symbol;Acc:30085] |
| FKBP7 | Up-regulated | protein_coding | actin, alpha 2, smooth muscle, aorta [Source:HGNC Symbol;Acc:130] |
| SLC1A3 | Up-regulated | protein_coding | dickkopf 1 homolog (Xenopus laevis) [Source:HGNC Symbol;Acc:2891] |
| MOXD1 | Down-regulated | protein_coding | vesicle amine transport protein 1 homolog (T. californica) [Source:HGNC Symbol;Acc:16919] |
| COL5A3 | Up-regulated | protein_coding | SMAD specific E3 ubiquitin protein ligase 2 [Source:HGNC Symbol;Acc:16809] |
| CXCL2 | Up-regulated | protein_coding | inositol polyphosphate-4-phosphatase, type II, 105kDa [Source:HGNC Symbol;Acc:6075] |
| ATP8B1 | Down-regulated | protein_coding | Wolf-Hirschhorn syndrome candidate 1 [Source:HGNC Symbol;Acc:12766] |
| COBLL1 | Down-regulated | protein_coding | non-SMC condensin I complex, subunit G [Source:HGNC Symbol;Acc:24304] |
| KCNK2 | Down-regulated | protein_coding | crystallin, alpha B [Source:HGNC Symbol;Acc:2389] |
| CD82 | Up-regulated | protein_coding | Rho guanine nucleotide exchange factor (GEF) 17 [Source:HGNC Symbol;Acc:21726] |
| PREP | Up-regulated | protein_coding | coronin, actin binding protein, 1C [Source:HGNC Symbol;Acc:2254] |
| AKR1B1 | Up-regulated | protein_coding | keratin 18 [Source:HGNC Symbol;Acc:6430] |
| DNAJA1 | Up-regulated | protein_coding | forkhead box M1 [Source:HGNC Symbol;Acc:3818] |
| B4GALT1 | Up-regulated | protein_coding | solute carrier family 38, member 1 [Source:HGNC Symbol;Acc:13447] |
| ALG9 | Up-regulated | protein_coding | G protein-coupled receptor 133 [Source:HGNC Symbol;Acc:19893] |
| ADAMTS2 | Down-regulated | protein_coding | collagen, type XII, alpha 1 [Source:HGNC Symbol;Acc:2188] |
| MMP2 | Up-regulated | protein_coding | neural precursor cell expressed, developmentally down-regulated 9 [Source:HGNC Symbol;Acc:7733] |
| PTHLH | Up-regulated | protein_coding | serine/threonine kinase 38 [Source:HGNC Symbol;Acc:17847] |
| AURKA | Down-regulated | protein_coding | CAP, adenylate cyclase-associated protein, 2 (yeast) [Source:HGNC Symbol;Acc:20039] |
| TPX2 | Down-regulated | protein_coding | single-minded homolog 1 (Drosophila) [Source:HGNC Symbol;Acc:10882] |
| ANKRD10 | Up-regulated | protein_coding | G protein-coupled receptor 126 [Source:HGNC Symbol;Acc:13841] |
| SMOX | Up-regulated | protein_coding | phosphatase and actin regulator 2 [Source:HGNC Symbol;Acc:20956] |
| CPXM1 | Up-regulated | protein_coding | erbb2 interacting protein [Source:HGNC Symbol;Acc:15842] |
| PXN | Down-regulated | protein_coding | 3-hydroxy-3-methylglutaryl-CoA synthase 1 (soluble) [Source:HGNC Symbol;Acc:5007] |
| BIRC5 | Down-regulated | protein_coding | kinesin family member 20A [Source:HGNC Symbol;Acc:9787] |
| PCBP4 | Down-regulated | protein_coding | lysyl oxidase [Source:HGNC Symbol;Acc:6664] |
| ICAM1 | Up-regulated | protein_coding | phosphodiesterase 8B [Source:HGNC Symbol;Acc:8794] |
| IRAK3 | Up-regulated | protein_coding | cadherin 6, type 2, K-cadherin (fetal kidney) [Source:HGNC Symbol;Acc:1765] |
| NRCAM | Up-regulated | protein_coding | lamin B1 [Source:HGNC Symbol;Acc:6637] |
| LAMB1 | Up-regulated | protein_coding | natriuretic peptide receptor C/guanylate cyclase C (atrionatriuretic peptide receptor C) [Source:HGNC Symbol;Acc:7945] |
| ITGA6 | Down-regulated | protein_coding | fibroblast growth factor 1 (acidic) [Source:HGNC Symbol;Acc:3665] |
| SEL1L3 | Up-regulated | protein_coding | stanniocalcin 2 [Source:HGNC Symbol;Acc:11374] |
| ZFHX4 | Down-regulated | protein_coding | angiomotin like 2 [Source:HGNC Symbol;Acc:17812] |
| CCDC80 | Down-regulated | protein_coding | hairy and enhancer of split 1, (Drosophila) [Source:HGNC Symbol;Acc:5192] |
| SLC22A17 | Up-regulated | protein_coding | epithelial cell transforming sequence 2 oncogene [Source:HGNC Symbol;Acc:3155] |
| SEMA6A | Up-regulated | protein_coding | ARP3 actin-related protein 3 homolog (yeast) [Source:HGNC Symbol;Acc:170] |
| EZR | Down-regulated | protein_coding | tumor protein p53 inducible protein 3 [Source:HGNC Symbol;Acc:19373] |
| MYL6 | Down-regulated | protein_coding | integrin, alpha 4 (antigen CD49D, alpha 4 subunit of VLA-4 receptor) [Source:HGNC Symbol;Acc:6140] |
| TMEM38B | Up-regulated | protein_coding | reticulon 4 [Source:HGNC Symbol;Acc:14085] |
| PTGS1 | Up-regulated | protein_coding | EGF containing fibulin-like extracellular matrix protein 1 [Source:HGNC Symbol;Acc:3218] |
| TBC1D2 | Down-regulated | protein_coding | glutaminase [Source:HGNC Symbol;Acc:4331] |
| IL11 | Up-regulated | protein_coding | EF-hand domain family, member D1 [Source:HGNC Symbol;Acc:29556] |
| CREM | Up-regulated | protein_coding | EH domain binding protein 1 [Source:HGNC Symbol;Acc:29144] |
| DSP | Down-regulated | protein_coding | four and a half LIM domains 2 [Source:HGNC Symbol;Acc:3703] |
| ABL1 | Up-regulated | protein_coding | syndecan 1 [Source:HGNC Symbol;Acc:10658] |
| SCD | Down-regulated | protein_coding | par-3 partitioning defective 3 homolog B (C. elegans) [Source:HGNC Symbol;Acc:14446] |
| ABLIM1 | Down-regulated | protein_coding | 24-dehydrocholesterol reductase [Source:HGNC Symbol;Acc:2859] |
| PALM | Down-regulated | protein_coding | Ral GEF with PH domain and SH3 binding motif 2 [Source:HGNC Symbol;Acc:30279] |
| MED15 | Down-regulated | protein_coding | family with sequence similarity 20, member B [Source:HGNC Symbol;Acc:23017] |
| GGT5 | Up-regulated | protein_coding | olfactomedin-like 3 [Source:HGNC Symbol;Acc:24956] |
| MICALL1 | Down-regulated | protein_coding | regulator of G-protein signaling 4 [Source:HGNC Symbol;Acc:10000] |
| SUN2 | Down-regulated | protein_coding | synovial sarcoma, X breakpoint 2 interacting protein [Source:HGNC Symbol;Acc:16509] |
| MCM5 | Down-regulated | protein_coding | guanylate binding protein 1, interferon-inducible [Source:HGNC Symbol;Acc:4182] |
| MYH9 | Down-regulated | protein_coding | inhibitor of DNA binding 3, dominant negative helix-loop-helix protein [Source:HGNC Symbol;Acc:5362] |
| RANGAP1 | Down-regulated | protein_coding | cell division cycle 20 [Source:HGNC Symbol;Acc:1723] |
| PYGL | Down-regulated | protein_coding | coagulation factor III (thromboplastin, tissue factor) [Source:HGNC Symbol;Acc:3541] |
| CNIH | Up-regulated | protein_coding | stathmin 1 [Source:HGNC Symbol;Acc:6510] |
| SPTLC2 | Down-regulated | protein_coding | centromere protein F, 350/400kDa [Source:HGNC Symbol;Acc:1857] |
| RIN3 | Down-regulated | protein_coding | Kruppel-like factor 7 (ubiquitous) [Source:HGNC Symbol;Acc:6350] |
| LGMN | Down-regulated | protein_coding | connective tissue growth factor [Source:HGNC Symbol;Acc:2500] |
| DHRS7 | Up-regulated | protein_coding | myosin, light chain 12B, regulatory [Source:HGNC Symbol;Acc:29827] |
| KIAA0247 | Up-regulated | protein_coding | coiled-coil domain containing 92 [Source:HGNC Symbol;Acc:29563] |
| PCNX | Up-regulated | protein_coding | catenin (cadherin-associated protein), alpha-like 1 [Source:HGNC Symbol;Acc:2512] |
| PCK2 | Down-regulated | protein_coding | PHD finger protein 19 [Source:HGNC Symbol;Acc:24566] |
| NFKBIA | Up-regulated | protein_coding | protein phosphatase 1, regulatory subunit 3C [Source:HGNC Symbol;Acc:9293] |
| PLTP | Up-regulated | protein_coding | methylenetetrahydrofolate dehydrogenase (NADP+ dependent) 1-like [Source:HGNC Symbol;Acc:21055] |
| MMP9 | Up-regulated | protein_coding | acetyl-CoA acetyltransferase 2 [Source:HGNC Symbol;Acc:94] |
| PROCR | Up-regulated | protein_coding | SMAD family member 9 [Source:HGNC Symbol;Acc:6774] |
| MYBL2 | Down-regulated | protein_coding | transforming growth factor, beta-induced, 68kDa [Source:HGNC Symbol;Acc:11771] |
| SLCO4A1 | Up-regulated | protein_coding | thymopoietin [Source:HGNC Symbol;Acc:11875] |
| NTSR1 | Up-regulated | protein_coding | sorbin and SH3 domain containing 3 [Source:HGNC Symbol;Acc:30907] |
| RNF24 | Up-regulated | protein_coding | PDZ and LIM domain 2 (mystique) [Source:HGNC Symbol;Acc:13992] |
| MYL9 | Down-regulated | protein_coding | annexin A11 [Source:HGNC Symbol;Acc:535] |
| JAG1 | Down-regulated | protein_coding | reversion-inducing-cysteine-rich protein with kazal motifs [Source:HGNC Symbol;Acc:11345] |
| CST3 | Up-regulated | protein_coding | caldesmon 1 [Source:HGNC Symbol;Acc:1441] |
| MYL12A | Down-regulated | protein_coding | GIPC PDZ domain containing family, member 1 [Source:HGNC Symbol;Acc:1226] |
| MXRA5 | Down-regulated | protein_coding | neurolysin (metallopeptidase M3 family) [Source:HGNC Symbol;Acc:16058] |
| SRPX | Up-regulated | protein_coding | tubulin, alpha 1b [Source:HGNC Symbol;Acc:18809] |
| PLS3 | Down-regulated | protein_coding | tocopherol (alpha) transfer protein-like [Source:HGNC Symbol;Acc:16114] |
| EEA1 | Down-regulated | protein_coding | prostaglandin I2 (prostacyclin) synthase [Source:HGNC Symbol;Acc:9603] |
| TIMP1 | Up-regulated | protein_coding | serpin peptidase inhibitor, clade B (ovalbumin), member 6 [Source:HGNC Symbol;Acc:8950] |
| SRPX2 | Up-regulated | protein_coding | AHNAK nucleoprotein [Source:HGNC Symbol;Acc:347] |
| SYTL4 | Up-regulated | protein_coding | FBJ murine osteosarcoma viral oncogene homolog B [Source:HGNC Symbol;Acc:3797] |
| ARMCX3 | Up-regulated | protein_coding | vasodilator-stimulated phosphoprotein [Source:HGNC Symbol;Acc:12652] |
| FNDC3A | Up-regulated | protein_coding | destrin (actin depolymerizing factor) [Source:HGNC Symbol;Acc:15750] |
| DNAJC3 | Up-regulated | protein_coding | inhibitor of DNA binding 1, dominant negative helix-loop-helix protein [Source:HGNC Symbol;Acc:5360] |
| UGGT2 | Up-regulated | protein_coding | angiomotin [Source:HGNC Symbol;Acc:17810] |
| FLT1 | Down-regulated | protein_coding | discs, large (Drosophila) homolog-associated protein 5 [Source:HGNC Symbol;Acc:16864] |
| KATNAL1 | Down-regulated | protein_coding | heat shock 70kDa protein 2 [Source:HGNC Symbol;Acc:5235] |
| MEDAG | Up-regulated | protein_coding | Kruppel-like factor 2 (lung) [Source:HGNC Symbol;Acc:6347] |
| TSC22D1 | Up-regulated | protein_coding | ZFP36 ring finger protein [Source:HGNC Symbol;Acc:12862] |
| HCFC1R1 | Down-regulated | protein_coding | tyrosine 3-monooxygenase/tryptophan 5-monooxygenase activation protein, eta polypeptide [Source:HGNC Symbol;Acc:12853] |
| WFDC1 | Down-regulated | protein_coding | CDC42 effector protein (Rho GTPase binding) 1 [Source:HGNC Symbol;Acc:17014] |
| COTL1 | Down-regulated | protein_coding | carboxypeptidase A4 [Source:HGNC Symbol;Acc:15740] |
| CRISPLD2 | Up-regulated | protein_coding | podocalyxin-like [Source:HGNC Symbol;Acc:9171] |
| PIEZO1 | Down-regulated | protein_coding | filamin C, gamma [Source:HGNC Symbol;Acc:3756] |
| CPPED1 | Down-regulated | protein_coding | tropomodulin 2 (neuronal) [Source:HGNC Symbol;Acc:11872] |
| SALL1 | Up-regulated | protein_coding | family with sequence similarity 63, member B [Source:HGNC Symbol;Acc:26954] |
| RASL12 | Up-regulated | protein_coding | chemokine (C-C motif) receptor-like 1 [Source:HGNC Symbol;Acc:1611] |
| IGDCC4 | Up-regulated | protein_coding | palladin, cytoskeletal associated protein [Source:HGNC Symbol;Acc:17068] |
| CD276 | Up-regulated | protein_coding | family with sequence similarity 64, member A [Source:HGNC Symbol;Acc:25483] |
| KIAA1199 | Up-regulated | protein_coding | solute carrier family 44, member 2 [Source:HGNC Symbol;Acc:17292] |
| EHD4 | Down-regulated | protein_coding | ajuba LIM protein [Source:HGNC Symbol;Acc:20250] |
| ZDHHC2 | Down-regulated | protein_coding | low density lipoprotein receptor [Source:HGNC Symbol;Acc:6547] |
| RIPK2 | Up-regulated | protein_coding | calponin 1, basic, smooth muscle [Source:HGNC Symbol;Acc:2155] |
| SFRP1 | Up-regulated | protein_coding | actinin, alpha 4 [Source:HGNC Symbol;Acc:166] |
| PLAT | Up-regulated | protein_coding | H19, imprinted maternally expressed transcript (non-protein coding) [Source:HGNC Symbol;Acc:4713] |
| NDRG1 | Up-regulated | protein_coding | neuronal PAS domain protein 1 [Source:HGNC Symbol;Acc:7894] |
| STMN2 | Down-regulated | protein_coding | A kinase (PRKA) anchor protein 12 [Source:HGNC Symbol;Acc:370] |
| TRPS1 | Up-regulated | protein_coding | spectrin repeat containing, nuclear envelope 1 [Source:HGNC Symbol;Acc:17089] |
| SH2D4A | Down-regulated | protein_coding | CAP, adenylate cyclase-associated protein 1 (yeast) [Source:HGNC Symbol;Acc:20040] |
| SLC39A14 | Up-regulated | protein_coding | diaphanous homolog 1 (Drosophila) [Source:HGNC Symbol;Acc:2876] |
| ARHGEF10 | Down-regulated | protein_coding | microtubule-associated protein 1B [Source:HGNC Symbol;Acc:6836] |
| MAN2B1 | Up-regulated | protein_coding | keratin 34 [Source:HGNC Symbol;Acc:6452] |
| ECH1 | Up-regulated | protein_coding | matrilin 2 [Source:HGNC Symbol;Acc:6908] |
| RELB | Up-regulated | protein_coding | KN motif and ankyrin repeat domains 4 [Source:HGNC Symbol;Acc:27263] |
| FCGRT | Up-regulated | protein_coding | myosin, heavy chain 10, non-muscle [Source:HGNC Symbol;Acc:7568] |
| PPP1R13L | Down-regulated | protein_coding | myosin phosphatase Rho interacting protein [Source:HGNC Symbol;Acc:30321] |
| ERCC2 | Down-regulated | protein_coding | periostin, osteoblast specific factor [Source:HGNC Symbol;Acc:16953] |
| GLTSCR2 | Up-regulated | protein_coding | myosin, heavy chain 11, smooth muscle [Source:HGNC Symbol;Acc:7569] |
| CLEC11A | Up-regulated | protein_coding | SWAP switching B-cell complex 70kDa subunit [Source:HGNC Symbol;Acc:17070] |
| HAS1 | Up-regulated | protein_coding | microtubule associated monooxygenase, calponin and LIM domain containing 2 [Source:HGNC Symbol;Acc:24693] |
| TMEM205 | Up-regulated | protein_coding | related RAS viral (r-ras) oncogene homolog 2 [Source:HGNC Symbol;Acc:17271] |
| ARRDC2 | Up-regulated | protein_coding | CBP80/20-dependent translation initiation factor [Source:HGNC Symbol;Acc:23925] |
| CDK6 | Down-regulated | protein_coding | cyclin B1 [Source:HGNC Symbol;Acc:1579] |
| TFPI2 | Up-regulated | protein_coding | Meis homeobox 2 [Source:HGNC Symbol;Acc:7001] |
| NAMPT | Up-regulated | protein_coding | proline/serine-rich coiled-coil 1 [Source:HGNC Symbol;Acc:24472] |
| HBP1 | Up-regulated | protein_coding | sortilin 1 [Source:HGNC Symbol;Acc:11186] |
| PTN | Up-regulated | protein_coding | nerve growth factor (beta polypeptide) [Source:HGNC Symbol;Acc:7808] |
| CAV2 | Down-regulated | protein_coding | transmembrane protein 106C [Source:HGNC Symbol;Acc:28775] |
| CAV1 | Down-regulated | protein_coding | solute carrier family 38, member 2 [Source:HGNC Symbol;Acc:13448] |
| MET | Down-regulated | protein_coding | Rho-associated, coiled-coil containing protein kinase 2 [Source:HGNC Symbol;Acc:10252] |
| WNT2 | Down-regulated | protein_coding | SPOC domain containing 1 [Source:HGNC Symbol;Acc:26338] |
| HOXA13 | Down-regulated | protein_coding | cell division cycle associated 8 [Source:HGNC Symbol;Acc:14629] |
| HSPB1 | Down-regulated | protein_coding | fatty acid desaturase 2 [Source:HGNC Symbol;Acc:3575] |
| SERPINE1 | Down-regulated | protein_coding | annexin A1 [Source:HGNC Symbol;Acc:533] |
| MEST | Down-regulated | protein_coding | ADAM metallopeptidase domain 19 [Source:HGNC Symbol;Acc:197] |
| MEOX2 | Down-regulated | protein_coding | integrin, alpha 7 [Source:HGNC Symbol;Acc:6143] |
| TSPAN13 | Up-regulated | protein_coding | keratin 7 [Source:HGNC Symbol;Acc:6445] |
| PRUNE2 | Down-regulated | protein_coding | microtubule associated monooxygenase, calponin and LIM domain containing 1 [Source:HGNC Symbol;Acc:20619] |
| ASPN | Down-regulated | protein_coding | dedicator of cytokinesis 10 [Source:HGNC Symbol;Acc:23479] |
| DNM1 | Up-regulated | protein_coding | plexin C1 [Source:HGNC Symbol;Acc:9106] |
| KANK1 | Down-regulated | protein_coding | adaptor protein, phosphotyrosine interaction, PH domain and leucine zipper containing 2 [Source:HGNC Symbol;Acc:18242] |
| GLIS3 | Up-regulated | protein_coding | DNA-damage regulated autophagy modulator 1 [Source:HGNC Symbol;Acc:25645] |
| BAG1 | Up-regulated | protein_coding | filamin B, beta [Source:HGNC Symbol;Acc:3755] |
| PDLIM1 | Down-regulated | protein_coding | cytoskeleton associated protein 2 [Source:HGNC Symbol;Acc:1990] |
| CXCL12 | Down-regulated | protein_coding | LIM domain 7 [Source:HGNC Symbol;Acc:6646] |
| C10orf54 | Down-regulated | protein_coding | tensin 3 [Source:HGNC Symbol;Acc:21616] |
| ACTA2 | Down-regulated | protein_coding | ADAM metallopeptidase with thrombospondin type 1 motif, 7 [Source:HGNC Symbol;Acc:223] |
| SUFU | Up-regulated | protein_coding | nipsnap homolog 3A (C. elegans) [Source:HGNC Symbol;Acc:23619] |
| DKK1 | Down-regulated | protein_coding | ectonucleotide pyrophosphatase/phosphodiesterase 2 [Source:HGNC Symbol;Acc:3357] |
| RPL28 | Up-regulated | protein_coding | talin 1 [Source:HGNC Symbol;Acc:11845] |
| CSF3 | Up-regulated | protein_coding | transcription factor 19 [Source:HGNC Symbol;Acc:11629] |
| RASD1 | Up-regulated | protein_coding | flotillin 1 [Source:HGNC Symbol;Acc:3757] |
| LGALS3BP | Up-regulated | protein_coding | synaptotagmin-like 2 [Source:HGNC Symbol;Acc:15585] |
| CCL7 | Up-regulated | protein_coding | sulfatase 1 [Source:HGNC Symbol;Acc:20391] |
| CCL2 | Up-regulated | protein_coding | transient receptor potential cation channel, subfamily C, member 6 [Source:HGNC Symbol;Acc:12338] |
| VAT1 | Down-regulated | protein_coding | thrombospondin 1 [Source:HGNC Symbol;Acc:11785] |
| SMURF2 | Down-regulated | protein_coding | kinesin family member 23 [Source:HGNC Symbol;Acc:6392] |
| SLC16A6 | Up-regulated | protein_coding | integrin, alpha 11 [Source:HGNC Symbol;Acc:6136] |
| PRKAR1A | Up-regulated | protein_coding | cancer susceptibility candidate 5 [Source:HGNC Symbol;Acc:24054] |
| FAM20A | Up-regulated | protein_coding | uveal autoantigen with coiled-coil domains and ankyrin repeats [Source:HGNC Symbol;Acc:15947] |
| MMD | Up-regulated | protein_coding | SMAD family member 6 [Source:HGNC Symbol;Acc:6772] |
| INPP4B | Down-regulated | protein_coding | breast cancer anti-estrogen resistance 3 [Source:HGNC Symbol;Acc:973] |
| KLHL2 | Up-regulated | protein_coding | Rho GTPase activating protein 29 [Source:HGNC Symbol;Acc:30207] |
| CPE | Up-regulated | protein_coding | myoferlin [Source:HGNC Symbol;Acc:3656] |
| WHSC1 | Down-regulated | protein_coding | lysyl oxidase-like 4 [Source:HGNC Symbol;Acc:17171] |
| KLHL5 | Up-regulated | protein_coding | kinesin family member 11 [Source:HGNC Symbol;Acc:6388] |
| NCAPG | Down-regulated | protein_coding | centrosomal protein 55kDa [Source:HGNC Symbol;Acc:1161] |
| PPARGC1A | Up-regulated | protein_coding | phospholipase C, epsilon 1 [Source:HGNC Symbol;Acc:17175] |
| CRYAB | Down-regulated | protein_coding | myopalladin [Source:HGNC Symbol;Acc:23246] |
| PANX1 | Up-regulated | protein_coding | HECT and RLD domain containing E3 ubiquitin protein ligase 3 [Source:HGNC Symbol;Acc:4876] |
| ARHGEF17 | Down-regulated | protein_coding | fibroblast growth factor 5 [Source:HGNC Symbol;Acc:3683] |
| SLC1A2 | Up-regulated | protein_coding | phosphodiesterase 5A, cGMP-specific [Source:HGNC Symbol;Acc:8784] |
| PITPNM1 | Up-regulated | protein_coding | septin 11 [Source:HGNC Symbol;Acc:25589] |
| CORO1C | Down-regulated | protein_coding | shroom family member 3 [Source:HGNC Symbol;Acc:30422] |
| TSPAN11 | Up-regulated | protein_coding | fibrillin 2 [Source:HGNC Symbol;Acc:3604] |
| KRT18 | Down-regulated | protein_coding | adhesion molecule with Ig-like domain 2 [Source:HGNC Symbol;Acc:24073] |
| FOXM1 | Down-regulated | protein_coding | GLI pathogenesis-related 1 [Source:HGNC Symbol;Acc:17001] |
| SH2B3 | Up-regulated | protein_coding | leucine-rich repeat containing G protein-coupled receptor 5 [Source:HGNC Symbol;Acc:4504] |
| SLC38A1 | Down-regulated | protein_coding | neural precursor cell expressed, developmentally down-regulated 1 [Source:HGNC Symbol;Acc:7723] |
| FZD10 | Up-regulated | protein_coding | solute carrier family 7 (cationic amino acid transporter, y+ system), member 1 [Source:HGNC Symbol;Acc:11057] |
| GPR133 | Down-regulated | protein_coding | extended synaptotagmin-like protein 1 [Source:HGNC Symbol;Acc:29534] |
| C12orf57 | Up-regulated | protein_coding | diaphanous homolog 3 (Drosophila) [Source:HGNC Symbol;Acc:15480] |
| COL12A1 | Down-regulated | protein_coding | muscleblind-like splicing regulator 2 [Source:HGNC Symbol;Acc:16746] |
| NEDD9 | Down-regulated | protein_coding | somatostatin receptor 1 [Source:HGNC Symbol;Acc:11330] |
| MAN1A1 | Up-regulated | protein_coding | FERM domain containing 6 [Source:HGNC Symbol;Acc:19839] |
| NCOA7 | Up-regulated | protein_coding | fibulin 5 [Source:HGNC Symbol;Acc:3602] |
| STK38 | Down-regulated | protein_coding | tropomyosin 1 (alpha) [Source:HGNC Symbol;Acc:12010] |
| SOD2 | Up-regulated | protein_coding | arrestin domain containing 4 [Source:HGNC Symbol;Acc:28087] |
| BACH2 | Up-regulated | protein_coding | transforming growth factor beta 1 induced transcript 1 [Source:HGNC Symbol;Acc:11767] |
| CAP2 | Down-regulated | protein_coding | Rho GTPase activating protein 17 [Source:HGNC Symbol;Acc:18239] |
| PTP4A1 | Up-regulated | protein_coding | NLR family, CARD domain containing 5 [Source:HGNC Symbol;Acc:29933] |
| SIM1 | Down-regulated | protein_coding | TBC/LysM-associated domain containing 1 [Source:HGNC Symbol;Acc:29325] |
| GPR126 | Down-regulated | protein_coding | myocardin [Source:HGNC Symbol;Acc:16067] |
| PHACTR2 | Down-regulated | protein_coding | v-erb-b2 erythroblastic leukemia viral oncogene homolog 2, neuro/glioblastoma derived oncogene homolog (avian) [Source:HGNC Symbol;Acc:3430] |
| GMDS | Up-regulated | protein_coding | dopey family member 2 [Source:HGNC Symbol;Acc:1291] |
| VEGFA | Up-regulated | protein_coding | Wilms tumor 1 interacting protein [Source:HGNC Symbol;Acc:20964] |
| FAM46A | Up-regulated | protein_coding | EPH receptor A2 [Source:HGNC Symbol;Acc:3386] |
| ERBB2IP | Down-regulated | protein_coding | SH3 domain binding glutamic acid-rich protein like 3 [Source:HGNC Symbol;Acc:15568] |
| SEMA5A | Up-regulated | protein_coding | heparan sulfate proteoglycan 2 [Source:HGNC Symbol;Acc:5273] |
| HMGCS1 | Down-regulated | protein_coding | cysteine-rich, angiogenic inducer, 61 [Source:HGNC Symbol;Acc:2654] |
| KIF20A | Down-regulated | protein_coding | tubulointerstitial nephritis antigen-like 1 [Source:HGNC Symbol;Acc:19168] |
| LOX | Down-regulated | protein_coding | protein tyrosine phosphatase, receptor type, F [Source:HGNC Symbol;Acc:9670] |
| PDE8B | Down-regulated | protein_coding | calmodulin 2 (phosphorylase kinase, delta) [Source:HGNC Symbol;Acc:1445] |
| CDH6 | Down-regulated | protein_coding | AF4/FMR2 family, member 3 [Source:HGNC Symbol;Acc:6473] |
| LMNB1 | Down-regulated | protein_coding | membrane-associated ring finger (C3HC4) 4, E3 ubiquitin protein ligase [Source:HGNC Symbol;Acc:29269] |
| ARRDC3 | Up-regulated | protein_coding | pleckstrin homology-like domain, family B, member 2 [Source:HGNC Symbol;Acc:29573] |
| NPR3 | Down-regulated | protein_coding | neutral cholesterol ester hydrolase 1 [Source:HGNC Symbol;Acc:29260] |
| PDE4D | Up-regulated | protein_coding | KIAA0226 [Source:HGNC Symbol;Acc:28991] |
| FGF1 | Down-regulated | protein_coding | T-cell leukemia translocation altered [Source:HGNC Symbol;Acc:11692] |
| PDGFRB | Up-regulated | protein_coding | slit homolog 2 (Drosophila) [Source:HGNC Symbol;Acc:11086] |
| STC2 | Down-regulated | protein_coding | EPH receptor A5 [Source:HGNC Symbol;Acc:3389] |
| CPEB4 | Up-regulated | protein_coding | cyclin A2 [Source:HGNC Symbol;Acc:1578] |
| SELK | Up-regulated | protein_coding | ubiquitin specific peptidase 53 [Source:HGNC Symbol;Acc:29255] |
| AMOTL2 | Down-regulated | protein_coding | myosin X [Source:HGNC Symbol;Acc:7593] |
| WNT5A | Up-regulated | protein_coding | S-phase kinase-associated protein 2, E3 ubiquitin protein ligase [Source:HGNC Symbol;Acc:10901] |
| PFKFB4 | Up-regulated | protein_coding | teneurin transmembrane protein 2 [Source:HGNC Symbol;Acc:29943] |
| COL7A1 | Up-regulated | protein_coding | dishevelled associated activator of morphogenesis 2 [Source:HGNC Symbol;Acc:18143] |
| HES1 | Down-regulated | protein_coding | signal peptide, CUB domain, EGF-like 3 [Source:HGNC Symbol;Acc:13655] |
| ECT2 | Down-regulated | protein_coding | Rho GTPase activating protein 18 [Source:HGNC Symbol;Acc:21035] |
| EFCC1 | Up-regulated | protein_coding | insulin-like growth factor binding protein 3 [Source:HGNC Symbol;Acc:5472] |
| CCL20 | Up-regulated | protein_coding | non-SMC condensin II complex, subunit G2 [Source:HGNC Symbol;Acc:21904] |
| ACTR3 | Down-regulated | protein_coding | transmembrane protein 47 [Source:HGNC Symbol;Acc:18515] |
| TP53I3 | Down-regulated | protein_coding | solute carrier family 16, member 2 (thyroid hormone transporter) [Source:HGNC Symbol;Acc:10923] |
| ACVR1 | Up-regulated | protein_coding | phosphoribosyl pyrophosphate synthetase 1 [Source:HGNC Symbol;Acc:9462] |
| FNDC4 | Up-regulated | protein_coding | chromosome 9 open reading frame 3 [Source:HGNC Symbol;Acc:1361] |
| ITGA4 | Down-regulated | protein_coding | stomatin [Source:HGNC Symbol;Acc:3383] |
| RTN4 | Down-regulated | protein_coding | Ras suppressor protein 1 [Source:HGNC Symbol;Acc:10464] |
| EFEMP1 | Down-regulated | protein_coding | HECT and RLD domain containing E3 ubiquitin protein ligase 4 [Source:HGNC Symbol;Acc:24521] |
| GLS | Down-regulated | protein_coding | calcium/calmodulin-dependent protein kinase II gamma [Source:HGNC Symbol;Acc:1463] |
| IGFBP2 | Up-regulated | protein_coding | ankyrin repeat domain 1 (cardiac muscle) [Source:HGNC Symbol;Acc:15819] |
| IGFBP5 | Up-regulated | protein_coding | antigen identified by monoclonal antibody Ki-67 [Source:HGNC Symbol;Acc:7107] |
| EFHD1 | Down-regulated | protein_coding | endonuclease domain containing 1 [Source:HGNC Symbol;Acc:29129] |
| EHBP1 | Down-regulated | protein_coding | ADAM metallopeptidase domain 33 [Source:HGNC Symbol;Acc:15478] |
| KDM3A | Up-regulated | protein_coding | kin of IRRE like 3 (Drosophila) [Source:HGNC Symbol;Acc:23204] |
| IL1R1 | Up-regulated | protein_coding | transgelin [Source:HGNC Symbol;Acc:11553] |
| IL1RL1 | Up-regulated | protein_coding | junctophilin 2 [Source:HGNC Symbol;Acc:14202] |
| IL18R1 | Up-regulated | protein_coding | KIAA1755 [Source:HGNC Symbol;Acc:29372] |
| FHL2 | Down-regulated | protein_coding | phospholipase C, beta 3 (phosphatidylinositol-specific) [Source:HGNC Symbol;Acc:9056] |
| QPCT | Up-regulated | protein_coding | mohawk homeobox [Source:HGNC Symbol;Acc:23729] |
| SDC1 | Down-regulated | protein_coding | adrenoceptor alpha 2A [Source:HGNC Symbol;Acc:281] |
| PARD3B | Down-regulated | protein_coding | vascular endothelial growth factor C [Source:HGNC Symbol;Acc:12682] |
| DHCR24 | Down-regulated | protein_coding | protease, serine, 23 [Source:HGNC Symbol;Acc:14370] |
| RALGPS2 | Down-regulated | protein_coding | cysteine rich transmembrane BMP regulator 1 (chordin-like) [Source:HGNC Symbol;Acc:2359] |
| FAM20B | Down-regulated | protein_coding | calcium channel, voltage-dependent, L type, alpha 1C subunit [Source:HGNC Symbol;Acc:1390] |
| ERRFI1 | Up-regulated | protein_coding | membrane associated guanylate kinase, WW and PDZ domain containing 1 [Source:HGNC Symbol;Acc:946] |
| EDEM3 | Up-regulated | protein_coding | NIMA-related kinase 7 [Source:HGNC Symbol;Acc:13386] |
| LEPR | Up-regulated | protein_coding | epidermal growth factor receptor pathway substrate 8 [Source:HGNC Symbol;Acc:3420] |
| PRG4 | Up-regulated | protein_coding | WW and C2 domain containing 2 [Source:HGNC Symbol;Acc:24148] |
| PLA2G4A | Up-regulated | protein_coding | solute carrier family 25 (mitochondrial carrier; adenine nucleotide translocator), member 4 [Source:HGNC Symbol;Acc:10990] |
| WLS | Up-regulated | protein_coding | dystonin [Source:HGNC Symbol;Acc:1090] |
| RGS2 | Up-regulated | protein_coding | protein tyrosine phosphatase, non-receptor type 14 [Source:HGNC Symbol;Acc:9647] |
| OLFML3 | Down-regulated | protein_coding | ectopic P-granules autophagy protein 5 homolog (C. elegans) [Source:HGNC Symbol;Acc:29331] |
| MFAP2 | Up-regulated | protein_coding | muscleblind-like splicing regulator 1 [Source:HGNC Symbol;Acc:6923] |
| RGS4 | Down-regulated | protein_coding | FERM, RhoGEF (ARHGEF) and pleckstrin domain protein 1 (chondrocyte-derived) [Source:HGNC Symbol;Acc:3591] |
| SSX2IP | Down-regulated | protein_coding | utrophin [Source:HGNC Symbol;Acc:12635] |
| GBP1 | Down-regulated | protein_coding | Dab, mitogen-responsive phosphoprotein, homolog 2 (Drosophila) [Source:HGNC Symbol;Acc:2662] |
| ID3 | Down-regulated | protein_coding | dimethylarginine dimethylaminohydrolase 1 [Source:HGNC Symbol;Acc:2715] |
| CDC20 | Down-regulated | protein_coding | sema domain, immunoglobulin domain (Ig), short basic domain, secreted, (semaphorin) 3D [Source:HGNC Symbol;Acc:10726] |
| TMED5 | Up-regulated | protein_coding | adenylate kinase 5 [Source:HGNC Symbol;Acc:365] |
| F3 | Down-regulated | protein_coding | Thy-1 cell surface antigen [Source:HGNC Symbol;Acc:11801] |
| HSD11B1 | Up-regulated | protein_coding | SH3 domain containing ring finger 1 [Source:HGNC Symbol;Acc:17650] |
| STMN1 | Down-regulated | protein_coding | family with sequence similarity 69, member A [Source:HGNC Symbol;Acc:32213] |
| CENPF | Down-regulated | protein_coding | ADAM metallopeptidase with thrombospondin type 1 motif, 1 [Source:HGNC Symbol;Acc:217] |
| CTSD | Up-regulated | protein_coding | vesicular, overexpressed in cancer, prosurvival protein 1 [Source:HGNC Symbol;Acc:34518] |
| NRP2 | Up-regulated | protein_coding | myristoylated alanine-rich protein kinase C substrate [Source:HGNC Symbol;Acc:6759] |
| KLF7 | Down-regulated | protein_coding | GRAM domain containing 3 [Source:HGNC Symbol;Acc:24911] |
| TNFAIP3 | Up-regulated | protein_coding | frizzled family receptor 7 [Source:HGNC Symbol;Acc:4045] |
| CTGF | Down-regulated | protein_coding | formin 2 [Source:HGNC Symbol;Acc:14074] |
| MYL12B | Down-regulated | protein_coding | mitochondrial calcium uniporter [Source:HGNC Symbol;Acc:23526] |
| FOXO3 | Up-regulated | protein_coding | growth differentiation factor 6 [Source:HGNC Symbol;Acc:4221] |
| KLF9 | Up-regulated | protein_coding | myosin IE [Source:HGNC Symbol;Acc:7599] |
| CCDC92 | Down-regulated | protein_coding | tetraspanin 18 [Source:HGNC Symbol;Acc:20660] |
| CTNNAL1 | Down-regulated | protein_coding | cAMP responsive element binding protein 3-like 1 [Source:HGNC Symbol;Acc:18856] |
| PHF19 | Down-regulated | protein_coding | aggrecan [Source:HGNC Symbol;Acc:319] |
| NR4A3 | Up-regulated | protein_coding | xanthine dehydrogenase [Source:HGNC Symbol;Acc:12805] |
| PGF | Up-regulated | protein_coding | muscle RAS oncogene homolog [Source:HGNC Symbol;Acc:7227] |
| IRF2BPL | Up-regulated | protein_coding | collectin sub-family member 12 [Source:HGNC Symbol;Acc:16016] |
| TGFB3 | Up-regulated | protein_coding | cullin 4B [Source:HGNC Symbol;Acc:2555] |
| PPP1R3C | Down-regulated | protein_coding | cysteine and glycine-rich protein 1 [Source:HGNC Symbol;Acc:2469] |
| MSX2 | Up-regulated | protein_coding | actin, alpha, cardiac muscle 1 [Source:HGNC Symbol;Acc:143] |
| MTHFD1L | Down-regulated | protein_coding | zyxin [Source:HGNC Symbol;Acc:13200] |
| ACAT2 | Down-regulated | protein_coding | cystathionine-beta-synthase [Source:HGNC Symbol;Acc:1550] |
| SMAD9 | Down-regulated | protein_coding | proprotein convertase subtilisin/kexin type 7 [Source:HGNC Symbol;Acc:8748] |
| TGFBI | Down-regulated | protein_coding | lymphocyte antigen 6 complex, locus K [Source:HGNC Symbol;Acc:24225] |
| TMPO | Down-regulated | protein_coding | phospholipase C, delta 3 [Source:HGNC Symbol;Acc:9061] |
| DUSP4 | Up-regulated | protein_coding | Rac GTPase activating protein 1 [Source:HGNC Symbol;Acc:9804] |
| CLU | Up-regulated | protein_coding | SPC24, NDC80 kinetochore complex component, homolog (S. cerevisiae) [Source:HGNC Symbol;Acc:26913] |
| SORBS3 | Down-regulated | protein_coding | adenylate cyclase 9 [Source:HGNC Symbol;Acc:240] |
| PDLIM2 | Down-regulated | protein_coding | ribosomal protein S6 kinase, 90kDa, polypeptide 4 [Source:HGNC Symbol;Acc:10433] |
| TBX2 | Up-regulated | protein_coding | dehydrogenase/reductase (SDR family) member 3 [Source:HGNC Symbol;Acc:17693] |
| TSHZ3 | Up-regulated | protein_coding | syndecan 3 [Source:HGNC Symbol;Acc:10660] |
| ANXA11 | Down-regulated | protein_coding | syncoilin, intermediate filament protein [Source:HGNC Symbol;Acc:28897] |
| FAM213A | Up-regulated | protein_coding | alkaline phosphatase, liver/bone/kidney [Source:HGNC Symbol;Acc:438] |
| PTGFR | Up-regulated | protein_coding | multiple EGF-like-domains 6 [Source:HGNC Symbol;Acc:3232] |
| INHBA | Up-regulated | protein_coding | nexilin (F actin binding protein) [Source:HGNC Symbol;Acc:29557] |
| TWIST1 | Up-regulated | protein_coding | DnaJ (Hsp40) homolog, subfamily B, member 4 [Source:HGNC Symbol;Acc:14886] |
| RECK | Down-regulated | protein_coding | sorting nexin 7 [Source:HGNC Symbol;Acc:14971] |
| CALD1 | Down-regulated | protein_coding | netrin G1 [Source:HGNC Symbol;Acc:23319] |
| PLAU | Up-regulated | protein_coding | actin related protein 2/3 complex, subunit 5, 16kDa [Source:HGNC Symbol;Acc:708] |
| SRGN | Up-regulated | protein_coding | phosphoprotein enriched in astrocytes 15 [Source:HGNC Symbol;Acc:8822] |
| EGR2 | Up-regulated | protein_coding | kinesin family member 26B [Source:HGNC Symbol;Acc:25484] |
| P4HA1 | Up-regulated | protein_coding | actin, gamma 2, smooth muscle, enteric [Source:HGNC Symbol;Acc:145] |
| RASSF8 | Up-regulated | protein_coding | structural maintenance of chromosomes 6 [Source:HGNC Symbol;Acc:20466] |
| BHLHE41 | Up-regulated | protein_coding | PDZ and LIM domain 5 [Source:HGNC Symbol;Acc:17468] |
| PRDX4 | Up-regulated | protein_coding | CDC42 effector protein (Rho GTPase binding) 3 [Source:HGNC Symbol;Acc:16943] |
| GIPC1 | Down-regulated | protein_coding | leiomodin 1 (smooth muscle) [Source:HGNC Symbol;Acc:6647] |
| NLN | Down-regulated | protein_coding | neurofascin [Source:HGNC Symbol;Acc:29866] |
| MMP19 | Up-regulated | protein_coding | protein tyrosine phosphatase, non-receptor type 13 (APO-1/CD95 (Fas)-associated phosphatase) [Source:HGNC Symbol;Acc:9646] |
| NR4A1 | Up-regulated | protein_coding | pentraxin 3, long [Source:HGNC Symbol;Acc:9692] |
| TUBA1B | Down-regulated | protein_coding | amyloid beta (A4) precursor protein-binding, family B, member 2 [Source:HGNC Symbol;Acc:582] |
| TNFAIP6 | Up-regulated | protein_coding | sphingomyelin synthase 2 [Source:HGNC Symbol;Acc:28395] |
| G0S2 | Up-regulated | protein_coding | protease, serine, 12 (neurotrypsin, motopsin) [Source:HGNC Symbol;Acc:9477] |
| FAM210B | Up-regulated | protein_coding | high mobility group box 2 [Source:HGNC Symbol;Acc:5000] |
| TTPAL | Down-regulated | protein_coding | StAR-related lipid transfer (START) domain containing 4 [Source:HGNC Symbol;Acc:18058] |
| PREX1 | Up-regulated | protein_coding | Rho-related BTB domain containing 3 [Source:HGNC Symbol;Acc:18757] |
| PTGIS | Down-regulated | protein_coding | glutamate receptor, ionotropic, kainate 2 [Source:HGNC Symbol;Acc:4580] |
| PMEPA1 | Up-regulated | protein_coding | Cbp/p300-interacting transactivator, with Glu/Asp-rich carboxy-terminal domain, 2 [Source:HGNC Symbol;Acc:1987] |
| RNF114 | Up-regulated | protein_coding | discoidin, CUB and LCCL domain containing 1 [Source:HGNC Symbol;Acc:21479] |
| NDP | Up-regulated | protein_coding | serine/threonine kinase 17a [Source:HGNC Symbol;Acc:11395] |
| SERPINB6 | Down-regulated | protein_coding | UDP-N-acetyl-alpha-D-galactosamine:polypeptide N-acetylgalactosaminyltransferase 10 (GalNAc-T10) [Source:HGNC Symbol;Acc:19873] |
| TREM1 | Up-regulated | protein_coding | somatomedin B and thrombospondin, type 1 domain containing [Source:HGNC Symbol;Acc:30362] |
| COL21A1 | Up-regulated | protein_coding | aldehyde dehydrogenase 7 family, member A1 [Source:HGNC Symbol;Acc:877] |
| SOX4 | Up-regulated | protein_coding | odd-skipped related 2 (Drosophila) [Source:HGNC Symbol;Acc:15830] |
| CXCL6 | Up-regulated | protein_coding | aldehyde dehydrogenase 1 family, member A1 [Source:HGNC Symbol;Acc:402] |
| EREG | Up-regulated | protein_coding | sushi, von Willebrand factor type A, EGF and pentraxin domain containing 1 [Source:HGNC Symbol;Acc:15985] |
| AHNAK | Down-regulated | protein_coding | maternal embryonic leucine zipper kinase [Source:HGNC Symbol;Acc:16870] |
| SH3TC1 | Up-regulated | protein_coding | cofilin 2 (muscle) [Source:HGNC Symbol;Acc:1875] |
| MT2A | Up-regulated | protein_coding | KIAA1462 [Source:HGNC Symbol;Acc:29283] |
| IRF1 | Up-regulated | protein_coding | protein tyrosine phosphatase-like (proline instead of catalytic arginine), member A [Source:HGNC Symbol;Acc:9639] |
| HS3ST3B1 | Up-regulated | protein_coding | G protein-coupled receptor 176 [Source:HGNC Symbol;Acc:32370] |
| IL1B | Up-regulated | protein_coding | creatine kinase, brain [Source:HGNC Symbol;Acc:1991] |
| C3 | Up-regulated | protein_coding | cytochrome b5 type A (microsomal) [Source:HGNC Symbol;Acc:2570] |
| FOSB | Down-regulated | protein_coding | serpin peptidase inhibitor, clade B (ovalbumin), member 7 [Source:HGNC Symbol;Acc:13902] |
| VASP | Down-regulated | protein_coding | neuron navigator 2 [Source:HGNC Symbol;Acc:15997] |
| GPCPD1 | Up-regulated | protein_coding | polo-like kinase 1 [Source:HGNC Symbol;Acc:9077] |
| BMP2 | Up-regulated | protein_coding | myotubularin related protein 10 [Source:HGNC Symbol;Acc:25999] |
| DSTN | Down-regulated | protein_coding | gremlin 1, DAN family BMP antagonist [Source:HGNC Symbol;Acc:2001] |
| ID1 | Down-regulated | protein_coding | SMAD family member 3 [Source:HGNC Symbol;Acc:6769] |
| AMOT | Down-regulated | protein_coding | microtubule-associated protein 1A [Source:HGNC Symbol;Acc:6835] |
| NR1D1 | Up-regulated | protein_coding | tubulin, alpha 1a [Source:HGNC Symbol;Acc:20766] |
| IFI6 | Up-regulated | protein_coding | tubulin, alpha 1c [Source:HGNC Symbol;Acc:20768] |
| DLGAP5 | Down-regulated | protein_coding | AXL receptor tyrosine kinase [Source:HGNC Symbol;Acc:905] |
| HSPA2 | Down-regulated | protein_coding | protein phosphatase 1, regulatory (inhibitor) subunit 14A [Source:HGNC Symbol;Acc:14871] |
| EMR2 | Up-regulated | protein_coding | death-associated protein kinase 3 [Source:HGNC Symbol;Acc:2676] |
| KLF2 | Down-regulated | protein_coding | insulin-like growth factor binding protein 6 [Source:HGNC Symbol;Acc:5475] |
| TNFRSF19 | Up-regulated | protein_coding | thymidine kinase 1, soluble [Source:HGNC Symbol;Acc:11830] |
| GNG11 | Up-regulated | protein_coding | tetratricopeptide repeat domain 39C [Source:HGNC Symbol;Acc:26595] |
| POR | Up-regulated | protein_coding | filamin A interacting protein 1-like [Source:HGNC Symbol;Acc:24589] |
| ZFP36 | Down-regulated | protein_coding | tenascin XB [Source:HGNC Symbol;Acc:11976] |
| YWHAH | Down-regulated | protein_coding | serum deprivation response [Source:HGNC Symbol;Acc:10690] |
| A4GALT | Up-regulated | protein_coding | SOGA family member 2 [Source:HGNC Symbol;Acc:29121] |
| CDC42EP1 | Down-regulated | protein_coding | interleukin 7 receptor [Source:HGNC Symbol;Acc:6024] |
| LIF | Up-regulated | protein_coding | atonal homolog 8 (Drosophila) [Source:HGNC Symbol;Acc:24126] |
| CPA4 | Down-regulated | protein_coding | PX domain containing 1 [Source:HGNC Symbol;Acc:21361] |
| DOCK4 | Up-regulated | protein_coding | meningioma (disrupted in balanced translocation) 1 [Source:HGNC Symbol;Acc:7180] |
| PODXL | Down-regulated | protein_coding | RAB3B, member RAS oncogene family [Source:HGNC Symbol;Acc:9778] |
| DNAJB9 | Up-regulated | protein_coding | sodium channel, voltage-gated, type IX, alpha subunit [Source:HGNC Symbol;Acc:10597] |
| FLNC | Down-regulated | protein_coding | pleckstrin homology domain containing, family A (phosphoinositide binding specific) member 2 [Source:HGNC Symbol;Acc:14336] |
| SMO | Up-regulated | protein_coding | solute carrier family 38, member 11 [Source:HGNC Symbol;Acc:26836] |
| CHN1 | Up-regulated | protein_coding | BUB1 mitotic checkpoint serine/threonine kinase [Source:HGNC Symbol;Acc:1148] |
| ARHGAP22 | Up-regulated | protein_coding | transmembrane 4 L six family member 1 [Source:HGNC Symbol;Acc:11853] |
| TMOD2 | Down-regulated | protein_coding | activated leukocyte cell adhesion molecule [Source:HGNC Symbol;Acc:400] |
| ALDH1A2 | Up-regulated | protein_coding | trafficking protein particle complex 1 [Source:HGNC Symbol;Acc:19894] |
| FAM63B | Down-regulated | protein_coding | ring finger protein 150 [Source:HGNC Symbol;Acc:23138] |
| CCRL1 | Down-regulated | protein_coding | doublecortin-like kinase 2 [Source:HGNC Symbol;Acc:19002] |
| PALLD | Down-regulated | protein_coding | cadherin 2, type 1, N-cadherin (neuronal) [Source:HGNC Symbol;Acc:1759] |
| FAM64A | Down-regulated | protein_coding | tetratricopeptide repeat, ankyrin repeat and coiled-coil containing 2 [Source:HGNC Symbol;Acc:30212] |
| SLC44A2 | Down-regulated | protein_coding | fasciculation and elongation protein zeta 2 (zygin II) [Source:HGNC Symbol;Acc:3660] |
| MTUS1 | Up-regulated | protein_coding | SHC SH2-domain binding protein 1 [Source:HGNC Symbol;Acc:29547] |
| AJUBA | Down-regulated | protein_coding | keratin 19 [Source:HGNC Symbol;Acc:6436] |
| SAT1 | Up-regulated | protein_coding | potassium voltage-gated channel, Shal-related subfamily, member 3 [Source:HGNC Symbol;Acc:6239] |
| LDLR | Down-regulated | protein_coding | collagen, type VIII, alpha 2 [Source:HGNC Symbol;Acc:2216] |
| CNN1 | Down-regulated | protein_coding | ribonucleotide reductase M2 [Source:HGNC Symbol;Acc:10452] |
| APOE | Up-regulated | protein_coding | leucine rich repeat containing 15 [Source:HGNC Symbol;Acc:20818] |
| ACTN4 | Down-regulated | protein_coding | cold shock domain containing C2, RNA binding [Source:HGNC Symbol;Acc:30359] |
| GDF15 | Up-regulated | protein_coding | regulator of calcineurin 2 [Source:HGNC Symbol;Acc:3041] |
| H19 | Down-regulated | processed_transcript | synaptopodin 2 [Source:HGNC Symbol;Acc:17732] |
| LAMA5 | Up-regulated | protein_coding | cofilin 1 (non-muscle) [Source:HGNC Symbol;Acc:1874] |
| ASS1 | Up-regulated | protein_coding | 7-dehydrocholesterol reductase [Source:HGNC Symbol;Acc:2860] |
| NPAS1 | Down-regulated | protein_coding | - |
| THEMIS2 | Up-regulated | protein_coding | Homo sapiens NKF3 kinase family member (PEAK1), mRNA. [Source:RefSeq mRNA;Acc:NM_024776] |
| ZNF331 | Up-regulated | protein_coding | nucleic acid binding protein 1 [Source:HGNC Symbol;Acc:26232] |
| AKAP12 | Down-regulated | protein_coding | heat shock 27kDa protein family, member 7 (cardiovascular) [Source:HGNC Symbol;Acc:5249] |
| SYNE1 | Down-regulated | protein_coding | heart development protein with EGF-like domains 1 [Source:HGNC Symbol;Acc:29227] |
| CAP1 | Down-regulated | protein_coding | methionine sulfoxide reductase B3 [Source:HGNC Symbol;Acc:27375] |
| SH3BP5 | Up-regulated | protein_coding | RGM domain family, member B [Source:HGNC Symbol;Acc:26896] |
| GALNT15 | Up-regulated | protein_coding | podocan [Source:HGNC Symbol;Acc:23174] |
| PDLIM4 | Up-regulated | protein_coding | tumor protein p53 inducible protein 11 [Source:HGNC Symbol;Acc:16842] |
| GFPT2 | Up-regulated | protein_coding | clathrin, light chain B [Source:HGNC Symbol;Acc:2091] |
| DIAPH1 | Down-regulated | protein_coding | target of myb1-like 2 (chicken) [Source:HGNC Symbol;Acc:11984] |
| ANO1 | Up-regulated | protein_coding | tubulin, beta 6 class V [Source:HGNC Symbol;Acc:20776] |
| NINJ1 | Up-regulated | protein_coding | lamin B2 [Source:HGNC Symbol;Acc:6638] |
| MAP1B | Down-regulated | protein_coding | BCL2-related ovarian killer [Source:HGNC Symbol;Acc:1087] |
| KRT34 | Down-regulated | protein_coding | brain abundant, membrane attached signal protein 1 [Source:HGNC Symbol;Acc:957] |
| CHSY1 | Up-regulated | protein_coding | thymidylate synthetase [Source:HGNC Symbol;Acc:12441] |
| GCH1 | Up-regulated | protein_coding | EPS8-like 2 [Source:HGNC Symbol;Acc:21296] |
| ENOSF1 | Up-regulated | protein_coding | Parkinson disease 7 domain containing 1 [Source:HGNC Symbol;Acc:26616] |
| PTPRE | Up-regulated | protein_coding | PRKC, apoptosis, WT1, regulator [Source:HGNC Symbol;Acc:8614] |
| SERPINF1 | Up-regulated | protein_coding | polymerase I and transcript release factor [Source:HGNC Symbol;Acc:9688] |
| KDM6B | Up-regulated | protein_coding | polymerase (RNA) II (DNA directed) polypeptide L, 7.6kDa [Source:HGNC Symbol;Acc:9199] |
| MATN2 | Down-regulated | protein_coding | phosphodiesterase 4D interacting protein [Source:HGNC Symbol;Acc:15580] |
| SNAP25 | Up-regulated | protein_coding | plectin [Source:HGNC Symbol;Acc:9069] |
| RIN2 | Up-regulated | protein_coding | frizzled family receptor 2 [Source:HGNC Symbol;Acc:4040] |
| KANK4 | Down-regulated | protein_coding | mab-21-like 1 (C. elegans) [Source:HGNC Symbol;Acc:6757] |
| MYH10 | Down-regulated | protein_coding | gremlin 2, DAN family BMP antagonist [Source:HGNC Symbol;Acc:17655] |
| MPRIP | Down-regulated | protein_coding | oxytocin receptor [Source:HGNC Symbol;Acc:8529] |
| CHI3L1 | Up-regulated | protein_coding | RAP2B, member of RAS oncogene family [Source:HGNC Symbol;Acc:9862] |
| POSTN | Down-regulated | protein_coding | chromosome 5 open reading frame 30 [Source:HGNC Symbol;Acc:25052] |
| TPT1 | Up-regulated | protein_coding | exostosin glycosyltransferase 1 [Source:HGNC Symbol;Acc:3512] |
| STARD13 | Up-regulated | protein_coding | Tyrosine-protein kinase SgK223 [Source:UniProtKB/Swiss-Prot;Acc:Q86YV5] |
| MYH11 | Down-regulated | protein_coding | calcium channel, voltage-dependent, beta 4 subunit [Source:HGNC Symbol;Acc:1404] |
| C1QTNF6 | Up-regulated | protein_coding | trafficking protein, kinesin binding 1 [Source:HGNC Symbol;Acc:29947] |
| BTG1 | Up-regulated | protein_coding | annexin A2 [Source:HGNC Symbol;Acc:537] |
| SWAP70 | Down-regulated | protein_coding | gap junction protein, gamma 1, 45kDa [Source:HGNC Symbol;Acc:4280] |
| AMPD3 | Up-regulated | protein_coding | solute carrier family 8 (sodium/calcium exchanger), member 1 [Source:HGNC Symbol;Acc:11068] |
| MICAL2 | Down-regulated | protein_coding | growth arrest-specific 6 [Source:HGNC Symbol;Acc:4168] |
| RRAS2 | Down-regulated | protein_coding | collagen and calcium binding EGF domains 1 [Source:HGNC Symbol;Acc:29426] |
| TMEM66 | Up-regulated | protein_coding | family with sequence similarity 101, member B [Source:HGNC Symbol;Acc:28705] |
| NUMB | Up-regulated | protein_coding | IQ motif containing GTPase activating protein 3 [Source:HGNC Symbol;Acc:20669] |
| CTIF | Down-regulated | protein_coding | actin, gamma 1 [Source:HGNC Symbol;Acc:144] |
| CCNB1 | Down-regulated | protein_coding | protein kinase D1 [Source:HGNC Symbol;Acc:9407] |
| IRAK2 | Up-regulated | protein_coding | colony stimulating factor 1 (macrophage) [Source:HGNC Symbol;Acc:2432] |
| BHLHE40 | Up-regulated | protein_coding | transmembrane protein 173 [Source:HGNC Symbol;Acc:27962] |
| MEIS2 | Down-regulated | protein_coding | mucin 1, cell surface associated [Source:HGNC Symbol;Acc:7508] |
| VAV3 | Up-regulated | protein_coding | AHNAK nucleoprotein 2 [Source:HGNC Symbol;Acc:20125] |
| PSRC1 | Down-regulated | protein_coding | phosphate cytidylyltransferase 2, ethanolamine [Source:HGNC Symbol;Acc:8756] |
| SORT1 | Down-regulated | protein_coding | B-cell CLL/lymphoma 9-like [Source:HGNC Symbol;Acc:23688] |
| PTGFRN | Up-regulated | protein_coding | kinesin family member 18B [Source:HGNC Symbol;Acc:27102] |
| NGF | Down-regulated | protein_coding | neurofibromin 2 (merlin) [Source:HGNC Symbol;Acc:7773] |
| TMEM106C | Down-regulated | protein_coding | microphthalmia-associated transcription factor [Source:HGNC Symbol;Acc:7105] |
| SLC38A2 | Down-regulated | protein_coding | glucosaminyl (N-acetyl) transferase 1, core 2 [Source:HGNC Symbol;Acc:4203] |
| ROCK2 | Down-regulated | protein_coding | formin binding protein 1 [Source:HGNC Symbol;Acc:17069] |
| SOX5 | Up-regulated | protein_coding | sterile alpha motif domain containing 11 [Source:HGNC Symbol;Acc:28706] |
| SPOCD1 | Down-regulated | protein_coding | platelet endothelial aggregation receptor 1 [Source:HGNC Symbol;Acc:33631] |
| PHC2 | Up-regulated | protein_coding | chromosome 6 open reading frame 132 [Source:HGNC Symbol;Acc:21288] |
| CDCA8 | Down-regulated | protein_coding | tubulin, beta 4B class IVb [Source:HGNC Symbol;Acc:20771] |
| FHOD3 | Up-regulated | protein_coding | chromosome 15 open reading frame 52 [Source:HGNC Symbol;Acc:33488] |
| FADS2 | Down-regulated | protein_coding | proline/arginine-rich end leucine-rich repeat protein [Source:HGNC Symbol;Acc:9357] |
| PDGFRA | Up-regulated | protein_coding | reelin [Source:HGNC Symbol;Acc:9957] |
| COL4A2 | Up-regulated | protein_coding | hematological and neurological expressed 1 [Source:HGNC Symbol;Acc:14569] |
| ANXA1 | Down-regulated | protein_coding | S100 calcium binding protein A4 [Source:HGNC Symbol;Acc:10494] |
| CTSL1 | Up-regulated | protein_coding | tubulin, beta class I [Source:HGNC Symbol;Acc:20778] |
| ADAM19 | Down-regulated | protein_coding | actin filament associated protein 1 [Source:HGNC Symbol;Acc:24017] |
| TBX3 | Up-regulated | protein_coding | calcium channel, voltage-dependent, T type, alpha 1H subunit [Source:HGNC Symbol;Acc:1395] |
| ITGA7 | Down-regulated | protein_coding | myosin VI [Source:HGNC Symbol;Acc:7605] |
| FAIM2 | Up-regulated | protein_coding | megakaryoblastic leukemia (translocation) 1 [Source:HGNC Symbol;Acc:14334] |
| KRT7 | Down-regulated | protein_coding | alcohol dehydrogenase 1B (class I), beta polypeptide [Source:HGNC Symbol;Acc:250] |
| MAP7 | Up-regulated | protein_coding | death-associated protein kinase 1 [Source:HGNC Symbol;Acc:2674] |
| MICAL1 | Down-regulated | protein_coding | sodium channel, voltage gated, type VIII, alpha subunit [Source:HGNC Symbol;Acc:10596] |
| DOCK10 | Down-regulated | protein_coding | PDZ and LIM domain 7 (enigma) [Source:HGNC Symbol;Acc:22958] |
| SERPINE2 | Up-regulated | protein_coding | filamin A, alpha [Source:HGNC Symbol;Acc:3754] |
| PLXNC1 | Down-regulated | protein_coding | SLIT-ROBO Rho GTPase activating protein 1 [Source:HGNC Symbol;Acc:17382] |
| APPL2 | Down-regulated | protein_coding | annexin A6 [Source:HGNC Symbol;Acc:544] |
| DRAM1 | Down-regulated | protein_coding | microfibrillar associated protein 5 [Source:HGNC Symbol;Acc:29673] |
| FLNB | Down-regulated | protein_coding | small glutamine-rich tetratricopeptide repeat (TPR)-containing, beta [Source:HGNC Symbol;Acc:23567] |
| CKAP2 | Down-regulated | protein_coding | myosin IC [Source:HGNC Symbol;Acc:7597] |
| LRCH1 | Up-regulated | protein_coding | FCH and double SH3 domains 1 [Source:HGNC Symbol;Acc:25463] |
| LMO7 | Down-regulated | protein_coding | WW domain containing E3 ubiquitin protein ligase 2 [Source:HGNC Symbol;Acc:16804] |
| ITM2B | Up-regulated | protein_coding | tropomyosin 2 (beta) [Source:HGNC Symbol;Acc:12011] |
| SPRY2 | Up-regulated | protein_coding | integrin, beta-like 1 (with EGF-like repeat domains) [Source:HGNC Symbol;Acc:6164] |
| EDNRB | Up-regulated | protein_coding | 3'-phosphoadenosine 5'-phosphosulfate synthase 2 [Source:HGNC Symbol;Acc:8604] |
| LCP1 | Up-regulated | protein_coding | alpha-kinase 2 [Source:HGNC Symbol;Acc:20565] |
| TNS3 | Down-regulated | protein_coding | Rho GTPase activating protein 11A [Source:HGNC Symbol;Acc:15783] |
| GPNMB | Up-regulated | protein_coding | protein regulator of cytokinesis 1 [Source:HGNC Symbol;Acc:9341] |
| IL6 | Up-regulated | protein_coding | L1 cell adhesion molecule [Source:HGNC Symbol;Acc:6470] |
| ADAMTS7 | Down-regulated | protein_coding | sterol regulatory element binding transcription factor 2 [Source:HGNC Symbol;Acc:11290] |
| ABHD17C | Up-regulated | protein_coding | layilin [Source:HGNC Symbol;Acc:29471] |
| IL1RN | Up-regulated | protein_coding | pregnancy specific beta-1-glycoprotein 5 [Source:HGNC Symbol;Acc:9522] |
| GYPC | Up-regulated | protein_coding | leucine-rich repeat containing G protein-coupled receptor 4 [Source:HGNC Symbol;Acc:13299] |
| DNAJC1 | Up-regulated | protein_coding | tectonin beta-propeller repeat containing 1 [Source:HGNC Symbol;Acc:22214] |
| NIPSNAP3A | Down-regulated | protein_coding | ATPase, class V, type 10A [Source:HGNC Symbol;Acc:13542] |
| ANGPTL2 | Up-regulated | protein_coding | protein tyrosine phosphatase-like (proline instead of catalytic arginine), member b [Source:HGNC Symbol;Acc:9640] |
| ATP6V1G1 | Up-regulated | protein_coding | vestigial like 3 (Drosophila) [Source:HGNC Symbol;Acc:24327] |
| ENPP2 | Down-regulated | protein_coding | limb bud and heart development [Source:HGNC Symbol;Acc:29532] |
| IL33 | Up-regulated | protein_coding | integrin, alpha 1 [Source:HGNC Symbol;Acc:6134] |
| TLN1 | Down-regulated | protein_coding | keratin associated protein 1-5 [Source:HGNC Symbol;Acc:16777] |
| SLC22A23 | Up-regulated | protein_coding | long intergenic non-protein coding RNA 152 [Source:HGNC Symbol;Acc:28717] |
| TCF19 | Down-regulated | protein_coding | - |
| FLOT1 | Down-regulated | protein_coding | Rho GTPase activating protein 23 [Source:HGNC Symbol;Acc:29293] |
| IER3 | Up-regulated | protein_coding | jerky homolog (mouse) [Source:HGNC Symbol;Acc:6199] |
| SYTL2 | Down-regulated | protein_coding | chromosome 12 open reading frame 75 [Source:HGNC Symbol;Acc:35164] |
| LRRC32 | Up-regulated | protein_coding | TRHDE antisense RNA 1 [Source:HGNC Symbol;Acc:27471] |
| PI15 | Up-regulated | protein_coding | brain cytoplasmic RNA 1 [Source:HGNC Symbol;Acc:1022] |
| SULF1 | Down-regulated | protein_coding | kinesin family member C1 [Source:HGNC Symbol;Acc:6389] |
| TRPC6 | Down-regulated | protein_coding | chromosome 8 open reading frame 58 [Source:HGNC Symbol;Acc:32233] |
| SQRDL | Up-regulated | protein_coding | paternally expressed 10 [Source:HGNC Symbol;Acc:14005] |
| THBS1 | Down-regulated | protein_coding | microtubule associated monooxygenase, calponin and LIM domain containing 3 [Source:HGNC Symbol;Acc:24694] |
| KIF23 | Down-regulated | protein_coding | - |
| ITGA11 | Down-regulated | protein_coding | STARD4 antisense RNA 1 [Source:HGNC Symbol;Acc:44117] |
| CASC5 | Down-regulated | protein_coding | MIR143 host gene (non-protein coding) [Source:HGNC Symbol;Acc:42872] |
| UACA | Down-regulated | protein_coding | KIAA1456 [Source:HGNC Symbol;Acc:26725] |
| SMAD6 | Down-regulated | protein_coding | TMF1-regulated nuclear protein 1 [Source:HGNC Symbol;Acc:34348] |
| STRA6 | Up-regulated | protein_coding | - |
| BCAR3 | Down-regulated | protein_coding | - |
| ARHGAP29 | Down-regulated | protein_coding | KCNQ1 opposite strand/antisense transcript 1 (non-protein coding) [Source:HGNC Symbol;Acc:6295] |
| MYOF | Down-regulated | protein_coding | hsa-mir-145 [Source:miRBase;Acc:MI0000461] |
| LOXL4 | Down-regulated | protein_coding | Description |
| CH25H | Up-regulated | protein_coding | NIPA-like domain containing 3 [Source:HGNC Symbol;Acc:25233] |
| KIF11 | Down-regulated | protein_coding | LIM and SH3 protein 1 [Source:HGNC Symbol;Acc:6513] |
| DUSP5 | Up-regulated | protein_coding | heat shock protein, alpha-crystallin-related, B6 [Source:HGNC Symbol;Acc:26511] |
| CEP55 | Down-regulated | protein_coding | coatomer protein complex, subunit zeta 2 [Source:HGNC Symbol;Acc:19356] |
| PLCE1 | Down-regulated | protein_coding | integrin, alpha 3 (antigen CD49C, alpha 3 subunit of VLA-3 receptor) [Source:HGNC Symbol;Acc:6139] |
| MYPN | Down-regulated | protein_coding | aldehyde dehydrogenase 3 family, member B1 [Source:HGNC Symbol;Acc:410] |
| NAB1 | Up-regulated | protein_coding | cytohesin 3 [Source:HGNC Symbol;Acc:9504] |
| CILP | Up-regulated | protein_coding | nuclear factor I/X (CCAAT-binding transcription factor) [Source:HGNC Symbol;Acc:7788] |
| HERC3 | Down-regulated | protein_coding | anillin, actin binding protein [Source:HGNC Symbol;Acc:14082] |
| FGF5 | Down-regulated | protein_coding | RAB GTPase activating protein 1 [Source:HGNC Symbol;Acc:17155] |
| AGPAT9 | Up-regulated | protein_coding | sema domain, immunoglobulin domain (Ig), short basic domain, secreted, (semaphorin) 3B [Source:HGNC Symbol;Acc:10724] |
| PDE5A | Down-regulated | protein_coding | EH-domain containing 3 [Source:HGNC Symbol;Acc:3244] |
| 11-Sep | Down-regulated | protein_coding | claudin 11 [Source:HGNC Symbol;Acc:8514] |
| SHROOM3 | Down-regulated | protein_coding | G protein-coupled receptor, family C, group 5, member A [Source:HGNC Symbol;Acc:9836] |
| SLC39A8 | Up-regulated | protein_coding | transforming, acidic coiled-coil containing protein 3 [Source:HGNC Symbol;Acc:11524] |
| FBN2 | Down-regulated | protein_coding | ralA binding protein 1 [Source:HGNC Symbol;Acc:9841] |
| RGS3 | Up-regulated | protein_coding | four and a half LIM domains 1 [Source:HGNC Symbol;Acc:3702] |
| AMIGO2 | Down-regulated | protein_coding | EH-domain containing 2 [Source:HGNC Symbol;Acc:3243] |
| GLIPR1 | Down-regulated | protein_coding | vimentin [Source:HGNC Symbol;Acc:12692] |
| PHLDA1 | Up-regulated | protein_coding | ubiquitin-like with PHD and ring finger domains 1 [Source:HGNC Symbol;Acc:12556] |
| LGR5 | Down-regulated | protein_coding | vinculin [Source:HGNC Symbol;Acc:12665] |
| DUSP6 | Up-regulated | protein_coding | death-associated protein kinase 2 [Source:HGNC Symbol;Acc:2675] |
| NEDD1 | Down-regulated | protein_coding | capping protein (actin filament), gelsolin-like [Source:HGNC Symbol;Acc:1474] |
| TMEM132B | Up-regulated | protein_coding | phosphatidylinositol-3,4,5-trisphosphate-dependent Rac exchange factor 2 [Source:HGNC Symbol;Acc:22950] |
| SLC7A1 | Down-regulated | protein_coding | KIT ligand [Source:HGNC Symbol;Acc:6343] |
| ESYT1 | Down-regulated | protein_coding | steroid-5-alpha-reductase, alpha polypeptide 2 (3-oxo-5 alpha-steroid delta 4-dehydrogenase alpha 2) [Source:HGNC Symbol;Acc:11285] |
| DIAPH3 | Down-regulated | protein_coding | elastin [Source:HGNC Symbol;Acc:3327] |
| MBNL2 | Down-regulated | protein_coding | dickkopf 3 homolog (Xenopus laevis) [Source:HGNC Symbol;Acc:2893] |
| SSTR1 | Down-regulated | protein_coding | LIM domain and actin binding 1 [Source:HGNC Symbol;Acc:24636] |
| FRMD6 | Down-regulated | protein_coding | spectrin repeat containing, nuclear envelope 2 [Source:HGNC Symbol;Acc:17084] |
| FBLN5 | Down-regulated | protein_coding | coiled-coil domain containing 85A [Source:HGNC Symbol;Acc:29400] |
| FGF7 | Up-regulated | protein_coding | protein phosphatase 1, regulatory subunit 12A [Source:HGNC Symbol;Acc:7618] |
| MESDC1 | Up-regulated | protein_coding | ATPase, Ca++ transporting, plasma membrane 4 [Source:HGNC Symbol;Acc:817] |
| TPM1 | Down-regulated | protein_coding | collagen, type XI, alpha 1 [Source:HGNC Symbol;Acc:2186] |
| ARRDC4 | Down-regulated | protein_coding | aryl-hydrocarbon receptor repressor [Source:HGNC Symbol;Acc:346] |
| PCSK6 | Up-regulated | protein_coding | LIM and calponin homology domains 1 [Source:HGNC Symbol;Acc:29191] |
| TGFB1I1 | Down-regulated | protein_coding | calponin 2 [Source:HGNC Symbol;Acc:2156] |
| ARHGAP17 | Down-regulated | protein_coding | solute carrier family 9, subfamily A (NHE3, cation proton antiporter 3), member 3 regulator 2 [Source:HGNC Symbol;Acc:11076] |
| NLRC5 | Down-regulated | protein_coding | importin 5 [Source:HGNC Symbol;Acc:6402] |
| TLDC1 | Down-regulated | protein_coding | myosin light chain kinase [Source:HGNC Symbol;Acc:7590] |
| MYOCD | Down-regulated | protein_coding | asp (abnormal spindle) homolog, microcephaly associated (Drosophila) [Source:HGNC Symbol;Acc:19048] |
| ABCA8 | Up-regulated | protein_coding | neuron navigator 3 [Source:HGNC Symbol;Acc:15998] |
| SECTM1 | Up-regulated | protein_coding | tetratricopeptide repeat domain 7A [Source:HGNC Symbol;Acc:19750] |
| PMAIP1 | Up-regulated | protein_coding | follistatin-like 3 (secreted glycoprotein) [Source:HGNC Symbol;Acc:3973] |
| ERBB2 | Down-regulated | protein_coding | protein tyrosine phosphatase, non-receptor type 21 [Source:HGNC Symbol;Acc:9651] |
| COL6A1 | Up-regulated | protein_coding | WD repeat domain 1 [Source:HGNC Symbol;Acc:12754] |
| COL6A2 | Up-regulated | protein_coding | ribosomal protein S6 kinase, 90kDa, polypeptide 2 [Source:HGNC Symbol;Acc:10431] |
| SIK1 | Up-regulated | protein_coding | LIM and cysteine-rich domains 1 [Source:HGNC Symbol;Acc:6633] |
| DOPEY2 | Down-regulated | protein_coding | thyroid hormone receptor interactor 13 [Source:HGNC Symbol;Acc:12307] |
| WTIP | Down-regulated | protein_coding | actinin, alpha 1 [Source:HGNC Symbol;Acc:163] |
| RPS11 | Up-regulated | protein_coding | LIM and senescent cell antigen-like domains 2 [Source:HGNC Symbol;Acc:16084] |
| RPL13A | Up-regulated | protein_coding | SPEG complex locus [Source:HGNC Symbol;Acc:16901] |
| EPHA2 | Down-regulated | protein_coding | thyrotropin-releasing hormone degrading enzyme [Source:HGNC Symbol;Acc:30748] |
| PGD | Up-regulated | protein_coding | murine retrovirus integration site 1 homolog [Source:HGNC Symbol;Acc:7237] |
| SH3BGRL3 | Down-regulated | protein_coding | fermitin family member 2 [Source:HGNC Symbol;Acc:15767] |
| HSPG2 | Down-regulated | protein_coding | furry homolog (Drosophila) [Source:HGNC Symbol;Acc:20367] |
| CYR61 | Down-regulated | protein_coding | monoglyceride lipase [Source:HGNC Symbol;Acc:17038] |
| TINAGL1 | Down-regulated | protein_coding | netrin 4 [Source:HGNC Symbol;Acc:13658] |
| PTPRF | Down-regulated | protein_coding | NUAK family, SNF1-like kinase, 1 [Source:HGNC Symbol;Acc:14311] |
| LMO4 | Up-regulated | protein_coding | G-2 and S-phase expressed 1 [Source:HGNC Symbol;Acc:13698] |
| CREG1 | Up-regulated | protein_coding | sema domain, immunoglobulin domain (Ig), short basic domain, secreted, (semaphorin) 3C [Source:HGNC Symbol;Acc:10725] |
| DPT | Up-regulated | protein_coding | RAS protein activator like 2 [Source:HGNC Symbol;Acc:9874] |
| RGS5 | Up-regulated | protein_coding | actin, beta [Source:HGNC Symbol;Acc:132] |
| RGS16 | Up-regulated | protein_coding | RNA binding motif, single stranded interacting protein 2 [Source:HGNC Symbol;Acc:9909] |
| ECM1 | Up-regulated | protein_coding | ankyrin repeat domain 13A [Source:HGNC Symbol;Acc:21268] |
| MCL1 | Up-regulated | protein_coding | protein phosphatase 1, regulatory subunit 12B [Source:HGNC Symbol;Acc:7619] |
| CTSK | Up-regulated | protein_coding | PHD finger protein 17 [Source:HGNC Symbol;Acc:30027] |
| DUSP10 | Up-regulated | protein_coding | monooxygenase, DBH-like 1 [Source:HGNC Symbol;Acc:21063] |
| RAB13 | Up-regulated | protein_coding | ATPase, aminophospholipid transporter, class I, type 8B, member 1 [Source:HGNC Symbol;Acc:3706] |
| CNIH3 | Up-regulated | protein_coding | cordon-bleu WH2 repeat protein-like 1 [Source:HGNC Symbol;Acc:23571] |
| EPHX1 | Up-regulated | protein_coding | potassium channel, subfamily K, member 2 [Source:HGNC Symbol;Acc:6277] |
| CALM2 | Down-regulated | protein_coding | ADAM metallopeptidase with thrombospondin type 1 motif, 2 [Source:HGNC Symbol;Acc:218] |
| SLC20A1 | Up-regulated | protein_coding | aurora kinase A [Source:HGNC Symbol;Acc:11393] |
| AFF3 | Down-regulated | protein_coding | TPX2, microtubule-associated, homolog (Xenopus laevis) [Source:HGNC Symbol;Acc:1249] |
| CXCR7 | Up-regulated | protein_coding | paxillin [Source:HGNC Symbol;Acc:9718] |
| VGLL4 | Up-regulated | protein_coding | baculoviral IAP repeat containing 5 [Source:HGNC Symbol;Acc:593] |
| 4-Mar | Down-regulated | protein_coding | poly(rC) binding protein 4 [Source:HGNC Symbol;Acc:8652] |
| CSRNP1 | Up-regulated | protein_coding | integrin, alpha 6 [Source:HGNC Symbol;Acc:6142] |
| LRIG1 | Up-regulated | protein_coding | zinc finger homeobox 4 [Source:HGNC Symbol;Acc:30939] |
| LIMD1 | Up-regulated | protein_coding | coiled-coil domain containing 80 [Source:HGNC Symbol;Acc:30649] |
| NFKBIZ | Up-regulated | protein_coding | ezrin [Source:HGNC Symbol;Acc:12691] |
| PHLDB2 | Down-regulated | protein_coding | myosin, light chain 6, alkali, smooth muscle and non-muscle [Source:HGNC Symbol;Acc:7587] |
| NCEH1 | Down-regulated | protein_coding | TBC1 domain family, member 2 [Source:HGNC Symbol;Acc:18026] |
| KIAA0226 | Down-regulated | protein_coding | desmoplakin [Source:HGNC Symbol;Acc:3052] |
| TCTA | Down-regulated | protein_coding | stearoyl-CoA desaturase (delta-9-desaturase) [Source:HGNC Symbol;Acc:10571] |
| UCN2 | Up-regulated | protein_coding | actin binding LIM protein 1 [Source:HGNC Symbol;Acc:78] |
| SLIT2 | Down-regulated | protein_coding | paralemmin [Source:HGNC Symbol;Acc:8594] |
| EPHA5 | Down-regulated | protein_coding | mediator complex subunit 15 [Source:HGNC Symbol;Acc:14248] |
| SNCA | Up-regulated | protein_coding | MICAL-like 1 [Source:HGNC Symbol;Acc:29804] |
| CCNA2 | Down-regulated | protein_coding | Sad1 and UNC84 domain containing 2 [Source:HGNC Symbol;Acc:14210] |
| USP53 | Down-regulated | protein_coding | minichromosome maintenance complex component 5 [Source:HGNC Symbol;Acc:6948] |
| SFRP2 | Up-regulated | protein_coding | myosin, heavy chain 9, non-muscle [Source:HGNC Symbol;Acc:7579] |
| NKD2 | Up-regulated | protein_coding | Ran GTPase activating protein 1 [Source:HGNC Symbol;Acc:9854] |
| MYO10 | Down-regulated | protein_coding | phosphorylase, glycogen, liver [Source:HGNC Symbol;Acc:9725] |
| SKP2 | Down-regulated | protein_coding | serine palmitoyltransferase, long chain base subunit 2 [Source:HGNC Symbol;Acc:11278] |
| PLK2 | Up-regulated | protein_coding | Ras and Rab interactor 3 [Source:HGNC Symbol;Acc:18751] |
| LHFPL2 | Up-regulated | protein_coding | legumain [Source:HGNC Symbol;Acc:9472] |
| TSLP | Up-regulated | protein_coding | phosphoenolpyruvate carboxykinase 2 (mitochondrial) [Source:HGNC Symbol;Acc:8725] |
| TNFAIP8 | Up-regulated | protein_coding | v-myb myeloblastosis viral oncogene homolog (avian)-like 2 [Source:HGNC Symbol;Acc:7548] |
| MEGF10 | Up-regulated | protein_coding | myosin, light chain 9, regulatory [Source:HGNC Symbol;Acc:15754] |
| TENM2 | Down-regulated | protein_coding | jagged 1 [Source:HGNC Symbol;Acc:6188] |
| TNFRSF21 | Up-regulated | protein_coding | myosin, light chain 12A, regulatory, non-sarcomeric [Source:HGNC Symbol;Acc:16701] |
| DAAM2 | Down-regulated | protein_coding | matrix-remodelling associated 5 [Source:HGNC Symbol;Acc:7539] |
| SCUBE3 | Down-regulated | protein_coding | plastin 3 [Source:HGNC Symbol;Acc:9091] |
| PNRC1 | Up-regulated | protein_coding | early endosome antigen 1 [Source:HGNC Symbol;Acc:3185] |
| ARHGAP18 | Down-regulated | protein_coding | fms-related tyrosine kinase 1 [Source:HGNC Symbol;Acc:3763] |
| WTAP | Up-regulated | protein_coding | katanin p60 subunit A-like 1 [Source:HGNC Symbol;Acc:28361] |
| SDK1 | Up-regulated | protein_coding | host cell factor C1 regulator 1 (XPO1 dependent) [Source:HGNC Symbol;Acc:21198] |
| CREB5 | Up-regulated | protein_coding | WAP four-disulfide core domain 1 [Source:HGNC Symbol;Acc:15466] |
| IGFBP3 | Down-regulated | protein_coding | coactosin-like 1 (Dictyostelium) [Source:HGNC Symbol;Acc:18304] |
| NCAPG2 | Down-regulated | protein_coding | piezo-type mechanosensitive ion channel component 1 [Source:HGNC Symbol;Acc:28993] |
| DENND2A | Up-regulated | protein_coding | calcineurin-like phosphoesterase domain containing 1 [Source:HGNC Symbol;Acc:25632] |
| TMEM47 | Down-regulated | protein_coding | EH-domain containing 4 [Source:HGNC Symbol;Acc:3245] |
| SLC16A2 | Down-regulated | protein_coding | zinc finger, DHHC-type containing 2 [Source:HGNC Symbol;Acc:18469] |
| PRPS1 | Down-regulated | protein_coding | stathmin-like 2 [Source:HGNC Symbol;Acc:10577] |
| CSGALNACT1 | Up-regulated | protein_coding | SH2 domain containing 4A [Source:HGNC Symbol;Acc:26102] |
| PLIN2 | Up-regulated | protein_coding | Rho guanine nucleotide exchange factor (GEF) 10 [Source:HGNC Symbol;Acc:14103] |
| CDKN2B | Up-regulated | protein_coding | protein phosphatase 1, regulatory subunit 13 like [Source:HGNC Symbol;Acc:18838] |
| C9orf3 | Down-regulated | protein_coding | excision repair cross-complementing rodent repair deficiency, complementation group 2 [Source:HGNC Symbol;Acc:3434] |
| STOM | Down-regulated | protein_coding | cyclin-dependent kinase 6 [Source:HGNC Symbol;Acc:1777] |
| PTGES | Up-regulated | protein_coding | caveolin 2 [Source:HGNC Symbol;Acc:1528] |
| RSU1 | Down-regulated | protein_coding | caveolin 1, caveolae protein, 22kDa [Source:HGNC Symbol;Acc:1527] |
| HERC4 | Down-regulated | protein_coding | met proto-oncogene (hepatocyte growth factor receptor) [Source:HGNC Symbol;Acc:7029] |
| CAMK2G | Down-regulated | protein_coding | wingless-type MMTV integration site family member 2 [Source:HGNC Symbol;Acc:12780] |
| ANKRD1 | Down-regulated | protein_coding | homeobox A13 [Source:HGNC Symbol;Acc:5102] |
| MKI67 | Down-regulated | protein_coding | heat shock 27kDa protein 1 [Source:HGNC Symbol;Acc:5246] |
| ITPRIP | Up-regulated | protein_coding | serpin peptidase inhibitor, clade E (nexin, plasminogen activator inhibitor type 1), member 1 [Source:HGNC Symbol;Acc:8583] |
| ADAM12 | Up-regulated | protein_coding | mesoderm specific transcript [Source:HGNC Symbol;Acc:7028] |
| ENDOD1 | Down-regulated | protein_coding | mesenchyme homeobox 2 [Source:HGNC Symbol;Acc:7014] |
| ZC3H12C | Up-regulated | protein_coding | prune homolog 2 (Drosophila) [Source:HGNC Symbol;Acc:25209] |
| P4HA3 | Up-regulated | protein_coding | asporin [Source:HGNC Symbol;Acc:14872] |
| ADAM33 | Down-regulated | protein_coding | KN motif and ankyrin repeat domains 1 [Source:HGNC Symbol;Acc:19309] |
| KIRREL3 | Down-regulated | protein_coding | PDZ and LIM domain 1 [Source:HGNC Symbol;Acc:2067] |
| TAGLN | Down-regulated | protein_coding | chemokine (C-X-C motif) ligand 12 [Source:HGNC Symbol;Acc:10672] |
| JPH2 | Down-regulated | protein_coding | chromosome 10 open reading frame 54 [Source:HGNC Symbol;Acc:30085] |
| KIAA1755 | Down-regulated | protein_coding | actin, alpha 2, smooth muscle, aorta [Source:HGNC Symbol;Acc:130] |
| PLCB3 | Down-regulated | protein_coding | dickkopf 1 homolog (Xenopus laevis) [Source:HGNC Symbol;Acc:2891] |
| MKX | Down-regulated | protein_coding | vesicle amine transport protein 1 homolog (T. californica) [Source:HGNC Symbol;Acc:16919] |
| ADRA2A | Down-regulated | protein_coding | SMAD specific E3 ubiquitin protein ligase 2 [Source:HGNC Symbol;Acc:16809] |
| VEGFC | Down-regulated | protein_coding | inositol polyphosphate-4-phosphatase, type II, 105kDa [Source:HGNC Symbol;Acc:6075] |
| PRSS23 | Down-regulated | protein_coding | Wolf-Hirschhorn syndrome candidate 1 [Source:HGNC Symbol;Acc:12766] |
| FOXO1 | Up-regulated | protein_coding | non-SMC condensin I complex, subunit G [Source:HGNC Symbol;Acc:24304] |
| CRIM1 | Down-regulated | protein_coding | crystallin, alpha B [Source:HGNC Symbol;Acc:2389] |
| ITPR1 | Up-regulated | protein_coding | Rho guanine nucleotide exchange factor (GEF) 17 [Source:HGNC Symbol;Acc:21726] |
| CACNA1C | Down-regulated | protein_coding | coronin, actin binding protein, 1C [Source:HGNC Symbol;Acc:2254] |
| SLC2A13 | Up-regulated | protein_coding | keratin 18 [Source:HGNC Symbol;Acc:6430] |
| MAGI1 | Down-regulated | protein_coding | forkhead box M1 [Source:HGNC Symbol;Acc:3818] |
| ADAMTS12 | Up-regulated | protein_coding | solute carrier family 38, member 1 [Source:HGNC Symbol;Acc:13447] |
| NEK7 | Down-regulated | protein_coding | G protein-coupled receptor 133 [Source:HGNC Symbol;Acc:19893] |
| EPS8 | Down-regulated | protein_coding | collagen, type XII, alpha 1 [Source:HGNC Symbol;Acc:2188] |
| EDNRA | Up-regulated | protein_coding | neural precursor cell expressed, developmentally down-regulated 9 [Source:HGNC Symbol;Acc:7733] |
| AKR1C2 | Up-regulated | protein_coding | serine/threonine kinase 38 [Source:HGNC Symbol;Acc:17847] |
| DPYSL4 | Up-regulated | protein_coding | CAP, adenylate cyclase-associated protein, 2 (yeast) [Source:HGNC Symbol;Acc:20039] |
| RNF144A | Up-regulated | protein_coding | single-minded homolog 1 (Drosophila) [Source:HGNC Symbol;Acc:10882] |
| WWC2 | Down-regulated | protein_coding | G protein-coupled receptor 126 [Source:HGNC Symbol;Acc:13841] |
| SLC25A4 | Down-regulated | protein_coding | phosphatase and actin regulator 2 [Source:HGNC Symbol;Acc:20956] |
| DST | Down-regulated | protein_coding | erbb2 interacting protein [Source:HGNC Symbol;Acc:15842] |
| PTPN14 | Down-regulated | protein_coding | 3-hydroxy-3-methylglutaryl-CoA synthase 1 (soluble) [Source:HGNC Symbol;Acc:5007] |
| SETBP1 | Up-regulated | protein_coding | kinesin family member 20A [Source:HGNC Symbol;Acc:9787] |
| EPG5 | Down-regulated | protein_coding | lysyl oxidase [Source:HGNC Symbol;Acc:6664] |
| SPON1 | Up-regulated | processed_transcript | phosphodiesterase 8B [Source:HGNC Symbol;Acc:8794] |
| PDE3B | Up-regulated | protein_coding | cadherin 6, type 2, K-cadherin (fetal kidney) [Source:HGNC Symbol;Acc:1765] |
| MBNL1 | Down-regulated | protein_coding | lamin B1 [Source:HGNC Symbol;Acc:6637] |
| GJA1 | Up-regulated | protein_coding | natriuretic peptide receptor C/guanylate cyclase C (atrionatriuretic peptide receptor C) [Source:HGNC Symbol;Acc:7945] |
| FARP1 | Down-regulated | protein_coding | fibroblast growth factor 1 (acidic) [Source:HGNC Symbol;Acc:3665] |
| UTRN | Down-regulated | protein_coding | stanniocalcin 2 [Source:HGNC Symbol;Acc:11374] |
| GPR125 | Up-regulated | protein_coding | angiomotin like 2 [Source:HGNC Symbol;Acc:17812] |
| DAB2 | Down-regulated | protein_coding | hairy and enhancer of split 1, (Drosophila) [Source:HGNC Symbol;Acc:5192] |
| BCL2L11 | Up-regulated | protein_coding | epithelial cell transforming sequence 2 oncogene [Source:HGNC Symbol;Acc:3155] |
| BMP6 | Up-regulated | protein_coding | ARP3 actin-related protein 3 homolog (yeast) [Source:HGNC Symbol;Acc:170] |
| NR4A2 | Up-regulated | protein_coding | tumor protein p53 inducible protein 3 [Source:HGNC Symbol;Acc:19373] |
| ING1 | Up-regulated | protein_coding | integrin, alpha 4 (antigen CD49D, alpha 4 subunit of VLA-4 receptor) [Source:HGNC Symbol;Acc:6140] |
| RMND5A | Up-regulated | protein_coding | reticulon 4 [Source:HGNC Symbol;Acc:14085] |
| PTPRD | Up-regulated | protein_coding | EGF containing fibulin-like extracellular matrix protein 1 [Source:HGNC Symbol;Acc:3218] |
| SPHKAP | Up-regulated | protein_coding | glutaminase [Source:HGNC Symbol;Acc:4331] |
| PID1 | Up-regulated | protein_coding | EF-hand domain family, member D1 [Source:HGNC Symbol;Acc:29556] |
| DDAH1 | Down-regulated | protein_coding | EH domain binding protein 1 [Source:HGNC Symbol;Acc:29144] |
| HS2ST1 | Up-regulated | protein_coding | four and a half LIM domains 2 [Source:HGNC Symbol;Acc:3703] |
| SEMA3D | Down-regulated | protein_coding | syndecan 1 [Source:HGNC Symbol;Acc:10658] |
| AK5 | Down-regulated | protein_coding | par-3 partitioning defective 3 homolog B (C. elegans) [Source:HGNC Symbol;Acc:14446] |
| THY1 | Down-regulated | protein_coding | 24-dehydrocholesterol reductase [Source:HGNC Symbol;Acc:2859] |
| ANKH | Up-regulated | protein_coding | Ral GEF with PH domain and SH3 binding motif 2 [Source:HGNC Symbol;Acc:30279] |
| PITPNC1 | Up-regulated | protein_coding | family with sequence similarity 20, member B [Source:HGNC Symbol;Acc:23017] |
| ABCA6 | Up-regulated | protein_coding | olfactomedin-like 3 [Source:HGNC Symbol;Acc:24956] |
| SH3RF1 | Down-regulated | protein_coding | regulator of G-protein signaling 4 [Source:HGNC Symbol;Acc:10000] |
| FAM69A | Down-regulated | protein_coding | synovial sarcoma, X breakpoint 2 interacting protein [Source:HGNC Symbol;Acc:16509] |
| JAM2 | Up-regulated | protein_coding | guanylate binding protein 1, interferon-inducible [Source:HGNC Symbol;Acc:4182] |
| ADAMTS1 | Down-regulated | protein_coding | inhibitor of DNA binding 3, dominant negative helix-loop-helix protein [Source:HGNC Symbol;Acc:5362] |
| VOPP1 | Down-regulated | protein_coding | cell division cycle 20 [Source:HGNC Symbol;Acc:1723] |
| KLF10 | Up-regulated | protein_coding | coagulation factor III (thromboplastin, tissue factor) [Source:HGNC Symbol;Acc:3541] |
| MARCKS | Down-regulated | protein_coding | stathmin 1 [Source:HGNC Symbol;Acc:6510] |
| HSPA13 | Up-regulated | protein_coding | centromere protein F, 350/400kDa [Source:HGNC Symbol;Acc:1857] |
| GRAMD3 | Down-regulated | protein_coding | Kruppel-like factor 7 (ubiquitous) [Source:HGNC Symbol;Acc:6350] |
| SLC16A1 | Up-regulated | protein_coding | connective tissue growth factor [Source:HGNC Symbol;Acc:2500] |
| FZD7 | Down-regulated | protein_coding | myosin, light chain 12B, regulatory [Source:HGNC Symbol;Acc:29827] |
| FMN2 | Down-regulated | protein_coding | coiled-coil domain containing 92 [Source:HGNC Symbol;Acc:29563] |
| ACPL2 | Up-regulated | protein_coding | catenin (cadherin-associated protein), alpha-like 1 [Source:HGNC Symbol;Acc:2512] |
| PSD3 | Up-regulated | protein_coding | PHD finger protein 19 [Source:HGNC Symbol;Acc:24566] |
| MCU | Down-regulated | protein_coding | protein phosphatase 1, regulatory subunit 3C [Source:HGNC Symbol;Acc:9293] |
| GDF6 | Down-regulated | protein_coding | methylenetetrahydrofolate dehydrogenase (NADP+ dependent) 1-like [Source:HGNC Symbol;Acc:21055] |
| SST | Up-regulated | protein_coding | acetyl-CoA acetyltransferase 2 [Source:HGNC Symbol;Acc:94] |
| MMP14 | Up-regulated | protein_coding | SMAD family member 9 [Source:HGNC Symbol;Acc:6774] |
| MYO1E | Down-regulated | protein_coding | transforming growth factor, beta-induced, 68kDa [Source:HGNC Symbol;Acc:11771] |
| ETS2 | Up-regulated | protein_coding | thymopoietin [Source:HGNC Symbol;Acc:11875] |
| TSPAN18 | Down-regulated | protein_coding | sorbin and SH3 domain containing 3 [Source:HGNC Symbol;Acc:30907] |
| CREB3L1 | Down-regulated | protein_coding | PDZ and LIM domain 2 (mystique) [Source:HGNC Symbol;Acc:13992] |
| ACAN | Down-regulated | protein_coding | annexin A11 [Source:HGNC Symbol;Acc:535] |
| FMNL2 | Up-regulated | protein_coding | reversion-inducing-cysteine-rich protein with kazal motifs [Source:HGNC Symbol;Acc:11345] |
| XDH | Down-regulated | protein_coding | caldesmon 1 [Source:HGNC Symbol;Acc:1441] |
| MRAS | Down-regulated | protein_coding | GIPC PDZ domain containing family, member 1 [Source:HGNC Symbol;Acc:1226] |
| COLEC12 | Down-regulated | protein_coding | neurolysin (metallopeptidase M3 family) [Source:HGNC Symbol;Acc:16058] |
| CUL4B | Down-regulated | protein_coding | tubulin, alpha 1b [Source:HGNC Symbol;Acc:18809] |
| GPR153 | Up-regulated | protein_coding | tocopherol (alpha) transfer protein-like [Source:HGNC Symbol;Acc:16114] |
| AUTS2 | Up-regulated | protein_coding | prostaglandin I2 (prostacyclin) synthase [Source:HGNC Symbol;Acc:9603] |
| NBL1 | Up-regulated | protein_coding | serpin peptidase inhibitor, clade B (ovalbumin), member 6 [Source:HGNC Symbol;Acc:8950] |
| ADAMTS4 | Up-regulated | protein_coding | AHNAK nucleoprotein [Source:HGNC Symbol;Acc:347] |
| CACHD1 | Up-regulated | protein_coding | FBJ murine osteosarcoma viral oncogene homolog B [Source:HGNC Symbol;Acc:3797] |
| IFNGR2 | Up-regulated | protein_coding | vasodilator-stimulated phosphoprotein [Source:HGNC Symbol;Acc:12652] |
| STC1 | Up-regulated | protein_coding | destrin (actin depolymerizing factor) [Source:HGNC Symbol;Acc:15750] |
| CSRP1 | Down-regulated | protein_coding | inhibitor of DNA binding 1, dominant negative helix-loop-helix protein [Source:HGNC Symbol;Acc:5360] |
| ACTC1 | Down-regulated | protein_coding | angiomotin [Source:HGNC Symbol;Acc:17810] |
| SPON2 | Down-regulated | protein_coding | discs, large (Drosophila) homolog-associated protein 5 [Source:HGNC Symbol;Acc:16864] |
| ZYX | Down-regulated | protein_coding | heat shock 70kDa protein 2 [Source:HGNC Symbol;Acc:5235] |
| CBS | Down-regulated | protein_coding | Kruppel-like factor 2 (lung) [Source:HGNC Symbol;Acc:6347] |
| ICOSLG | Up-regulated | protein_coding | ZFP36 ring finger protein [Source:HGNC Symbol;Acc:12862] |
| PCSK7 | Down-regulated | protein_coding | tyrosine 3-monooxygenase/tryptophan 5-monooxygenase activation protein, eta polypeptide [Source:HGNC Symbol;Acc:12853] |
| LY6K | Down-regulated | protein_coding | CDC42 effector protein (Rho GTPase binding) 1 [Source:HGNC Symbol;Acc:17014] |
| SQSTM1 | Up-regulated | protein_coding | carboxypeptidase A4 [Source:HGNC Symbol;Acc:15740] |
| MFSD12 | Up-regulated | protein_coding | podocalyxin-like [Source:HGNC Symbol;Acc:9171] |
| CYGB | Up-regulated | protein_coding | filamin C, gamma [Source:HGNC Symbol;Acc:3756] |
| CCL5 | Up-regulated | protein_coding | tropomodulin 2 (neuronal) [Source:HGNC Symbol;Acc:11872] |
| PLCD3 | Down-regulated | protein_coding | family with sequence similarity 63, member B [Source:HGNC Symbol;Acc:26954] |
| FMNL3 | Up-regulated | protein_coding | chemokine (C-C motif) receptor-like 1 [Source:HGNC Symbol;Acc:1611] |
| RACGAP1 | Down-regulated | protein_coding | palladin, cytoskeletal associated protein [Source:HGNC Symbol;Acc:17068] |
| SPC24 | Down-regulated | protein_coding | family with sequence similarity 64, member A [Source:HGNC Symbol;Acc:25483] |
| ADCY9 | Down-regulated | protein_coding | solute carrier family 44, member 2 [Source:HGNC Symbol;Acc:17292] |
| RPS6KA4 | Down-regulated | protein_coding | ajuba LIM protein [Source:HGNC Symbol;Acc:20250] |
| PPAP2B | Up-regulated | protein_coding | low density lipoprotein receptor [Source:HGNC Symbol;Acc:6547] |
| AK4 | Up-regulated | protein_coding | calponin 1, basic, smooth muscle [Source:HGNC Symbol;Acc:2155] |
| DRAXIN | Up-regulated | protein_coding | actinin, alpha 4 [Source:HGNC Symbol;Acc:166] |
| PDPN | Up-regulated | protein_coding | H19, imprinted maternally expressed transcript (non-protein coding) [Source:HGNC Symbol;Acc:4713] |
| DHRS3 | Down-regulated | protein_coding | neuronal PAS domain protein 1 [Source:HGNC Symbol;Acc:7894] |
| SDC3 | Down-regulated | protein_coding | A kinase (PRKA) anchor protein 12 [Source:HGNC Symbol;Acc:370] |
| SYNC | Down-regulated | protein_coding | spectrin repeat containing, nuclear envelope 1 [Source:HGNC Symbol;Acc:17089] |
| ALPL | Down-regulated | protein_coding | CAP, adenylate cyclase-associated protein 1 (yeast) [Source:HGNC Symbol;Acc:20040] |
| MEGF6 | Down-regulated | protein_coding | diaphanous homolog 1 (Drosophila) [Source:HGNC Symbol;Acc:2876] |
| DIRAS3 | Up-regulated | protein_coding | microtubule-associated protein 1B [Source:HGNC Symbol;Acc:6836] |
| NEXN | Down-regulated | protein_coding | keratin 34 [Source:HGNC Symbol;Acc:6452] |
| DNAJB4 | Down-regulated | protein_coding | matrilin 2 [Source:HGNC Symbol;Acc:6908] |
| SNX7 | Down-regulated | protein_coding | KN motif and ankyrin repeat domains 4 [Source:HGNC Symbol;Acc:27263] |
| NTNG1 | Down-regulated | protein_coding | myosin, heavy chain 10, non-muscle [Source:HGNC Symbol;Acc:7568] |
| VCAM1 | Up-regulated | protein_coding | myosin phosphatase Rho interacting protein [Source:HGNC Symbol;Acc:30321] |
| ARPC5 | Down-regulated | protein_coding | periostin, osteoblast specific factor [Source:HGNC Symbol;Acc:16953] |
| PEA15 | Down-regulated | protein_coding | myosin, heavy chain 11, smooth muscle [Source:HGNC Symbol;Acc:7569] |
| SNED1 | Up-regulated | protein_coding | SWAP switching B-cell complex 70kDa subunit [Source:HGNC Symbol;Acc:17070] |
| KIF26B | Down-regulated | protein_coding | microtubule associated monooxygenase, calponin and LIM domain containing 2 [Source:HGNC Symbol;Acc:24693] |
| IL24 | Up-regulated | protein_coding | related RAS viral (r-ras) oncogene homolog 2 [Source:HGNC Symbol;Acc:17271] |
| ACTG2 | Down-regulated | protein_coding | CBP80/20-dependent translation initiation factor [Source:HGNC Symbol;Acc:23925] |
| SMC6 | Down-regulated | protein_coding | cyclin B1 [Source:HGNC Symbol;Acc:1579] |
| PDLIM5 | Down-regulated | protein_coding | Meis homeobox 2 [Source:HGNC Symbol;Acc:7001] |
| CTSS | Up-regulated | protein_coding | proline/serine-rich coiled-coil 1 [Source:HGNC Symbol;Acc:24472] |
| CDC42EP3 | Down-regulated | protein_coding | sortilin 1 [Source:HGNC Symbol;Acc:11186] |
| IGFN1 | Up-regulated | protein_coding | nerve growth factor (beta polypeptide) [Source:HGNC Symbol;Acc:7808] |
| LMOD1 | Down-regulated | protein_coding | transmembrane protein 106C [Source:HGNC Symbol;Acc:28775] |
| NFASC | Down-regulated | protein_coding | solute carrier family 38, member 2 [Source:HGNC Symbol;Acc:13448] |
| PTPN13 | Down-regulated | protein_coding | Rho-associated, coiled-coil containing protein kinase 2 [Source:HGNC Symbol;Acc:10252] |
| ADAMTS9 | Up-regulated | protein_coding | SPOC domain containing 1 [Source:HGNC Symbol;Acc:26338] |
| PTX3 | Down-regulated | protein_coding | cell division cycle associated 8 [Source:HGNC Symbol;Acc:14629] |
| APBB2 | Down-regulated | protein_coding | fatty acid desaturase 2 [Source:HGNC Symbol;Acc:3575] |
| CXCL3 | Up-regulated | protein_coding | annexin A1 [Source:HGNC Symbol;Acc:533] |
| CXCL5 | Up-regulated | protein_coding | ADAM metallopeptidase domain 19 [Source:HGNC Symbol;Acc:197] |
| CXCL1 | Up-regulated | protein_coding | integrin, alpha 7 [Source:HGNC Symbol;Acc:6143] |
| CDCP1 | Up-regulated | protein_coding | keratin 7 [Source:HGNC Symbol;Acc:6445] |
| ZC3H12A | Up-regulated | protein_coding | microtubule associated monooxygenase, calponin and LIM domain containing 1 [Source:HGNC Symbol;Acc:20619] |
| SGMS2 | Down-regulated | protein_coding | dedicator of cytokinesis 10 [Source:HGNC Symbol;Acc:23479] |
| SPRY1 | Up-regulated | protein_coding | plexin C1 [Source:HGNC Symbol;Acc:9106] |
| PRSS12 | Down-regulated | protein_coding | adaptor protein, phosphotyrosine interaction, PH domain and leucine zipper containing 2 [Source:HGNC Symbol;Acc:18242] |
| HMGB2 | Down-regulated | protein_coding | DNA-damage regulated autophagy modulator 1 [Source:HGNC Symbol;Acc:25645] |
| ITGA2 | Up-regulated | protein_coding | filamin B, beta [Source:HGNC Symbol;Acc:3755] |
| EDIL3 | Up-regulated | protein_coding | cytoskeleton associated protein 2 [Source:HGNC Symbol;Acc:1990] |
| STARD4 | Down-regulated | protein_coding | LIM domain 7 [Source:HGNC Symbol;Acc:6646] |
| RHOBTB3 | Down-regulated | protein_coding | tensin 3 [Source:HGNC Symbol;Acc:21616] |
| GRIK2 | Down-regulated | protein_coding | ADAM metallopeptidase with thrombospondin type 1 motif, 7 [Source:HGNC Symbol;Acc:223] |
| CITED2 | Down-regulated | protein_coding | nipsnap homolog 3A (C. elegans) [Source:HGNC Symbol;Acc:23619] |
| DCBLD1 | Down-regulated | protein_coding | ectonucleotide pyrophosphatase/phosphodiesterase 2 [Source:HGNC Symbol;Acc:3357] |
| STK17A | Down-regulated | protein_coding | talin 1 [Source:HGNC Symbol;Acc:11845] |
| GALNT10 | Down-regulated | protein_coding | transcription factor 19 [Source:HGNC Symbol;Acc:11629] |
| SBSPON | Down-regulated | protein_coding | flotillin 1 [Source:HGNC Symbol;Acc:3757] |
| OSGIN2 | Up-regulated | protein_coding | synaptotagmin-like 2 [Source:HGNC Symbol;Acc:15585] |
| ALDH7A1 | Down-regulated | protein_coding | sulfatase 1 [Source:HGNC Symbol;Acc:20391] |
| OSR2 | Down-regulated | protein_coding | transient receptor potential cation channel, subfamily C, member 6 [Source:HGNC Symbol;Acc:12338] |
| GEM | Up-regulated | protein_coding | thrombospondin 1 [Source:HGNC Symbol;Acc:11785] |
| ABCA1 | Up-regulated | protein_coding | kinesin family member 23 [Source:HGNC Symbol;Acc:6392] |
| NFIL3 | Up-regulated | protein_coding | integrin, alpha 11 [Source:HGNC Symbol;Acc:6136] |
| ALDH1A1 | Down-regulated | protein_coding | cancer susceptibility candidate 5 [Source:HGNC Symbol;Acc:24054] |
| SVEP1 | Down-regulated | protein_coding | uveal autoantigen with coiled-coil domains and ankyrin repeats [Source:HGNC Symbol;Acc:15947] |
| MELK | Down-regulated | protein_coding | SMAD family member 6 [Source:HGNC Symbol;Acc:6772] |
| SPTSSA | Up-regulated | protein_coding | breast cancer anti-estrogen resistance 3 [Source:HGNC Symbol;Acc:973] |
| CFL2 | Down-regulated | protein_coding | Rho GTPase activating protein 29 [Source:HGNC Symbol;Acc:30207] |
| PGM2L1 | Up-regulated | protein_coding | myoferlin [Source:HGNC Symbol;Acc:3656] |
| GJB2 | Up-regulated | protein_coding | lysyl oxidase-like 4 [Source:HGNC Symbol;Acc:17171] |
| VSTM4 | Up-regulated | protein_coding | kinesin family member 11 [Source:HGNC Symbol;Acc:6388] |
| ZNF503 | Up-regulated | protein_coding | centrosomal protein 55kDa [Source:HGNC Symbol;Acc:1161] |
| KIAA1462 | Down-regulated | protein_coding | phospholipase C, epsilon 1 [Source:HGNC Symbol;Acc:17175] |
| ARHGEF40 | Up-regulated | protein_coding | myopalladin [Source:HGNC Symbol;Acc:23246] |
| CASP7 | Up-regulated | protein_coding | HECT and RLD domain containing E3 ubiquitin protein ligase 3 [Source:HGNC Symbol;Acc:4876] |
| PTPLA | Down-regulated | protein_coding | fibroblast growth factor 5 [Source:HGNC Symbol;Acc:3683] |
| ABTB2 | Up-regulated | protein_coding | phosphodiesterase 5A, cGMP-specific [Source:HGNC Symbol;Acc:8784] |
| HTRA1 | Up-regulated | protein_coding | septin 11 [Source:HGNC Symbol;Acc:25589] |
| SPRED1 | Up-regulated | protein_coding | shroom family member 3 [Source:HGNC Symbol;Acc:30422] |
| GPR176 | Down-regulated | protein_coding | fibrillin 2 [Source:HGNC Symbol;Acc:3604] |
| CKB | Down-regulated | protein_coding | adhesion molecule with Ig-like domain 2 [Source:HGNC Symbol;Acc:24073] |
| CLMP | Up-regulated | protein_coding | GLI pathogenesis-related 1 [Source:HGNC Symbol;Acc:17001] |
| TMEM100 | Up-regulated | protein_coding | leucine-rich repeat containing G protein-coupled receptor 5 [Source:HGNC Symbol;Acc:4504] |
| CYB5A | Down-regulated | protein_coding | neural precursor cell expressed, developmentally down-regulated 1 [Source:HGNC Symbol;Acc:7723] |
| SERPINB7 | Down-regulated | protein_coding | solute carrier family 7 (cationic amino acid transporter, y+ system), member 1 [Source:HGNC Symbol;Acc:11057] |
| MFAP4 | Up-regulated | protein_coding | extended synaptotagmin-like protein 1 [Source:HGNC Symbol;Acc:29534] |
| MMP10 | Up-regulated | protein_coding | diaphanous homolog 3 (Drosophila) [Source:HGNC Symbol;Acc:15480] |
| B2M | Up-regulated | protein_coding | muscleblind-like splicing regulator 2 [Source:HGNC Symbol;Acc:16746] |
| C16orf45 | Up-regulated | protein_coding | somatostatin receptor 1 [Source:HGNC Symbol;Acc:11330] |
| ANPEP | Up-regulated | protein_coding | FERM domain containing 6 [Source:HGNC Symbol;Acc:19839] |
| NAV2 | Down-regulated | protein_coding | fibulin 5 [Source:HGNC Symbol;Acc:3602] |
| PLK1 | Down-regulated | protein_coding | tropomyosin 1 (alpha) [Source:HGNC Symbol;Acc:12010] |
| MTMR10 | Down-regulated | protein_coding | arrestin domain containing 4 [Source:HGNC Symbol;Acc:28087] |
| GREM1 | Down-regulated | protein_coding | transforming growth factor beta 1 induced transcript 1 [Source:HGNC Symbol;Acc:11767] |
| SMAD3 | Down-regulated | protein_coding | Rho GTPase activating protein 17 [Source:HGNC Symbol;Acc:18239] |
| MAP1A | Down-regulated | protein_coding | NLR family, CARD domain containing 5 [Source:HGNC Symbol;Acc:29933] |
| TBC1D16 | Up-regulated | protein_coding | TBC/LysM-associated domain containing 1 [Source:HGNC Symbol;Acc:29325] |
| TUBA1A | Down-regulated | protein_coding | myocardin [Source:HGNC Symbol;Acc:16067] |
| TUBA1C | Down-regulated | protein_coding | v-erb-b2 erythroblastic leukemia viral oncogene homolog 2, neuro/glioblastoma derived oncogene homolog (avian) [Source:HGNC Symbol;Acc:3430] |
| AXL | Down-regulated | protein_coding | dopey family member 2 [Source:HGNC Symbol;Acc:1291] |
| PPP1R14A | Down-regulated | protein_coding | Wilms tumor 1 interacting protein [Source:HGNC Symbol;Acc:20964] |
| DAPK3 | Down-regulated | protein_coding | EPH receptor A2 [Source:HGNC Symbol;Acc:3386] |
| SLC43A2 | Up-regulated | protein_coding | SH3 domain binding glutamic acid-rich protein like 3 [Source:HGNC Symbol;Acc:15568] |
| ANGPTL4 | Up-regulated | protein_coding | heparan sulfate proteoglycan 2 [Source:HGNC Symbol;Acc:5273] |
| IGFBP6 | Down-regulated | protein_coding | cysteine-rich, angiogenic inducer, 61 [Source:HGNC Symbol;Acc:2654] |
| TK1 | Down-regulated | protein_coding | tubulointerstitial nephritis antigen-like 1 [Source:HGNC Symbol;Acc:19168] |
| FTH1 | Up-regulated | protein_coding | protein tyrosine phosphatase, receptor type, F [Source:HGNC Symbol;Acc:9670] |
| SCARA5 | Up-regulated | protein_coding | calmodulin 2 (phosphorylase kinase, delta) [Source:HGNC Symbol;Acc:1445] |
| TTC39C | Down-regulated | protein_coding | AF4/FMR2 family, member 3 [Source:HGNC Symbol;Acc:6473] |
| FILIP1L | Down-regulated | protein_coding | membrane-associated ring finger (C3HC4) 4, E3 ubiquitin protein ligase [Source:HGNC Symbol;Acc:29269] |
| MFSD2A | Up-regulated | protein_coding | pleckstrin homology-like domain, family B, member 2 [Source:HGNC Symbol;Acc:29573] |
| BDKRB2 | Up-regulated | protein_coding | neutral cholesterol ester hydrolase 1 [Source:HGNC Symbol;Acc:29260] |
| RAB31 | Up-regulated | protein_coding | KIAA0226 [Source:HGNC Symbol;Acc:28991] |
| TNXB | Down-regulated | protein_coding | T-cell leukemia translocation altered [Source:HGNC Symbol;Acc:11692] |
| BMP1 | Up-regulated | protein_coding | slit homolog 2 (Drosophila) [Source:HGNC Symbol;Acc:11086] |
| SDPR | Down-regulated | protein_coding | EPH receptor A5 [Source:HGNC Symbol;Acc:3389] |
| SOGA2 | Down-regulated | protein_coding | cyclin A2 [Source:HGNC Symbol;Acc:1578] |
| COL3A1 | Up-regulated | protein_coding | ubiquitin specific peptidase 53 [Source:HGNC Symbol;Acc:29255] |
| GFRA2 | Up-regulated | protein_coding | myosin X [Source:HGNC Symbol;Acc:7593] |
| IL7R | Down-regulated | protein_coding | S-phase kinase-associated protein 2, E3 ubiquitin protein ligase [Source:HGNC Symbol;Acc:10901] |
| SEMA4C | Up-regulated | protein_coding | teneurin transmembrane protein 2 [Source:HGNC Symbol;Acc:29943] |
| ATOH8 | Down-regulated | protein_coding | dishevelled associated activator of morphogenesis 2 [Source:HGNC Symbol;Acc:18143] |
| SLC35G2 | Up-regulated | protein_coding | signal peptide, CUB domain, EGF-like 3 [Source:HGNC Symbol;Acc:13655] |
| PXDC1 | Down-regulated | protein_coding | Rho GTPase activating protein 18 [Source:HGNC Symbol;Acc:21035] |
| ROR2 | Up-regulated | protein_coding | insulin-like growth factor binding protein 3 [Source:HGNC Symbol;Acc:5472] |
| FAM110B | Up-regulated | protein_coding | non-SMC condensin II complex, subunit G2 [Source:HGNC Symbol;Acc:21904] |
| MN1 | Down-regulated | protein_coding | transmembrane protein 47 [Source:HGNC Symbol;Acc:18515] |
| RAB3B | Down-regulated | protein_coding | solute carrier family 16, member 2 (thyroid hormone transporter) [Source:HGNC Symbol;Acc:10923] |
| IL8 | Up-regulated | protein_coding | phosphoribosyl pyrophosphate synthetase 1 [Source:HGNC Symbol;Acc:9462] |
| SCN9A | Down-regulated | protein_coding | chromosome 9 open reading frame 3 [Source:HGNC Symbol;Acc:1361] |
| COL22A1 | Up-regulated | protein_coding | stomatin [Source:HGNC Symbol;Acc:3383] |
| PLEKHA2 | Down-regulated | protein_coding | Ras suppressor protein 1 [Source:HGNC Symbol;Acc:10464] |
| SLC38A11 | Down-regulated | protein_coding | HECT and RLD domain containing E3 ubiquitin protein ligase 4 [Source:HGNC Symbol;Acc:24521] |
| BUB1 | Down-regulated | protein_coding | calcium/calmodulin-dependent protein kinase II gamma [Source:HGNC Symbol;Acc:1463] |
| TM4SF1 | Down-regulated | protein_coding | ankyrin repeat domain 1 (cardiac muscle) [Source:HGNC Symbol;Acc:15819] |
| ALCAM | Down-regulated | protein_coding | antigen identified by monoclonal antibody Ki-67 [Source:HGNC Symbol;Acc:7107] |
| TRAPPC1 | Down-regulated | protein_coding | endonuclease domain containing 1 [Source:HGNC Symbol;Acc:29129] |
| RNF150 | Down-regulated | protein_coding | ADAM metallopeptidase domain 33 [Source:HGNC Symbol;Acc:15478] |
| DCLK2 | Down-regulated | protein_coding | kin of IRRE like 3 (Drosophila) [Source:HGNC Symbol;Acc:23204] |
| PFKFB3 | Up-regulated | protein_coding | transgelin [Source:HGNC Symbol;Acc:11553] |
| CDH2 | Down-regulated | protein_coding | junctophilin 2 [Source:HGNC Symbol;Acc:14202] |
| SOCS6 | Up-regulated | protein_coding | KIAA1755 [Source:HGNC Symbol;Acc:29372] |
| HTRA3 | Up-regulated | protein_coding | phospholipase C, beta 3 (phosphatidylinositol-specific) [Source:HGNC Symbol;Acc:9056] |
| TANC2 | Down-regulated | protein_coding | mohawk homeobox [Source:HGNC Symbol;Acc:23729] |
| HAS2 | Up-regulated | protein_coding | adrenoceptor alpha 2A [Source:HGNC Symbol;Acc:281] |
| FEZ2 | Down-regulated | protein_coding | vascular endothelial growth factor C [Source:HGNC Symbol;Acc:12682] |
| PRKCE | Up-regulated | protein_coding | protease, serine, 23 [Source:HGNC Symbol;Acc:14370] |
| C9orf16 | Up-regulated | protein_coding | cysteine rich transmembrane BMP regulator 1 (chordin-like) [Source:HGNC Symbol;Acc:2359] |
| JUNB | Up-regulated | protein_coding | calcium channel, voltage-dependent, L type, alpha 1C subunit [Source:HGNC Symbol;Acc:1390] |
| SHCBP1 | Down-regulated | protein_coding | membrane associated guanylate kinase, WW and PDZ domain containing 1 [Source:HGNC Symbol;Acc:946] |
| CHST11 | Up-regulated | protein_coding | NIMA-related kinase 7 [Source:HGNC Symbol;Acc:13386] |
| KRT19 | Down-regulated | protein_coding | epidermal growth factor receptor pathway substrate 8 [Source:HGNC Symbol;Acc:3420] |
| KCND3 | Down-regulated | protein_coding | WW and C2 domain containing 2 [Source:HGNC Symbol;Acc:24148] |
| PDE7B | Up-regulated | protein_coding | solute carrier family 25 (mitochondrial carrier; adenine nucleotide translocator), member 4 [Source:HGNC Symbol;Acc:10990] |
| RSL1D1 | Up-regulated | protein_coding | dystonin [Source:HGNC Symbol;Acc:1090] |
| PTGER4 | Up-regulated | protein_coding | protein tyrosine phosphatase, non-receptor type 14 [Source:HGNC Symbol;Acc:9647] |
| SPSB1 | Up-regulated | protein_coding | ectopic P-granules autophagy protein 5 homolog (C. elegans) [Source:HGNC Symbol;Acc:29331] |
| TMEM51 | Up-regulated | protein_coding | muscleblind-like splicing regulator 1 [Source:HGNC Symbol;Acc:6923] |
| COL8A2 | Down-regulated | protein_coding | FERM, RhoGEF (ARHGEF) and pleckstrin domain protein 1 (chondrocyte-derived) [Source:HGNC Symbol;Acc:3591] |
| RRM2 | Down-regulated | protein_coding | utrophin [Source:HGNC Symbol;Acc:12635] |
| SCG2 | Up-regulated | protein_coding | Dab, mitogen-responsive phosphoprotein, homolog 2 (Drosophila) [Source:HGNC Symbol;Acc:2662] |
| GAP43 | Up-regulated | protein_coding | dimethylarginine dimethylaminohydrolase 1 [Source:HGNC Symbol;Acc:2715] |
| LRRC15 | Down-regulated | protein_coding | sema domain, immunoglobulin domain (Ig), short basic domain, secreted, (semaphorin) 3D [Source:HGNC Symbol;Acc:10726] |
| ISG20 | Up-regulated | protein_coding | adenylate kinase 5 [Source:HGNC Symbol;Acc:365] |
| CEBPB | Up-regulated | protein_coding | Thy-1 cell surface antigen [Source:HGNC Symbol;Acc:11801] |
| BSG | Up-regulated | protein_coding | SH3 domain containing ring finger 1 [Source:HGNC Symbol;Acc:17650] |
| CSDC2 | Down-regulated | protein_coding | family with sequence similarity 69, member A [Source:HGNC Symbol;Acc:32213] |
| RCAN2 | Down-regulated | protein_coding | ADAM metallopeptidase with thrombospondin type 1 motif, 1 [Source:HGNC Symbol;Acc:217] |
| SYNPO2 | Down-regulated | protein_coding | vesicular, overexpressed in cancer, prosurvival protein 1 [Source:HGNC Symbol;Acc:34518] |
| PDE3A | Up-regulated | protein_coding | myristoylated alanine-rich protein kinase C substrate [Source:HGNC Symbol;Acc:6759] |
| CFL1 | Down-regulated | protein_coding | GRAM domain containing 3 [Source:HGNC Symbol;Acc:24911] |
| SP3 | Up-regulated | protein_coding | frizzled family receptor 7 [Source:HGNC Symbol;Acc:4045] |
| DHCR7 | Down-regulated | protein_coding | formin 2 [Source:HGNC Symbol;Acc:14074] |
| AC068491.1 | Down-regulated | processed_transcript | mitochondrial calcium uniporter [Source:HGNC Symbol;Acc:23526] |
| GLRX | Up-regulated | protein_coding | growth differentiation factor 6 [Source:HGNC Symbol;Acc:4221] |
| PEAK1 | Down-regulated | protein_coding | myosin IE [Source:HGNC Symbol;Acc:7599] |
| TNFRSF10D | Up-regulated | protein_coding | tetraspanin 18 [Source:HGNC Symbol;Acc:20660] |
| NABP1 | Down-regulated | protein_coding | cAMP responsive element binding protein 3-like 1 [Source:HGNC Symbol;Acc:18856] |
| PC | Up-regulated | protein_coding | aggrecan [Source:HGNC Symbol;Acc:319] |
| HSPB7 | Down-regulated | protein_coding | xanthine dehydrogenase [Source:HGNC Symbol;Acc:12805] |
| HEG1 | Down-regulated | protein_coding | muscle RAS oncogene homolog [Source:HGNC Symbol;Acc:7227] |
| CD7 | Up-regulated | protein_coding | collectin sub-family member 12 [Source:HGNC Symbol;Acc:16016] |
| JUP | Up-regulated | protein_coding | cullin 4B [Source:HGNC Symbol;Acc:2555] |
| C1QTNF1 | Up-regulated | protein_coding | cysteine and glycine-rich protein 1 [Source:HGNC Symbol;Acc:2469] |
| KLHL15 | Up-regulated | protein_coding | actin, alpha, cardiac muscle 1 [Source:HGNC Symbol;Acc:143] |
| CTSF | Up-regulated | protein_coding | zyxin [Source:HGNC Symbol;Acc:13200] |
| PIK3R6 | Up-regulated | processed_transcript | cystathionine-beta-synthase [Source:HGNC Symbol;Acc:1550] |
| MSRB3 | Down-regulated | protein_coding | proprotein convertase subtilisin/kexin type 7 [Source:HGNC Symbol;Acc:8748] |
| RGMB | Down-regulated | protein_coding | lymphocyte antigen 6 complex, locus K [Source:HGNC Symbol;Acc:24225] |
| PODN | Down-regulated | protein_coding | phospholipase C, delta 3 [Source:HGNC Symbol;Acc:9061] |
| SH3PXD2B | Up-regulated | protein_coding | Rac GTPase activating protein 1 [Source:HGNC Symbol;Acc:9804] |
| SEZ6L2 | Up-regulated | protein_coding | SPC24, NDC80 kinetochore complex component, homolog (S. cerevisiae) [Source:HGNC Symbol;Acc:26913] |
| CHST2 | Up-regulated | protein_coding | adenylate cyclase 9 [Source:HGNC Symbol;Acc:240] |
| MARCKSL1 | Up-regulated | protein_coding | ribosomal protein S6 kinase, 90kDa, polypeptide 4 [Source:HGNC Symbol;Acc:10433] |
| TP53I11 | Down-regulated | protein_coding | dehydrogenase/reductase (SDR family) member 3 [Source:HGNC Symbol;Acc:17693] |
| CLTB | Down-regulated | protein_coding | syndecan 3 [Source:HGNC Symbol;Acc:10660] |
| PCSK1 | Up-regulated | protein_coding | syncoilin, intermediate filament protein [Source:HGNC Symbol;Acc:28897] |
| TOM1L2 | Down-regulated | protein_coding | alkaline phosphatase, liver/bone/kidney [Source:HGNC Symbol;Acc:438] |
| ETV4 | Up-regulated | protein_coding | multiple EGF-like-domains 6 [Source:HGNC Symbol;Acc:3232] |
| A2M | Up-regulated | protein_coding | nexilin (F actin binding protein) [Source:HGNC Symbol;Acc:29557] |
| TUBB6 | Down-regulated | protein_coding | DnaJ (Hsp40) homolog, subfamily B, member 4 [Source:HGNC Symbol;Acc:14886] |
| BNIP3 | Up-regulated | protein_coding | sorting nexin 7 [Source:HGNC Symbol;Acc:14971] |
| RIMS2 | Up-regulated | protein_coding | netrin G1 [Source:HGNC Symbol;Acc:23319] |
| B3GNT5 | Up-regulated | protein_coding | actin related protein 2/3 complex, subunit 5, 16kDa [Source:HGNC Symbol;Acc:708] |
| LMNB2 | Down-regulated | protein_coding | phosphoprotein enriched in astrocytes 15 [Source:HGNC Symbol;Acc:8822] |
| BOK | Down-regulated | protein_coding | kinesin family member 26B [Source:HGNC Symbol;Acc:25484] |
| BASP1 | Down-regulated | protein_coding | actin, gamma 2, smooth muscle, enteric [Source:HGNC Symbol;Acc:145] |
| TYMS | Down-regulated | protein_coding | structural maintenance of chromosomes 6 [Source:HGNC Symbol;Acc:20466] |
| C8orf4 | Up-regulated | protein_coding | PDZ and LIM domain 5 [Source:HGNC Symbol;Acc:17468] |
| DPP7 | Up-regulated | protein_coding | CDC42 effector protein (Rho GTPase binding) 3 [Source:HGNC Symbol;Acc:16943] |
| EPS8L2 | Down-regulated | protein_coding | leiomodin 1 (smooth muscle) [Source:HGNC Symbol;Acc:6647] |
| PDDC1 | Down-regulated | protein_coding | neurofascin [Source:HGNC Symbol;Acc:29866] |
| FZD8 | Up-regulated | protein_coding | protein tyrosine phosphatase, non-receptor type 13 (APO-1/CD95 (Fas)-associated phosphatase) [Source:HGNC Symbol;Acc:9646] |
| LRRN4CL | Up-regulated | protein_coding | pentraxin 3, long [Source:HGNC Symbol;Acc:9692] |
| PAWR | Down-regulated | protein_coding | amyloid beta (A4) precursor protein-binding, family B, member 2 [Source:HGNC Symbol;Acc:582] |
| TGIF1 | Up-regulated | protein_coding | sphingomyelin synthase 2 [Source:HGNC Symbol;Acc:28395] |
| PTRF | Down-regulated | protein_coding | protease, serine, 12 (neurotrypsin, motopsin) [Source:HGNC Symbol;Acc:9477] |
| POLR2L | Down-regulated | protein_coding | high mobility group box 2 [Source:HGNC Symbol;Acc:5000] |
| FAM20C | Up-regulated | protein_coding | StAR-related lipid transfer (START) domain containing 4 [Source:HGNC Symbol;Acc:18058] |
| PDE4DIP | Down-regulated | protein_coding | Rho-related BTB domain containing 3 [Source:HGNC Symbol;Acc:18757] |
| PLEC | Down-regulated | protein_coding | glutamate receptor, ionotropic, kainate 2 [Source:HGNC Symbol;Acc:4580] |
| ERN1 | Up-regulated | protein_coding | Cbp/p300-interacting transactivator, with Glu/Asp-rich carboxy-terminal domain, 2 [Source:HGNC Symbol;Acc:1987] |
| KCTD12 | Up-regulated | protein_coding | discoidin, CUB and LCCL domain containing 1 [Source:HGNC Symbol;Acc:21479] |
| THBD | Up-regulated | protein_coding | serine/threonine kinase 17a [Source:HGNC Symbol;Acc:11395] |
| PER1 | Up-regulated | protein_coding | UDP-N-acetyl-alpha-D-galactosamine:polypeptide N-acetylgalactosaminyltransferase 10 (GalNAc-T10) [Source:HGNC Symbol;Acc:19873] |
| LDLRAD3 | Up-regulated | protein_coding | somatomedin B and thrombospondin, type 1 domain containing [Source:HGNC Symbol;Acc:30362] |
| EGR3 | Up-regulated | protein_coding | aldehyde dehydrogenase 7 family, member A1 [Source:HGNC Symbol;Acc:877] |
| FZD2 | Down-regulated | protein_coding | odd-skipped related 2 (Drosophila) [Source:HGNC Symbol;Acc:15830] |
| MAB21L1 | Down-regulated | protein_coding | aldehyde dehydrogenase 1 family, member A1 [Source:HGNC Symbol;Acc:402] |
| GREM2 | Down-regulated | protein_coding | sushi, von Willebrand factor type A, EGF and pentraxin domain containing 1 [Source:HGNC Symbol;Acc:15985] |
| SSR4 | Up-regulated | protein_coding | maternal embryonic leucine zipper kinase [Source:HGNC Symbol;Acc:16870] |
| OXTR | Down-regulated | protein_coding | cofilin 2 (muscle) [Source:HGNC Symbol;Acc:1875] |
| TMEM45A | Up-regulated | protein_coding | KIAA1462 [Source:HGNC Symbol;Acc:29283] |
| RAP2B | Down-regulated | protein_coding | protein tyrosine phosphatase-like (proline instead of catalytic arginine), member A [Source:HGNC Symbol;Acc:9639] |
| C5orf30 | Down-regulated | protein_coding | G protein-coupled receptor 176 [Source:HGNC Symbol;Acc:32370] |
| CREB3L2 | Up-regulated | protein_coding | creatine kinase, brain [Source:HGNC Symbol;Acc:1991] |
| EXT1 | Down-regulated | protein_coding | cytochrome b5 type A (microsomal) [Source:HGNC Symbol;Acc:2570] |
| SGK223 | Down-regulated | protein_coding | serpin peptidase inhibitor, clade B (ovalbumin), member 7 [Source:HGNC Symbol;Acc:13902] |
| C1S | Up-regulated | protein_coding | neuron navigator 2 [Source:HGNC Symbol;Acc:15997] |
| CACNB4 | Down-regulated | protein_coding | polo-like kinase 1 [Source:HGNC Symbol;Acc:9077] |
| TSHZ2 | Up-regulated | protein_coding | myotubularin related protein 10 [Source:HGNC Symbol;Acc:25999] |
| TRAK1 | Down-regulated | protein_coding | gremlin 1, DAN family BMP antagonist [Source:HGNC Symbol;Acc:2001] |
| TSKU | Up-regulated | protein_coding | SMAD family member 3 [Source:HGNC Symbol;Acc:6769] |
| ANXA2 | Down-regulated | protein_coding | microtubule-associated protein 1A [Source:HGNC Symbol;Acc:6835] |
| COL18A1 | Up-regulated | protein_coding | tubulin, alpha 1a [Source:HGNC Symbol;Acc:20766] |
| GJC1 | Down-regulated | protein_coding | tubulin, alpha 1c [Source:HGNC Symbol;Acc:20768] |
| SLC8A1 | Down-regulated | protein_coding | AXL receptor tyrosine kinase [Source:HGNC Symbol;Acc:905] |
| GAS6 | Down-regulated | protein_coding | protein phosphatase 1, regulatory (inhibitor) subunit 14A [Source:HGNC Symbol;Acc:14871] |
| CCBE1 | Down-regulated | protein_coding | death-associated protein kinase 3 [Source:HGNC Symbol;Acc:2676] |
| 15-Sep | Up-regulated | protein_coding | insulin-like growth factor binding protein 6 [Source:HGNC Symbol;Acc:5475] |
| FAM101B | Down-regulated | protein_coding | thymidine kinase 1, soluble [Source:HGNC Symbol;Acc:11830] |
| UPP1 | Up-regulated | protein_coding | tetratricopeptide repeat domain 39C [Source:HGNC Symbol;Acc:26595] |
| CMTM4 | Up-regulated | protein_coding | filamin A interacting protein 1-like [Source:HGNC Symbol;Acc:24589] |
| KREMEN1 | Up-regulated | protein_coding | tenascin XB [Source:HGNC Symbol;Acc:11976] |
| IQGAP3 | Down-regulated | protein_coding | serum deprivation response [Source:HGNC Symbol;Acc:10690] |
| ACTG1 | Down-regulated | protein_coding | SOGA family member 2 [Source:HGNC Symbol;Acc:29121] |
| PRKD1 | Down-regulated | protein_coding | interleukin 7 receptor [Source:HGNC Symbol;Acc:6024] |
| CSF1 | Down-regulated | protein_coding | atonal homolog 8 (Drosophila) [Source:HGNC Symbol;Acc:24126] |
| TMEM173 | Down-regulated | protein_coding | PX domain containing 1 [Source:HGNC Symbol;Acc:21361] |
| PDE4B | Up-regulated | protein_coding | meningioma (disrupted in balanced translocation) 1 [Source:HGNC Symbol;Acc:7180] |
| FLRT2 | Up-regulated | protein_coding | RAB3B, member RAS oncogene family [Source:HGNC Symbol;Acc:9778] |
| FAM43A | Up-regulated | protein_coding | sodium channel, voltage-gated, type IX, alpha subunit [Source:HGNC Symbol;Acc:10597] |
| WBP5 | Up-regulated | protein_coding | pleckstrin homology domain containing, family A (phosphoinositide binding specific) member 2 [Source:HGNC Symbol;Acc:14336] |
| UBALD2 | Up-regulated | protein_coding | solute carrier family 38, member 11 [Source:HGNC Symbol;Acc:26836] |
| MUC1 | Down-regulated | protein_coding | BUB1 mitotic checkpoint serine/threonine kinase [Source:HGNC Symbol;Acc:1148] |
| LSAMP | Up-regulated | protein_coding | transmembrane 4 L six family member 1 [Source:HGNC Symbol;Acc:11853] |
| AHNAK2 | Down-regulated | protein_coding | activated leukocyte cell adhesion molecule [Source:HGNC Symbol;Acc:400] |
| OLFML2A | Up-regulated | protein_coding | trafficking protein particle complex 1 [Source:HGNC Symbol;Acc:19894] |
| NDUFA4L2 | Up-regulated | protein_coding | ring finger protein 150 [Source:HGNC Symbol;Acc:23138] |
| C11orf87 | Up-regulated | protein_coding | doublecortin-like kinase 2 [Source:HGNC Symbol;Acc:19002] |
| PCYT2 | Down-regulated | protein_coding | cadherin 2, type 1, N-cadherin (neuronal) [Source:HGNC Symbol;Acc:1759] |
| IFITM1 | Up-regulated | protein_coding | tetratricopeptide repeat, ankyrin repeat and coiled-coil containing 2 [Source:HGNC Symbol;Acc:30212] |
| BCL9L | Down-regulated | protein_coding | fasciculation and elongation protein zeta 2 (zygin II) [Source:HGNC Symbol;Acc:3660] |
| KIF18B | Down-regulated | protein_coding | SHC SH2-domain binding protein 1 [Source:HGNC Symbol;Acc:29547] |
| BTN3A2 | Up-regulated | protein_coding | keratin 19 [Source:HGNC Symbol;Acc:6436] |
| NF2 | Down-regulated | protein_coding | potassium voltage-gated channel, Shal-related subfamily, member 3 [Source:HGNC Symbol;Acc:6239] |
| C17orf58 | Up-regulated | protein_coding | collagen, type VIII, alpha 2 [Source:HGNC Symbol;Acc:2216] |
| BCR | Up-regulated | protein_coding | ribonucleotide reductase M2 [Source:HGNC Symbol;Acc:10452] |
| TPCN1 | Up-regulated | protein_coding | leucine rich repeat containing 15 [Source:HGNC Symbol;Acc:20818] |
| MITF | Down-regulated | protein_coding | cold shock domain containing C2, RNA binding [Source:HGNC Symbol;Acc:30359] |
| AKR1C1 | Up-regulated | protein_coding | regulator of calcineurin 2 [Source:HGNC Symbol;Acc:3041] |
| GCNT1 | Down-regulated | protein_coding | synaptopodin 2 [Source:HGNC Symbol;Acc:17732] |
| FNBP1 | Down-regulated | protein_coding | cofilin 1 (non-muscle) [Source:HGNC Symbol;Acc:1874] |
| C11orf96 | Up-regulated | protein_coding | 7-dehydrocholesterol reductase [Source:HGNC Symbol;Acc:2860] |
| COL4A1 | Up-regulated | protein_coding | - |
| MAGEH1 | Up-regulated | protein_coding | Homo sapiens NKF3 kinase family member (PEAK1), mRNA. [Source:RefSeq mRNA;Acc:NM_024776] |
| SAMD11 | Down-regulated | protein_coding | nucleic acid binding protein 1 [Source:HGNC Symbol;Acc:26232] |
| SPRY4 | Up-regulated | protein_coding | heat shock 27kDa protein family, member 7 (cardiovascular) [Source:HGNC Symbol;Acc:5249] |
| PEAR1 | Down-regulated | protein_coding | heart development protein with EGF-like domains 1 [Source:HGNC Symbol;Acc:29227] |
| DNER | Up-regulated | protein_coding | methionine sulfoxide reductase B3 [Source:HGNC Symbol;Acc:27375] |
| C6orf132 | Down-regulated | protein_coding | RGM domain family, member B [Source:HGNC Symbol;Acc:26896] |
| TUBB4B | Down-regulated | protein_coding | podocan [Source:HGNC Symbol;Acc:23174] |
| C15orf52 | Down-regulated | protein_coding | tumor protein p53 inducible protein 11 [Source:HGNC Symbol;Acc:16842] |
| NANOS1 | Up-regulated | protein_coding | clathrin, light chain B [Source:HGNC Symbol;Acc:2091] |
| ZDHHC9 | Up-regulated | protein_coding | target of myb1-like 2 (chicken) [Source:HGNC Symbol;Acc:11984] |
| PRELP | Down-regulated | protein_coding | tubulin, beta 6 class V [Source:HGNC Symbol;Acc:20776] |
| RELN | Down-regulated | protein_coding | lamin B2 [Source:HGNC Symbol;Acc:6638] |
| APOD | Up-regulated | protein_coding | BCL2-related ovarian killer [Source:HGNC Symbol;Acc:1087] |
| H1F0 | Up-regulated | protein_coding | brain abundant, membrane attached signal protein 1 [Source:HGNC Symbol;Acc:957] |
| HN1 | Down-regulated | protein_coding | thymidylate synthetase [Source:HGNC Symbol;Acc:12441] |
| KAZN | Up-regulated | protein_coding | EPS8-like 2 [Source:HGNC Symbol;Acc:21296] |
| S100A4 | Down-regulated | protein_coding | Parkinson disease 7 domain containing 1 [Source:HGNC Symbol;Acc:26616] |
| TUBB | Down-regulated | protein_coding | PRKC, apoptosis, WT1, regulator [Source:HGNC Symbol;Acc:8614] |
| AFAP1 | Down-regulated | protein_coding | polymerase I and transcript release factor [Source:HGNC Symbol;Acc:9688] |
| CACNA1H | Down-regulated | protein_coding | polymerase (RNA) II (DNA directed) polypeptide L, 7.6kDa [Source:HGNC Symbol;Acc:9199] |
| MYO6 | Down-regulated | protein_coding | phosphodiesterase 4D interacting protein [Source:HGNC Symbol;Acc:15580] |
| MKL1 | Down-regulated | protein_coding | plectin [Source:HGNC Symbol;Acc:9069] |
| MMP1 | Up-regulated | protein_coding | frizzled family receptor 2 [Source:HGNC Symbol;Acc:4040] |
| ADH1B | Down-regulated | protein_coding | mab-21-like 1 (C. elegans) [Source:HGNC Symbol;Acc:6757] |
| DAPK1 | Down-regulated | protein_coding | gremlin 2, DAN family BMP antagonist [Source:HGNC Symbol;Acc:17655] |
| SCN8A | Down-regulated | protein_coding | oxytocin receptor [Source:HGNC Symbol;Acc:8529] |
| PDLIM7 | Down-regulated | protein_coding | RAP2B, member of RAS oncogene family [Source:HGNC Symbol;Acc:9862] |
| FLNA | Down-regulated | protein_coding | chromosome 5 open reading frame 30 [Source:HGNC Symbol;Acc:25052] |
| SRGAP1 | Down-regulated | protein_coding | exostosin glycosyltransferase 1 [Source:HGNC Symbol;Acc:3512] |
| ANXA6 | Down-regulated | protein_coding | Tyrosine-protein kinase SgK223 [Source:UniProtKB/Swiss-Prot;Acc:Q86YV5] |
| MAFG | Up-regulated | protein_coding | calcium channel, voltage-dependent, beta 4 subunit [Source:HGNC Symbol;Acc:1404] |
| SLC22A4 | Up-regulated | protein_coding | trafficking protein, kinesin binding 1 [Source:HGNC Symbol;Acc:29947] |
| SVIL | Up-regulated | protein_coding | annexin A2 [Source:HGNC Symbol;Acc:537] |
| PELI1 | Up-regulated | protein_coding | gap junction protein, gamma 1, 45kDa [Source:HGNC Symbol;Acc:4280] |
| MFAP5 | Down-regulated | protein_coding | solute carrier family 8 (sodium/calcium exchanger), member 1 [Source:HGNC Symbol;Acc:11068] |
| DPP4 | Up-regulated | protein_coding | growth arrest-specific 6 [Source:HGNC Symbol;Acc:4168] |
| PSAP | Up-regulated | protein_coding | collagen and calcium binding EGF domains 1 [Source:HGNC Symbol;Acc:29426] |
| SGTB | Down-regulated | protein_coding | family with sequence similarity 101, member B [Source:HGNC Symbol;Acc:28705] |
| MYO1C | Down-regulated | protein_coding | IQ motif containing GTPase activating protein 3 [Source:HGNC Symbol;Acc:20669] |
| FCHSD1 | Down-regulated | protein_coding | actin, gamma 1 [Source:HGNC Symbol;Acc:144] |
| LPAR1 | Up-regulated | protein_coding | protein kinase D1 [Source:HGNC Symbol;Acc:9407] |
| PIM3 | Up-regulated | protein_coding | colony stimulating factor 1 (macrophage) [Source:HGNC Symbol;Acc:2432] |
| SPRED2 | Up-regulated | protein_coding | transmembrane protein 173 [Source:HGNC Symbol;Acc:27962] |
| WWP2 | Down-regulated | protein_coding | mucin 1, cell surface associated [Source:HGNC Symbol;Acc:7508] |
| NTRK1 | Up-regulated | protein_coding | AHNAK nucleoprotein 2 [Source:HGNC Symbol;Acc:20125] |
| TPM2 | Down-regulated | protein_coding | phosphate cytidylyltransferase 2, ethanolamine [Source:HGNC Symbol;Acc:8756] |
| C2CD4A | Up-regulated | protein_coding | B-cell CLL/lymphoma 9-like [Source:HGNC Symbol;Acc:23688] |
| ITGBL1 | Down-regulated | protein_coding | kinesin family member 18B [Source:HGNC Symbol;Acc:27102] |
| PAPSS2 | Down-regulated | protein_coding | neurofibromin 2 (merlin) [Source:HGNC Symbol;Acc:7773] |
| DLL1 | Up-regulated | protein_coding | microphthalmia-associated transcription factor [Source:HGNC Symbol;Acc:7105] |
| ZNF521 | Up-regulated | protein_coding | glucosaminyl (N-acetyl) transferase 1, core 2 [Source:HGNC Symbol;Acc:4203] |
| ALPK2 | Down-regulated | protein_coding | formin binding protein 1 [Source:HGNC Symbol;Acc:17069] |
| GK | Up-regulated | protein_coding | sterile alpha motif domain containing 11 [Source:HGNC Symbol;Acc:28706] |
| ARHGAP11A | Down-regulated | protein_coding | platelet endothelial aggregation receptor 1 [Source:HGNC Symbol;Acc:33631] |
| GRK5 | Up-regulated | protein_coding | chromosome 6 open reading frame 132 [Source:HGNC Symbol;Acc:21288] |
| PRC1 | Down-regulated | protein_coding | tubulin, beta 4B class IVb [Source:HGNC Symbol;Acc:20771] |
| L1CAM | Down-regulated | protein_coding | chromosome 15 open reading frame 52 [Source:HGNC Symbol;Acc:33488] |
| SREBF2 | Down-regulated | protein_coding | proline/arginine-rich end leucine-rich repeat protein [Source:HGNC Symbol;Acc:9357] |
| RPL39 | Up-regulated | protein_coding | reelin [Source:HGNC Symbol;Acc:9957] |
| SAMD5 | Up-regulated | protein_coding | hematological and neurological expressed 1 [Source:HGNC Symbol;Acc:14569] |
| COL15A1 | Up-regulated | protein_coding | S100 calcium binding protein A4 [Source:HGNC Symbol;Acc:10494] |
| LAYN | Down-regulated | protein_coding | tubulin, beta class I [Source:HGNC Symbol;Acc:20778] |
| HLA-C | Up-regulated | protein_coding | actin filament associated protein 1 [Source:HGNC Symbol;Acc:24017] |
| PTCHD2 | Up-regulated | protein_coding | calcium channel, voltage-dependent, T type, alpha 1H subunit [Source:HGNC Symbol;Acc:1395] |
| PSG5 | Down-regulated | protein_coding | myosin VI [Source:HGNC Symbol;Acc:7605] |
| LGR4 | Down-regulated | protein_coding | megakaryoblastic leukemia (translocation) 1 [Source:HGNC Symbol;Acc:14334] |
| TECPR1 | Down-regulated | protein_coding | alcohol dehydrogenase 1B (class I), beta polypeptide [Source:HGNC Symbol;Acc:250] |
| C2CD4B | Up-regulated | protein_coding | death-associated protein kinase 1 [Source:HGNC Symbol;Acc:2674] |
| ATP10A | Down-regulated | protein_coding | sodium channel, voltage gated, type VIII, alpha subunit [Source:HGNC Symbol;Acc:10596] |
| HLA-H | Up-regulated | pseudogene | PDZ and LIM domain 7 (enigma) [Source:HGNC Symbol;Acc:22958] |
| HLA-A | Up-regulated | protein_coding | filamin A, alpha [Source:HGNC Symbol;Acc:3754] |
| PTPLB | Down-regulated | protein_coding | SLIT-ROBO Rho GTPase activating protein 1 [Source:HGNC Symbol;Acc:17382] |
| VGLL3 | Down-regulated | protein_coding | annexin A6 [Source:HGNC Symbol;Acc:544] |
| LBH | Down-regulated | protein_coding | microfibrillar associated protein 5 [Source:HGNC Symbol;Acc:29673] |
| EMP2 | Up-regulated | protein_coding | small glutamine-rich tetratricopeptide repeat (TPR)-containing, beta [Source:HGNC Symbol;Acc:23567] |
| ITGA1 | Down-regulated | protein_coding | myosin IC [Source:HGNC Symbol;Acc:7597] |
| FER1L6 | Up-regulated | protein_coding | FCH and double SH3 domains 1 [Source:HGNC Symbol;Acc:25463] |
| KRTAP1-5 | Down-regulated | protein_coding | WW domain containing E3 ubiquitin protein ligase 2 [Source:HGNC Symbol;Acc:16804] |
| LINC00152 | Down-regulated | lincRNA | tropomyosin 2 (beta) [Source:HGNC Symbol;Acc:12011] |
| LINC00473 | Up-regulated | processed_transcript | integrin, beta-like 1 (with EGF-like repeat domains) [Source:HGNC Symbol;Acc:6164] |
| RP11-48O20.4 | Down-regulated | lincRNA | 3'-phosphoadenosine 5'-phosphosulfate synthase 2 [Source:HGNC Symbol;Acc:8604] |
| ARHGAP23 | Down-regulated | protein_coding | alpha-kinase 2 [Source:HGNC Symbol;Acc:20565] |
| SLC26A6 | Up-regulated | protein_coding | Rho GTPase activating protein 11A [Source:HGNC Symbol;Acc:15783] |
| C14orf132 | Up-regulated | protein_coding | protein regulator of cytokinesis 1 [Source:HGNC Symbol;Acc:9341] |
| RP11-206L10.11 | Up-regulated | processed_transcript | L1 cell adhesion molecule [Source:HGNC Symbol;Acc:6470] |
| NAMPTL | Up-regulated | protein_coding | sterol regulatory element binding transcription factor 2 [Source:HGNC Symbol;Acc:11290] |
| TWIST2 | Up-regulated | protein_coding | layilin [Source:HGNC Symbol;Acc:29471] |
| JRK | Down-regulated | processed_transcript | pregnancy specific beta-1-glycoprotein 5 [Source:HGNC Symbol;Acc:9522] |
| GAS5 | Up-regulated | processed_transcript | leucine-rich repeat containing G protein-coupled receptor 4 [Source:HGNC Symbol;Acc:13299] |
| HLA-B | Up-regulated | protein_coding | tectonin beta-propeller repeat containing 1 [Source:HGNC Symbol;Acc:22214] |
| C12orf75 | Down-regulated | protein_coding | ATPase, class V, type 10A [Source:HGNC Symbol;Acc:13542] |
| TRHDE-AS1 | Down-regulated | processed_transcript | protein tyrosine phosphatase-like (proline instead of catalytic arginine), member b [Source:HGNC Symbol;Acc:9640] |
| BCYRN1 | Down-regulated | lincRNA | vestigial like 3 (Drosophila) [Source:HGNC Symbol;Acc:24327] |
| KIFC1 | Down-regulated | protein_coding | limb bud and heart development [Source:HGNC Symbol;Acc:29532] |
| C8orf58 | Down-regulated | protein_coding | integrin, alpha 1 [Source:HGNC Symbol;Acc:6134] |
| PEG10 | Down-regulated | protein_coding | keratin associated protein 1-5 [Source:HGNC Symbol;Acc:16777] |
| MICAL3 | Down-regulated | protein_coding | long intergenic non-protein coding RNA 152 [Source:HGNC Symbol;Acc:28717] |
| RPLP0P2 | Up-regulated | pseudogene | - |
| CTC-463N11.3 | Down-regulated | antisense | Rho GTPase activating protein 23 [Source:HGNC Symbol;Acc:29293] |
| STARD4-AS1 | Down-regulated | processed_transcript | jerky homolog (mouse) [Source:HGNC Symbol;Acc:6199] |
| MIR143HG | Down-regulated | processed_transcript | chromosome 12 open reading frame 75 [Source:HGNC Symbol;Acc:35164] |
| TMEM158 | Up-regulated | protein_coding | TRHDE antisense RNA 1 [Source:HGNC Symbol;Acc:27471] |
| KIAA1456 | Down-regulated | protein_coding | brain cytoplasmic RNA 1 [Source:HGNC Symbol;Acc:1022] |
| TRNP1 | Down-regulated | protein_coding | kinesin family member C1 [Source:HGNC Symbol;Acc:6389] |
| CHMP1B | Up-regulated | protein_coding | chromosome 8 open reading frame 58 [Source:HGNC Symbol;Acc:32233] |
| CTSO | Up-regulated | protein_coding | paternally expressed 10 [Source:HGNC Symbol;Acc:14005] |
| RP11-244F12.3 | Down-regulated | antisense | microtubule associated monooxygenase, calponin and LIM domain containing 3 [Source:HGNC Symbol;Acc:24694] |
| RP4-659J6.2 | Up-regulated | antisense | - |
| CTD-3252C9.4 | Down-regulated | lincRNA | STARD4 antisense RNA 1 [Source:HGNC Symbol;Acc:44117] |
| KCNQ1OT1 | Down-regulated | antisense | MIR143 host gene (non-protein coding) [Source:HGNC Symbol;Acc:42872] |
| hsa-mir-145 | Down-regulated | lincRNA | KIAA1456 [Source:HGNC Symbol;Acc:26725] |

**Table S4. Experiment treatments**

| Group | Number of animals | Dose concentration  (cells/mL) | Dose of injection  (μL) | Indication |
| --- | --- | --- | --- | --- |
| Negative control | 4 | / | / | Empty treatment |
| Hanks control | 4 | / | 200 | Hanks solution |
| P5 FBs | 4 | 1×10^7^ | 200 | FBs at passage 5 |
| P5 tSKPs | 4 | 1×10^7^ | 200 | tSKPs generated from FBs at passage 5 |
| P5 FBs+tSKPs | 4 | 1×10^7^ | 200 | a mixture of P5 FBs and tSKPs at 1:1 cell ratio |
| P10 FBs | 4 | 1×10^7^ | 200 | FBs at passage 10 |
| P10 tSKPs | 4 | 1×10^7^ | 200 | tSKPs generated from FBs at passage 10 |
| P10 FBs+tSKPs | 4 | 1×10^7^ | 200 | a mixture of P5 FBs and tSKPs at 1:1 cell ratio |

FB, fibroblast; tSKPs, trans-cultured skin-derive
